# Naturally occurring variations in the nod-independent model legume *Aeschynomene evenia* and relatives: a resource for nodulation genetics

**DOI:** 10.1186/s12870-018-1260-2

**Published:** 2018-04-03

**Authors:** Clémence Chaintreuil, Xavier Perrier, Guillaume Martin, Joël Fardoux, Gwilym P. Lewis, Laurent Brottier, Ronan Rivallan, Mario Gomez-Pacheco, Mickaël Bourges, Léo Lamy, Béatrice Thibaud, Heriniaina Ramanankierana, Herizo Randriambanona, Hervé Vandrot, Pierre Mournet, Eric Giraud, Jean-François Arrighi

**Affiliations:** 1 IRD, Laboratoire des Symbioses Tropicales et Méditerranéennes, UMR LSTM, Campus International de Baillarguet, F-34398 Montpellier, France; 2 CIRAD, Amélioration Génétique et Adaptation des Plantes Méditerranéennes et Tropicales, UMR AGAP, Campus de Lavalette, F-34398 Montpellier, France; 3 AGAP, Univ. Montpellier, CIRAD, INRA, Montpellier SupAgro, Montpellier, France; 4 Comparative Plant and Fungal Biology Department, Royal Botanic Gardens, Kew, Richmond, Surrey, TW9 3AB UK; 5 Institute of Integrative Biology of the Cell (I2BC), CEA, CNRS, Univ. Paris-Sud. Université Paris-Saclay, 91198 Gif-sur-Yvette, France; 6 Laboratoire de Microbiologie de l’Environnement/Centre National de Recherche sur l’Environnement, 101 Antananarivo, Madagascar; 7 IAC, Laboratoire de Botanique et d’Ecologie Végétale Appliquée, UMR AMAP, 98825 Pouembout, Nouvelle-Calédonie, France; 8 LSTM, Univ. Montpellier, CIRAD, INRA, IRD, Montpellier SupAgro, Montpellier, France

**Keywords:** *Aeschynomene*, Diversity, Genotype, Legume, Nodulation, Ploidy, Species, Symbiosis

## Abstract

**Background:**

Among semi-aquatic species of the legume genus *Aeschynomene*, some have the unique property of being root and stem-nodulated by photosynthetic *Bradyrhizobium* lacking the *nodABC* genes necessary for the production of Nod factors. These species provide an excellent biological system with which to explore the evolution of nodulation in legumes. Among them, *Aeschynomene evenia* has emerged as a model legume to undertake the genetic dissection of the so-called Nod-independent symbiosis. In addition to the genetic analysis of nodulation on a reference line, natural variation in a germplasm collection could also be surveyed to uncover genetic determinants of nodulation. To this aim, we investigated the patterns of genetic diversity in a collection of 226 Nod-independent *Aeschynomene* accessions.

**Results:**

A combination of phylogenetic analyses, comprising *ITS* and low-copy nuclear genes, along with cytogenetic experiments and artificial hybridizations revealed the richness of the Nod-independent *Aeschynomene* group with the identification of 13 diploid and 6 polyploid well-differentiated taxa. A set of 54 SSRs was used to further delineate taxon boundaries and to identify different genotypes. Patterns of microsatellite diversity also illuminated the genetic basis of the *Aeschynomene* taxa that were all found to be predominantly autogamous and with a predicted simple disomic inheritance, two attributes favorable for genetics. In addition, taxa displaying a pronounced genetic diversity, notably *A. evenia*, *A. indica* and *A. sensitiva*, were characterized by a clear geographically-based genetic structure and variations in root and stem nodulation.

**Conclusion:**

A well-characterized germplasm collection now exists as a major genetic resource to thoroughly explore the natural variation of nodulation in response to different bradyrhizobial strains. Symbiotic polymorphisms are expected to be found notably in the induction of nodulation, in nitrogen fixation and also in stem nodulation. Subsequent genetic analysis and locus mapping will pave the way for the identification of the underlying genes through forward or reverse genetics. Such discoveries will significantly contribute to our understanding of the molecular mechanisms underpinning how some *Aeschynomene* species can be efficiently nodulated in a Nod-independent fashion.

**Electronic supplementary material:**

The online version of this article (10.1186/s12870-018-1260-2) contains supplementary material, which is available to authorized users.

## Background

The legume family (*Leguminosae*) accounts for ~ 27% of the world’s primary crop production and is second only to cereals in economic and nutritional value. It includes many crops of agronomic importance for grain production, pasture and agroforestry. Many legumes are pioneers plants improving soil fertility and moderating harsh environments. Such economic and ecological success of the legume family is, in large part, due to the ability of the vast majority of its 20,000 species to develop symbiotic interactions with nitrogen-fixing bacteria collectively referred as rhizobia [1]. In this symbiosis, the rhizobia produce signal molecules, the Nod factors, whose specific recognition by the host plant is necessary to activate the formation of root nodules that correspond to symbiotic organs where the rhizobia are hosted. Inside the nodules, the rhizobia reduce atmospheric nitrogen (N_2_) into ammonium (NH_4_^+^), a form of nitrogen that is usable by the plant for its development. Historically, two model legumes, *Medicago truncatula* and *Lotus japonicus*, have been used to genetically investigate this nodulation process. Such studies have resulted in the identification and elucidation of the role of many genes that are essential for the different steps of nodule development and its infection by the symbiont [2–4]. To broaden our understanding of the molecular mechanisms underlying the nitrogen-fixing symbiosis, there has arisen a fast growing interest in uncovering the diversity of nodulation processes that are found in other legume species [5, 6].

In this line, the mainly tropical legume genus *Aeschynomene* represents a group of prime interest as it contains several original symbiotic features. The genus *Aeschynomene* was originally known for the ability of different species to develop stem nodules in addition to the typical root nodules. Stem nodulation is uncommon in legumes, being shared with a very few hydrophytic species of the genera *Sesbania*, *Neptunia* and *Discolobium*, but it is widespread among the semi-aquatic *Aeschynomene* species [7–9]. In addition, some bradyrhizobia isolated from *Aeschynomene* stem nodules exhibit a photosynthetic activity that was shown to play a key role in stem nodules by directly furnishing energy to the bacterium that can be used for biological nitrogen fixation [10, 11]. Even more outstanding was the discovery that some of these photosynthetic bradyrhizobia lack both the canonical *nodABC* genes required for the synthesis of the key Nod factors and a type III secretion system (T3SS) that is known in other rhizobia to activate or modulate nodulation [12–14]. This led to a new paradigm in nodulation studies in which an alternative symbiotic process between rhizobia and legumes efficiently triggers nodule formation in a Nod (factor)-independent fashion.

Phylogenetic analysis of the genus *Aeschynomene* revealed that all the species endowed with a Nod-independent nodulation process cluster in a single clade where no species using a Nod-dependent symbiotic process are found [8]. A taxonomic revision of American *Aeschynomene* was published in 1955 by Rudd [15], but it predated phylogenetic studies in plants and included no species native outside the Neotropics. First molecular studies of *Aeschynomene* pointed to new cryptic taxa differing by their ploidy levels [16, 17]. The knowledge gained from the study of the Nod-independent clade was also used to select *Aeschynomene evenia* as a new model legume for the purpose of deciphering the molecular mechanisms of the Nod-independent symbiosis [16, 18]. Key attributes of this species include its small, diploid genome (2n = 20, 415 Mb/1C), its selfing nature and its prolific seed production. Several tools have been developed including artificial hybridization and the *Agrobacterium rhizogenes*-mediated root transformation, rendering this species ideal for molecular genetic studies. First insights were obtained from RNAseq analysis and reverse genetics by revealing that some symbiotic determinants identified in *Medicago* and *Lotus* are recruited in the Nod-independent process but several key genes involved in bacterial recognition, symbiotic infection and nodule functioning were found not to be expressed during root nodulation [9, 19–21].

Forward genetics are now expected to allow the identification of the specific molecular determinants of the Nod-independent process in *A. evenia*. To optimize research effort, a reference line was inbred and successfully used to generate an SSR-based genetic map of *A. evenia* [9]. This genetic map uncovered the genome structure and the distribution of symbiotic genes. It also provides a basis for a genome sequencing project and paves the way of the genetic dissection of nodulation in this reference line. This does not exclude exploring the naturally occurring variations in nodulation, as a complementary genetic approach, in order to increase our understanding of symbiotic gene functions and of the genetic control of symbiosis as successfully performed in other legumes such as *Medicago*, *Lotus* and soybean [22]. But exploiting genetic diversity requires prior knowledge of the extent and structure of the variations occurring in the species of interest. Although the genetic relationships among the Nod-independent *Aeschynomene* species have been analysed using molecular markers, only two studies have included a small set of accessions for the diploid *A. evenia* and the related polyploid *A. indica* [17, 18]. As a consequence, the variations within and among the species of the Nod-independent clade remain largely uncharacterized.

To enable an efficient use of the natural variation in genetic studies of nodulation, we surveyed the genetic diversity occurring in a collection of 226 Nod-independent *Aeschynomene* accessions spanning the whole distributional range of this clade. As a first step, genetic relationships and differentiation between Nod-independent *Aeschynomene* taxa were established using a combination of molecular phylogenies, cytogenetics and hybridization experiments. This information then served as supportive data for the analysis of genotype data obtained for the germplasm collection using a set of 54 SSR markers. Patterns of microsatellite diversity illuminated the genetic basis of the *Aeschynomene* taxa and uncovered their genetic differentiation. The presence of an underlying genetic structure was then compared with geographical distribution data.

## Results

### Species identification and relationships

**Fig. 1:**
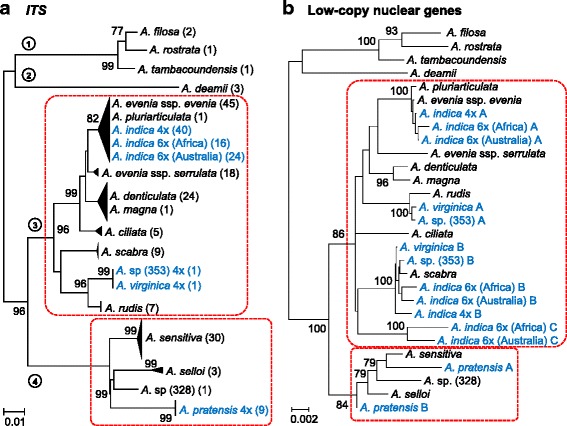
Phylogenetic and genetic relationships in the Nod-independent *Aeschynomene* clade. Phylogenetic reconstructions were obtained using the Neighbor Joining method. **a** *ITS* phylogeny. Accessions with ITS sequence divergence < 1% were clustered together. Numbers of accessions per taxon are indicated in brackets. **b** Phylogeny based on 5 concatenated low-copy nuclear gene fragments: *CYP1*, *eiF1α*, *SUI1*, *SuSy* and a gene homolog to Glyma.07G136800 and Glyma.18G187300. -A, -B and -C indicate the different copies found in polyploid species. The four gene pools are identified with a circled number and the *A. evenia and A. sensitiva* groups are framed in a box bordered with a dashed red line. Diploid taxa are in black and polyploid taxa in blue with ploidy level indicated. Numbers at nodes represent bootstrap values (% of 1000 replicates)

A collection of 233 accessions was developed to investigate the phylogenetic relationships and the genetic differentiation in the Nod-independent *Aeschynomene* clade (Additional file 1: Table S1). It included all the known species included in the clade and aimed to cover their distributional range [15, 23, 24]. The nuclear ribosomal *ITS* region was used as a marker of the species identity and served to reconstruct a phylogeny of the whole group based on the Neighbor Joining (NJ) method (Additional files 2 and 3: Tables S2 and S3). To simplify the resulting tree, accessions showing less than 1% of divergence in their sequence were grouped in the same clade (Fig. 1a, Additional file 4: Doc. S1). The *ITS* tree was composed of four lineages: one grouping *A. filosa*, *A. rostrata* and *A. tambacoundensis*, a monospecific one with *A. deamii*, a third one comprising *A. evenia* and its sister species, and a fourth one containing *A. sensitiva* and related species (Fig. 1a).

In this phylogenetic tree, a number of putative species displayed the same *ITS* sequence. In particular, two rare species, *A. magna* and *A. pluriarticulata*, tightly clustered with two widespread species, *A. denticulata* and *A. evenia*, respectively (Fig. 1a). Similarly, *A. evenia* and *A. indica* shared the same *ITS* signature but they were previously shown to form a species complex containing three cytotypes (2×, 4× and 6×) [17]. To sort these, accessions of this species complex were genotyped with SSR markers that are indicators of their genome constitution [17]. This led to the additional discovery that the *A. indica* 6× accessions of African and Australian origin displayed different SSR profiles, justifying their distinction in the phylogenic tree (Fig. 1a, data not shown). It is also noteworthy that two accessions did not fit well with the description of any known *Aeschynomene* species and so we refer to these as *Aeschynomene* sp. (328) and *A*. sp. (353) (Fig. 1a, Additional file 1: Table S1). To clarify the genetic status of these putative new taxa, they were both included in a flow cytometry analysis and a chromosome count (Additional file 1: Table S1, Additional file 5: Figure S1). Cytogenetic data were mapped onto the phylogeny, showing that *Aeschynomene* sp. (328) represented a new 2× taxon while *A*. sp. (353) was a 4× taxon, similar to the closely related *A. virginica*, and confirming the hexaploid status of the Australian *A. indica* 6× (Fig. 1a).

**Fig. 2:**
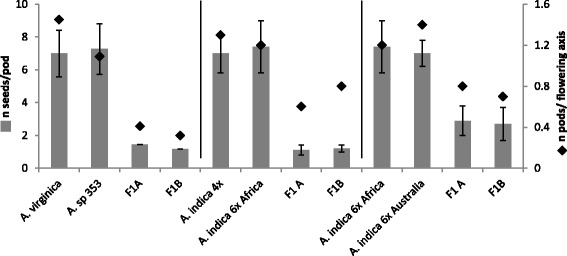
Hybridization experiments between related taxa. Manual crosses were performed between *A. virginica* and *A*. sp (353), *A. indica* 4× and *A. indica* 6× -Africa-, *A. indica* 6× -Africa- and *A. indica* 6× -Australia. Plant fertility was evaluated for the parental taxa and two independently obtained F1 hybrids based on the number of seeds produced per pod and the number of developed pods per flowering axis. Error bars represent s.d. (*n* = 30)

To further uncover genetic relationships between taxa, five low copy nuclear genes -*CYP1* (Cyclophilin 1), *eiF1α* (eukaryotic translation initiation factor α), *SUI1* (translation factor), *SuSy* (Sucrose Synthase) and a gene homolog to Glyma.07G136800 and Glyma.18G187300 identified in *Glycine max* - were cloned and sequenced in selected accessions (Additional file 2: Table S2). For diploid species, single sequences were obtained, while for polyploid species homeologous sequences were isolated for almost all genes (Additional file 3: Table S3). The five genes treated separately gave similar NJ trees where the homeologous sequences for polyploid taxa could be differentiated based on the differential clustering with the sequences of diploid taxa (not shown). To provide a unique and well-resolved NJ phylogeny, the gene sequences were concatenated together (Fig. 1b). In the resulting tree, the topology of the branches containing the diploid species was similar to that of the *ITS* tree, corroborating the distinctness of *A.* sp. (328) from other known *Aeschynomene* species (Fig. 1a,b). For the polyploid taxa, the different genome components were scattered different part of the phylogeny, revealing that the two taxa *A*. sp. (353) and *A. virginica*, and the African and Australian *A. indica* 6× had the same or a similar genomic constitution (Fig. 1b). To assess the genetic differentiation of these related taxa, they were manually crossed: *A*. sp. (353) with *A. virginica*, the African *A. indica* 6× with the Australian *A. indica* 6× and *A. indica* 4× with *A. indica* 6× for comparison (Fig. 2). In all cases, hybrid plants were obtained but they greatly differed in their fertility compared to their respective parental accessions, as seen by a drastic reduction of both the number of developed pods per flowering axis and of seeds per pod. As a result, these data suggest that the tested taxa are truly different *Aeschynomene* species.

### Genetic patterns and behaviours

To determine the genetic structure of the genomes of different taxa, we conducted a SSR genotyping of the collection of *Aeschynomene* accessions. For this, we tested the set of 500 SSRs previously used when developing a genetic map for *A. evenia* [9]. These markers were screened for polymorphism in four samples that are genetically different: two accessions of *A. evenia* 2× (the reference line CIAT 22838 and the mapping parent CIAT 8232), one accession of *A. indica* 4× (PI 196206) and one of *A. indica* 6× (LSTM19). Of these, 64 markers were selected using two main criteria: (1) the requirement to be polymorphic between the two *A. evenia* accessions so as to avoid the use of invariant SSRs, (2) to amplify a single allele in the 2× accessions, 2 and 3 alleles in the 4× and 6× accessions, respectively, with the assumption that the distinct alleles came from the different genomic components of the polyploid species (Additional files 6, 7 and 8: Figure S2, Tables S4 and S5). Forty nine out of the 64 selected markers were previously positioned on different *A. evenia* linkage groups, with their distribution representing a reasonable coverage of the *A. evenia* genetic map (Additional file 7: Table S4).

Preliminary genotyping experiments revealed that the SSRs developed from *A. evenia* sequences had a transferability rate qualified as (i) modest with *A. deamii* and *A. tambacoundensis*, (ii) good with *A. sensitiva* and *A. pratensis* and (iii) high with *A. denticulata* and *A. scabra*. These data were in accordance with the phylogenetic distance of the different gene pools relative to *A. evenia* (Fig. 1), but this prompted us to restrict our analysis to the *A. evenia* and *A. sensitiva* groups. The corresponding accessions were then subjected to high-throughput SSR genotyping by capillary sequencing (Additional file 9: Figure S3). The absence of amplification for some of them in the *A. sensitiva* group was compensated by the use of an additional set of SSR markers. Of the 64 SSR markers, 54 generated clearly interpretable allele profiles in at least one of the two groups, 38 SSR markers for the *A. evenia* group and 46 SSR markers for the *A. sensitiva* group, with 30 of them in common (Additional file 6: Figure S2). Allelic diversity, estimated by the number of alleles *Na*, varied importantly among the SSR markers used with an average of 12.8 alleles per locus in the *A. evenia* group (*n* = 186 accessions) and 4.21 in the *A. sensitiva* group (*n* = 40 accession) (Additional file 10: Table S6). Observed heterozygosities were very low for each SSR (*Ho* < 0.1 with one exception at 0.2), indicating that they amplified at single locus for diploid accessions and homeologous loci for polyploid accessions (Additional file 10: Table S6).

These results supported the appropriateness of these SSRs for investigating the genetic properties of species. Therefore they were analysed in a second step to characterize the taxa. Transferability level of the SSR markers from *A. evenia*, ranged from 92 to 100% for other taxa of the *A. evenia* group and from 72 to 78% for those of the *A. sensitiva* group (Table 1, Additional file 11: Table S7). Analysing the *Na* parameter revealed different levels of genetic variation among the *Aeschynomene* taxa, one of the highest being found in *A. evenia* (mean 5.2 alleles per SSR) (Table 1, Additional file 10: Table S6). These values reflected the genetic diversity of the taxa, but they were likely to have been influenced by the marked variation in sample sizes and by the non-random selection of the SSR markers. The mean number of alleles detected for the SSR markers in each taxon were congruent with their ploidy levels, with (i) 1.00 to 1.06 alleles observed for diploid taxa, (ii) 1.42 to 1.92 alleles for tetraploid taxa and (iii) 2.63 to 2.84 alleles for hexaploid taxa (Table 1, Additional file 12: Table S8). Accessions displaying more alleles than expected from their ploidy level were considered as heterozygous (Additional files 1 and 13: Tables S1, S9). Selfing rates based on the observed heterozygosities, ranged from 94.5% to 100%, with an average 98.7% score for *A. evenia* (Table 1, Additional file 13: Table S9). This provided genetic support for previous observations that the Nod-independent *Aeschynomene* species are preferentially autogamous [17, 18].

**Table 1:** Summary of the data obtained for the *Aeschynomene* taxa

Taxa/ ploidy level	n samples	n genotypes	Cross-species transferability	*NA*	n co-present alleles/SSR	*Ho*	Comment
2× taxa							
*A. ciliata*	5		100%	1.2	1.00	0.000	
*A. deamii*	3		–	–	–	–	
*A. denticulata*	24	3	92%	2.7	1.06	0.055	*A. magna* conspecific
*A. evenia* ssp. *evenia*	44	7	–	5.2	1.01	0.013	*A. pluriarticulata* conspecific
*A. evenia* ssp. *serrulata*	15	2	100%	2.3	1.02	0.019	
*A. filosa*	2		–	–	–	–	
*A. rostrata*	1		–	–	–	–	
*A. rudis*	7		95%	1.9	1.00	0.004	
*A. scabra*	8		100%	1.6	1.00	0.000	
*A. selloi*	3		78%	1.1	1.00	0.000	
*A. sensitiva*	27	4	78%	2.6	1.03	0.028	
*A. sp* (328)	1		72%	1	1.00	0.000	new taxon
*A. tambacoundensis*	1		–	–	–	–	
4× taxa							
*A. indica* 4×	41	6	100%	4.9	1.92	0.019	
*A. pratensis*	9		78%	2.1	1.60	0.000	
*A. sp* (353)	1		100%	1.6	1.66	0.000	new taxon
*A. virginica*	1		100%	1.5	1.42	0.000	
6× taxa							
*A. indica* 6× Africa	16	2	100%	4.8	2.84	0.005	
*A. indica* 6× Australia	24	3	100%	6.9	2.63	0.002	new taxon

### Genetic diversity and genotype delineation

**Fig. 3:**
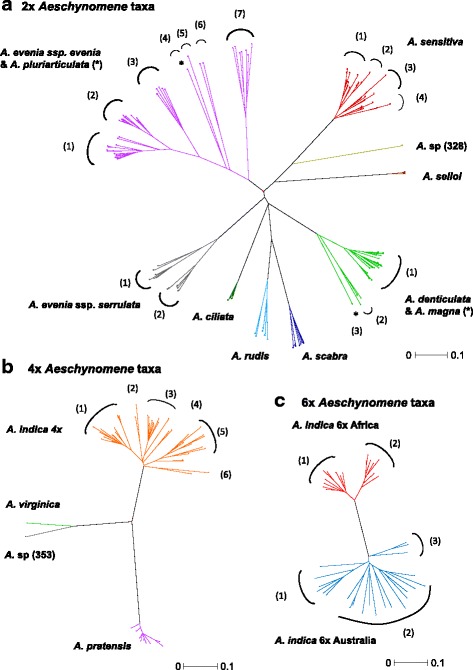
NJ trees representing the genetic diversity among the Nod-independent *Aeschynomene* accessions. The trees were developed separately in DARWIN using the allelic data of 54 SSRs for the 2× (**a**), 4× (**b**) and 6× (**c**) taxa. Well-differentiated taxa are distinctly coloured and identified genotypes are numbered. Species suspected to be morphological variants are marked with an asterisk

Based on the geographical distributions of the accessions making up the different Nod-independent *Aeschynomen*e taxa, most of their genetic diversity and structure were expected to be uncovered. Therefore, the genotyping data obtained for the *A. evenia* and *A. sensitiva* groups were combined and used to estimate pair-wise distances between all accessions and thereby generate a dissimilarity matrix in DARwin v5 [25]. From this, NJ trees were calculated for the 2×, 4×, 6× ploidy levels separately. Indeed, alleles of the genomic components of polyploid accessions cannot be analysed separately and so their inclusion in the analysis of the diploid accessions would result in their grouping with only one of the potential progenitors. Considering the 2× NJ tree, well-separated clades were evident and corresponded to the known species *A. evenia*, *A. ciliata*, *A. denticulata*, *A. rudis*, *A. scabra*, *A. serrulata*, *A. selloi* and *A. sensitiva*, as well as the newly identified *A*. sp. (328) (Fig. 3a,Additional file 9: Figure S3). Interestingly, *A. pluriarticulata* was found to be nested within the *A. evenia* accessions and the accession of *A. magna* tightly clustered with accessions of *A. denticulata*. This information together with the phylogenetic and genetic relationships makes their taxonomic distinctness uncertain. Conversely, the tree topology showed a clear separation of the two *A. evenia* subspecies, *evenia* and *serrulata*, and several 2× taxa (*A. denticulata*, *A. evenia* ssp. *evenia*, *A. evenia* ssp. *serrulata* and *A. sensitiva*) could be subdivided in different clusters delineating genotypes (Fig. 3a,Additional file 9: Figure S3). Among these, genetic diversity and differentiation was the highest for *A. evenia* ssp. *evenia* (here after *A. evenia* s.s. in the text), with 7 genotypes identified. Regarding the 4× tree, *A. virginica* and *A*. sp (353) were found to form sister lineages and contrasted patterns of genetic differentiation were observed with a very low genetic variability noted for *A. pratensis* whereas *A. indica* 4× was composed of several well-diverged clusters (Fig. 3b,Additional file 9: Figure S3). In the 6× tree, the African and Australian *A. indica* 6× were genetically distant with the African set subdivided in two homogenous genotypes while the Australian set contained three genotypes that formed far more diverse assemblages, indicative of distinct evolutions (Fig. 3c, Additional file 9: Figure S3).

**Fig. 4:**
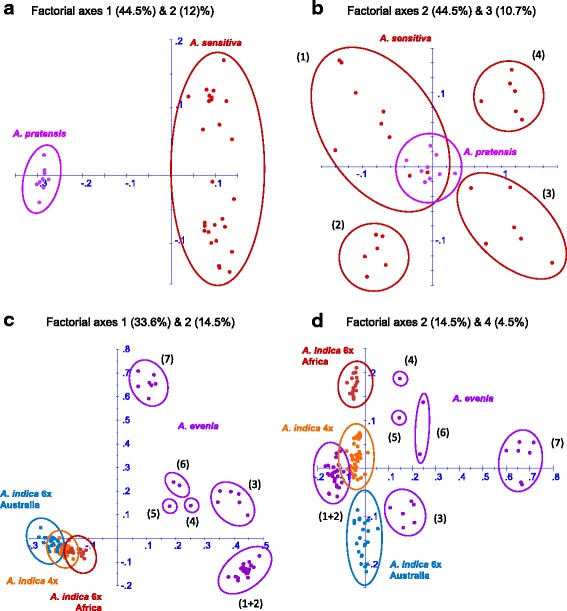
Factorial analysis of Nod-independent *Aeschynomene* taxa. (**a**) and (**b**) for the *A. evenia*-*A. indica* species complex, (**c**) and (**d**) for the *A. sensitiva*-*A. pratensis* species complex. Two pairss of factorial axes (with the percentage of variation they account for indicated in parenthesis) are used for each species complex so as to show genetic distinctness and relationships. Taxon colours and genotype numbers are the same as in Fig. 3

In a search for other relationships, a Factorial Analysis (FA) was also carried out in DARwin v5 [25]. This approach is more informative regarding distances among different groups and it also allows comparison of accessions of different ploidy levels. We focused on *A. evenia* and *A. sensitiva* to get a more detailed view of the genetic relatedness between the 2× genotypes and the derived polyploid taxa. FA clearly distinguished *A. sensitiva* and *A. pratensis* when using the factorial axes 1 and 2 (Fig. 4a). Factorial axes 2 and 3 separated the four *A. sensitiva* genotypes and but the central position of *A. pratensis* was interpreted has an absence of a preferential relationship with any of the *A. sensitiva* genotypes (Fig. 4b). Therefore, either the parental *A. sensitiva* genotype that contributed to the polyploid genome of *A. pratensis* was missing or *A. pratensis* was formed before the intraspecific differentiation of *A. sensitiva* as suggested by the sequence divergence of the nuclear genes (Fig. 1b). For *A. evenia* s.s., the FA separated the 7 identified genotypes but grouped the three *A. indica* taxa together when using factorial axes 1 and 2 (Fig. 4c). These polyploid taxa could be separated along factorial axis 4 and showed preferential affinity with the two unseparated genotypes (1) and (2) of *A. evenia* along factorial axis 2 (Fig. 4d). These observations suggested a common origin of the three *A. indica* taxa that would derive from the same *A. evenia* genome donor, this latter being potentially ancestral to the genotypes 1 and 2.

### Geographical structure of the Aeschynomene species

**Fig. 5:**
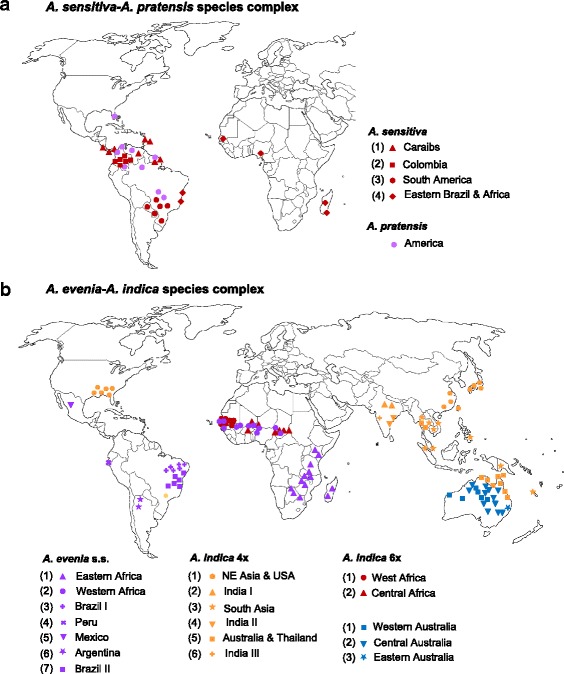
Geographical distribution of Nod-independent *Aeschynomene* taxa. (**a**) for the *A. sensitiva*-*A. pratensis* species complex and (**b**) for the *A. evenia*-*A. indica* species complex. Accessions with no geographical information are not shown; details of the accession origins are provided in Additional files 1 and 14: Table S1 and Figure S4. Taxon colours and genotype numbers are the same as in Fig. 3

The Nod-independent *Aeschynomene* clade is mainly a tropical/subtropical group, but the species show distinct geographical distributions [15, 23, 24]. Out of the 19 taxa considered here (Table 1), 13 are strictly American, while three taxa (*A. evenia*, *A. indica* 4× and *A. sensitiva*) have a wider distribution; two are African (*A. indica* 6× - Africa- and *A. tambacoundensis*) and one Australian (*A. indica* 6× - Australia-). This confirmed previous conclusions that America is the centre of origin and diversification of this clade but that several outliers have subsequently evolved in other continents [8]. Because some species showed a pronounced genetic differentiation and are part of polyploid species complexes, we investigated to what extent the corresponding cytotypes and genotypes are geographically structured. In the *A. sensitiva*-*A. pratensis* species complex, *A. sensitiva* has a transatlantic distribution. When mapping accessions globally, a clear geographical separation of the four *A. sensitiva* genotypes was observed. One occupies the Caribbean region, one found in Colombia, another one the central area of South America and, more noticeably a fourth one being present both in coastal East Brazil and in Africa, suggesting a recent dispersal event (Fig. 5a, Additional file 9: Figure S3, Additional file 1: Table S1). In contrast to *A. sensitiva*, the low genetic diversity observed in *A. pratensis* 4× revealed no consistent geographical pattern (Fig. 5A, Additional file 9: Figure S3, Additional file 1: Table S1).

In the pantropically distributed *A. evenia-A. indica* species complex, a prominent geographical division between the different taxa was observed. *A. evenia* s.s. (2×) grows both in America and Africa, *A. indica* 4× is widespread in Asia, including India, and it is also present in North Eastern America, while the two *A. indica* 6× taxa distinctly occur in Africa and Australia (Fig. 5b). At the intraspecific level, the identified genotypes were found to represent geographically defined groups. *A. evenia* displayed a high level of genetic structure with 5 American and 2 African genotypes. As far as phylogenetic relationships can be inferred from an SSR-based NJ tree, the African genotypes appeared to have diverged after a recent transatlantic migration from the Neotropics (Fig. 5b, Additional file 9: Figure S3, Additional file 1: Table S1). *A. indica* 4× contained 3 distinct Indian lineages, one genotype spanning Eastern Asia and the North Eastern America, a second widespread in Northern Australia but also occurring in Asia, and a third throughout South Asia and extending to the Pacific (Fig. 5b). The Australian *A. indica* 6× was found to have a wide distribution extending from Eastern to Western Australia. Missing collection data limited the analysis of the three 6× genotypes but they did not overlap in distributional area with *A. indica* 4× (Fig. 5b, Additional file 9: Figure S3, Additional file 1: Table S1). In contrast, distribution of the African *A. indica* 6× was restricted to the Subsahelian zone and the two identified genotypes clearly could be distinguished based on their location, one in the western part of the zone, the other one with a more central position (Fig. 5b, Additional file 9: Figure S3, Additional file 1: Table S1). Noteworthy, at a macroscale, there exists an overlap in distribution of African *A. indica* 6× with *A. evenia* s.s, but the latter was more widespread in Africa*.*

**Fig. 6:**
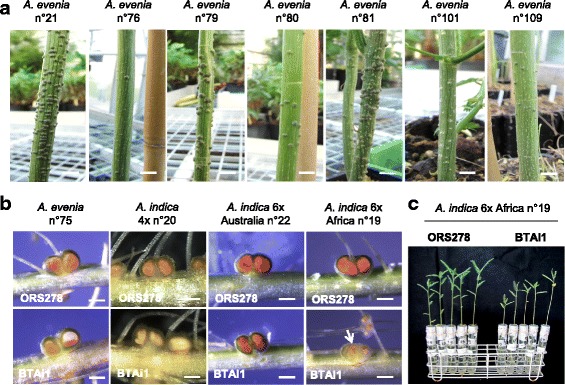
Variation of nodulation traits observed in accessions of *A. evenia* and *A. indica.* (**a**) Stem nodulation observed in various accessions of *A. evenia*, 3 weeks post inoculation with ORS278. (**b**) Root nodule development in accessions of *A. evenia* and *A. indica* following inoculation with *Bradyrhizobium* ORS278 and BTAi1. 14-dpi nodules were cut to observe the leghemoglobin color and reaction defense (arrow). (**c**) Comparison of plant growth (aerial part) after inoculation with *Bradyrhizobium* ORS278 and BTAi1, at 14 dpi. Scale bar in (a): 5 mm, in (b): 1 mm

To test whether this genetic diversity could support polymorphism in nodulation traits, a number of accessions of *A. evenia* s.s. and *A. indica* were submitted to root or stem inoculation with two photosynthetic *nodABC* gene-lacking *Bradyrhizobium* strains, ORS278 and BTAi1. A high variation in stem nodule development was observed in *A. evenia* when it was inoculated with ORS278 (Fig. 6a). Conversely, root nodulation was more homogenous but plant defense reactions characterized by the accumulation of brown compounds, most probably of polyphenol nature as already described for incompatible interactions [26], were obvious in nodules of *A. indica* 6× -Africa- inoculated with BTAi1 (Fig. 6b, Additional file 14: Figure S4a). This incompatibility was accompanied by a low nitrogen-fixing activity and an overall reduction in plant development (Fig. 6c, Additional file 14: Figure S4b).

## Discussion

This in-depth characterization of a Nod-independent *Aeschynomene* germplasm collection identified four main lineages and uncovered genetic diversity and structure at different scales: cytotypes, species and genotypes (as summarised in Table 1). Interestingly, this group of *Aeschynomene* is composed of mainly diploid taxa (13 out of 19), including the model legume *A. evenia*, which are expected to be the easiest to handle at a genetic standpoint. For the polyploid taxa (4 tetraploids and 2 hexaploids), low-copy nuclear genes and SSR analysis supported an allopolyploid origin and several genome donors could be identified [17; this study]. This must facilitate the identification and distinction of the different subgenomic components of the polyploid genomes and this indicated that these polyploid taxa must behave genetically as diploids. Segregation of molecular markers, however, has not been performed to date at a genome-wide scale and we therefore cannot exclude that they might behave as segmental allopolyploids just as recently reported for peanut and chrysanthemum [27, 28]. Such dual structure may be found notably in *A. pratensis* for which the two expected homeologous versions of the tested low-copy nuclear genes were not always detected.

In addition to shedding light on the genetic basis of the different *Aeschynomene* species, our analyses were used to identify different taxa and to delimit their taxonomic boundaries. Indeed, the approach of genotyping a germplasm collection already has shown to be a powerful tool resolving taxonomic issues and providing the basis of a good taxonomic classification in the legume genus *Lens* that includes the cultivated lentil [29]. Here, the rare species *Aeschynomene pluriarticulata* and *A. magna* were found to have *ITS* and low-copy nuclear gene sequences very similar to those of *A. evenia* and *A. denticulata*, respectively (Fig. 1). The NJ SSR trees further revealed that these two pairs of taxa clustered closely together. This strongly suggested that *A. pluriarticulata* and *A. magna* are more likely morphological variants of *A. evenia* and *A. denticulata* than distinct species (Fig. 3). But for *A. magna*, living material is now necessary to confirm this. Conversely, *A. evenia* ssp. *evenia* and *A. evenia* ssp. *serrulata* were found to form sister clades in the *ITS* and low-copy nuclear gene phylogenies (in accordance with their current grouping into a single species) but are clearly distinct entities in the SSR tree (Figs. 1,3). Concordant with this strong genetic differentiation, the two subspecies of *A. evenia* previously were demonstrated not to be cross-compatible [18]. Similarly, *A. virginica* and *A.* sp. 353, along with the African and Australian 6× *A. indica* taxa, formed separate groups in the SSR tree and their hybridization generated F1 hybrids with a marked reduction in fertility (Figs. 2,3). This raises the question of whether these taxa should be treated at the subspecies level or as separate species. On the other hand, the situation was clear for *Aeschynomene* sp. (328); this taxon was consistently found to be divergent in the *ITS*, low-copy nuclear gene and the SSR trees (Figs. 1,3). Therefore, it can be reliably considered as a new species belonging to the *A. sensitiva* group, notwithstanding that this conclusion is based on a single accession of what is apparently a rare taxon.

Given their high variability, the SSR markers are a powerful tool to highlight putative subdivisions in different *Aeschynomene* species that served to define genotypes. Striking is that these genotypes, delineated solely on the basis of marker data, corresponded to geographically based subgroups. Thus, the genetic structure of the *Aeschynomene* taxa appeared to mirror the eco-geographic distribution of the associated genotypes, a situation also described for other plants including lentil, tomato, pigeonpea and switchgrass [30–33]. Although not a major aim of this study, such genetic structure can help to understand the origin and the migration of some populations. This is notably the case for *A. indica* whose natural distribution range is obscure. The identified genetic clusters, which most prominently corresponded with geographical distribution patterns, likely reflected real differences within each species. Among the species studied, *A. evenia* s.s. had the highest genetic diversity with 7 genotypes, some of them previously being shown to be fully cross-compatible [18]. It is also noteworthy that the different *Aeschynomene* taxa displayed very high selfing rates*.* Using lines that tend to be mostly homozygous facilitates artificial hybridizations and analysis of confidently segregation patterns in the progeny in order to investigate the genetic determinism underlying the nodulation traits.

All these data taken together make our germplasm collection a valuable genetic resource for the Nod-independent *Aeschynomene* group. So how best to further exploit it? To decipher the molecular mechanisms underlying the different original nodulation properties found in the Nod-independent symbiosis, *A. evenia* was recently selected as a model species [16, 18]. The species is currently being subjected to full genome sequence analysis and an ongoing mutagenesis project is predicted to identify new symbiotic genetic determinants in the near future. Such approaches applied to the historical model legumes *M. truncatula* and *L. japonicus* led to major advances in the study of the nitrogen-fixing symbiosis with the identification of a set of symbiotic genes involved in the recognition of rhizobial signals, transduction, infection and nodule organogenesis [2, 4]. But mutants are usually screened for the loss of their ability to establish a symbiosis, due to the disruption of gene function, and they are developed in the frame of the study of a single plant line-rhizobial strain system. Conversely, the screening of natural populations with multiple rhizobial strains can reveal some symbiotic phenotypes that depend on both the host genetic background and the rhizobial strain. In fact, studying natural variation approach has been shown to be a powerful tool for gaining insights into the genetic basis underlying the specificity of the symbiotic interaction in three legumes, Medicago, Lotus or soybean. The identified symbiotic polymorphisms were mostly of two types: some plant-rhizobial strain combinations resulted in non-nodulating phenotypes (Nod^−^), others in the production of small white infected nodules proved to be defective in nitrogen fixation (Fix^−^) [34–38]. This indicated that the control of host-rhizobial strain compatibility occurred at two different levels in the symbiotic process.

It is noteworthy that the genetic analysis of natural variation shed light on the function of some key symbiotic genes. The LysM-RLK receptors, which were identified using the mutant approach to be the probable Nod factor receptors, represent a well-known example. Indeed, a synteny-based positional cloning identified *LYK3* in Medicago as corresponding to the *SYM2* gene that controls the symbiotic infection in a Nod factor structure dependent manner in the pea ‘Afghanistan’ ecotype [34, 38]. The diversity information has also been exploited in Lotus to further substantiate that the LysM-RLK receptors mediate specific recognition of Nod factors [39]. More recently, our current understanding of the symbiotic mechanisms has been challenged for the supposed role of NCR peptides that were initially shown as important effectors of endosymbiont’s differentiation to nitrogen-fixing bacteroids. Making use of the differential ability of *Sinorhizobium meliloti* Rm41 to form functional or non-nitrogen fixing nodules depending on the Medicago accession used, two genes, *NFS1* and *NFS2*, were identified and shown to code for NCR peptides [36–40]. This broadened the role of the NCR peptides in the fixation stage by revealing that some of them also control discrimination against incompatible microsymbionts. A thorough survey of naturally occurring variation also provided ground for the discovery of new genetic determinants of nodulation. This was notably the case in soybean where this approach led to the identification of two dominant genes that restrict nodulation in a strain-specific manner, *Rj2/Rfg1* encoding a TIR-NBS-LRR resistance (*R*) gene and *Rj4* that codes for a thaumatin-like protein [41, 42]. This revealed that some host genotypes are able to trigger gene-for-gene resistance, which is found in plant-pathogen interactions, to selectively interact with certain symbiotic strains but to exclude others.

These illuminating examples show that there is a mileage to be gained from exploring the natural variation in nodulation in the Nod-independent *Aeschynomene* group. To screen our germplasm collection, many *Bradyrhizobium* strains are available, including photosynthetic and non-photosynthetic ones [14, 43, 44]. A recent report revealed important variations in the ability of different Nod-independent *Aeschynomene* species to be nodulated in a T3SS-dependent fashion by the non-photosynthetic *Bradyrhizobium* strains STM6978 and USDA61 [14]. Such marked variations were also observed between different accessions of *A. evenia* s.s., providing the basis for a genetic analysis of this differential susceptibility. Variations in symbiotic traits with naturally-nodulating photosynthetic *Bradyrhizobium* strains have not been thoroughly surveyed yet, but the observations made in the present study using the strains ORS278 and BTAi1 yield promising preliminary results. By evaluating different genotype-rhizobial combinations, we predict that symbiotic polymorphisms will be found, notably in the induction of nodulation, nitrogen fixation and also in stem nodulation. Crossable accessions exhibiting polymorphic symbiotic phenotypes can then be selected for hybridization experiments, subsequent genetic analysis and locus mapping. This will pave the way for the identification of the genes underpinning these symbiotic responses through forward or reverse genetics. To assist in these studies, genomic and genetic data, together with a number of molecular tools, are being accumulated for the model species *A. evenia* and it thus represents the easiest system to work with [16, 20]. Despite the high genetic diversity observed in *A. evenia*, the number of available germplasm samples remains relatively modest since it has not been as extensively sampled as other legumes of interest [22]. Therefore, the accessions of other Nod-independent taxa in the same gene pool or in the related one containing *A. sensitiva*, represent a good complement. In addition, the expected high level of microsynteny with *A. evenia* will facilitate a synteny-based positional approach, as has been successfully performed between Pea and Medicago to identify the gene underlying the *SYM2* locus [34, 45]. As a result, genetic resources developed for the Nod-independent *Aeschynomene* clade can be fully exploited for the search of natural variation in nodulation with *Bradyrhizobium*.

## Conclusions

With the goal of discovering natural variation in the Nod-independent *Aeschynomene* legumes, we developed a large collection of 226 accessions that spans the geographical distribution of the different taxa in this group, including the model species *A. evenia*. These accessions were subjected to combined analyses of gene sequencing, cytogenetics, hybridization experiments and SSR genotyping. This work resulted in the delineation of taxon boundaries and in the discovery of new genotypes. Taxa displaying a significant genetic diversity were characterized by a clear geographically-based genetic structure. In addition, low-copy nuclear genes and patterns of microsatellite diversity illuminated the genetic basis of the *Aeschynomene* diploid and polyploid taxa that are all predominantly autogamous and have a predicted simple disomic inheritance, two attributes favorable for genetics. Such a well-characterized collection of accessions constitutes a major genetic resource for exploring the natural variation of nodulation in response to different Bradyrhizobia strains and for searching for their underlying genetic determinants. Discoveries of alternative functions in symbiotic genes identified in other model legumes or of new genes involved in the recognition of the still unknown non-Nod bacterial signal, nodule functioning and in the restriction of compatibility would add a new dimension to our understanding of the genetic control of nodulation in the Nod-independent symbiosis.

## Methods

### Plant material

All the accessions of *Aeschynomene* used in this study, their geographical origin and source data are listed in Additional file 1: Table S1. Seeds were scarified with sulphuric acid for germination and plants were grown in pots filled with compost under greenhouse conditions (temperature: 26-36 °C, relative humidity: 70%-80%, insect-proof screens) as detailed [16]. Interspecific crosses were performed according to the protocol developed earlier and the nature of the resulting hybrids was checked using SSR markers (data not shown) [16]. Fertility of the F1 plants was assessed by recording the number of successfully developed pods per flowering axis and the number of seeds in each pod.

### Plant nodulation and ARA

Nodulation tests were carried out using *Bradyrhizobium* sp. strains ORS278 and BTAi1 [12]. The strains were cultivated for 7 days in yeast-mannitol liquid medium at 34 °C. Root inoculation was performed in an in vitro growth chamber on 7-day-old plants using 1 mL of bacterial culture with an optical density at 600 nm adjusted to 1. Stem inoculation was carried out in a greenhouse by wrapping the stem of 5-week-old plants with a paper soaked with bacterial culture for 24 h. Stem nodules were observed at 21dpi and root nodules at 14dpi. Nitrogen-fixing activity was estimated on the entire plant by measurement of acetylene reducing activity (ARA) and microscopic observations were performed using a stereo-microscope (Nikon AZ100, Champigny-sur-Marne, France) as previously published [46].

### Genome size estimation and chromosome counting

Flow cytometry measurement was performed on leaf material to estimate genome sizes of various accessions as already explained [16]. These estimates were based on the measurements of three plants per accession using *Lycopersicum esculentum* (Solanaceae) cv “Roma” (2C = 1.99 pg) as the internal standard. For chromosome number counts, metaphasic chromosomes were prepared from root-tips, spread on slides and stained with DAPI (4′,6-diamidino-2-phenylindole). Chromosomes were counted from images obtained with a fluorescent microscope as previously described [16].

### Plant DNA extraction

Genomic DNA was extracted from fresh leaves using the CTAB (Cetyl Trimethyl Ammonium Bromide) method improved by the addition of β-mercaptoethanol 2% and PVPP 2%. For herbarium material, a protocol adapted for fragmented DNA was used, with increased length of the incubation (90 min), centrifugation (20 min) and precipitation (15 min) steps was used. DNA quantity was evaluated by spectrophotometer and DNA samples normalized to a uniform concentration of 10 ng/μL.

### Gene sequencing and sequence analysis

The nuclear ribosomal internal transcribed spacer region (ITS: ITS1-5.8S rDNA gene-ITS2) and five low copy nuclear genes *CYP1*, *eiF1α*, Sucrose Synthase, *SUI1* and a gene homolog to Glyma.07G136800 and Glyma.18G187300 identified in *Glycine max* were amplified with the primers listed in Additional file 2: Table S2. PCR amplifications, cloning and sequencing of PCR products were performed as already described [17, 18]. The DNA sequences generated in this study were deposited in Genbank (Additional file 3: Table S3) and additional sequences are available in Additional file 4: Doc. S1. For the phylogenetic analyses, the gene sequences were aligned in ClustalX, version 1.81b and the alignments were checked in Genedoc v2.7. Phylogenetic reconstructions were performed with the MEGA v7 program using the Neighbor Joining approach and the Tamura 3-parameter model with a 1000 x bootstrap.

### SSR marker selection and genotyping

A total of 500 primer pairs were initially defined to develop SSR markers for genetic mapping in *A. evenia* (Additional file 7: Table S4) [9]. In the present study, they were again tested on two polymorphic accessions of *A. evenia* and two cytotypes of *A. indica* (Additional file 8: Table S5). Forward primers all contained a 5′-end M13 tail (5′-CACGACGTTGTAAAACGAC-3′), enabling the tagging the PCR products during the PCR amplification with 4 M13 primer-fluorescent dyes 6-FAM™, NED®, VIC®, or PET® (Applied Biosystems, CA, USA). Amplicon sizes were analysed using an ABI 3700 automatic capillary sequencer (Applied Biosystems) as previously described [9]. 54 SSR markers were organized in 4-SSR multiplexes and used to genotype the accessions belonging to the *A. evenia* and the *A. sensitiva* groups as detailed in Additional file 6: Figure S2. Allele scorings were analysed using GeneMapper 4.0 software (Applied Biosystems) and exported as data tables for two groups of genotyping.

### SSR data analysis

Genotyping data files were assembled in a single database that was used to determine genetic relationships among the accessions. For this, a distance-based approach was applied by calculating with a shared allele index a genetic dissimilarity matrix in DARwin v5 software [25]. Then, individual relations were separately analyzed for each ploidy level (2×, 4×, 6×) with a tree construction based on an unweighted Neighbor Joining method while genetic affinities between diploid and polyploid species were investigated by a factorial analysis as implemented in DARwin v5. The genetic diversity was evaluated by computing the number of alleles per locus (*Na*) and the observed heterozygosities *Ho* for each SSR locus and for different accession groups (Additional files 12 and 13: Tables S8, S9).

## Additional files

Additional file 1: **Table S1.** Accessions used in this study, origin and characteristics. (XLSX 23 kb)

Additional file 2: **Table S2.** Nuclear genes used for the phylogenetic analyses. (XLSX 11 kb)

Additional file 3: **Table S3.** GenBank numbers for the sequences used in the phylogenetic analyses. (XLSX 12 kb)

Additional file 4: **Doc. S1.** *ITS* sequences obtained for the Nod-independent *Aeschynomene* accessions. (DOCX 30 kb)

Additional file 5: **Figure S1.** Chromosome numbers in new *Aeschynomene* taxa. Root tip metaphase chromosomes stained in blue with DAPI (4′,6-diamidino-2-phenylindole). Chromosome numbers are indicated in brackets. Scale bars: 5 μm. (PPTX 1008 kb)

Additional file 6: **Figure S2.** Schematic representation of the different steps of the genotyping process from marker selection to data treatment. (PPTX 64 kb)

Additional file 7: **Table S4.** Origin, location and primer sequences for the SSR markers used for genotyping. (XLSX 13 kb)

Additional file 8: **Table S5.** Repeat motif and allelic amplification profiles of the SSR selected for genotyping. (XLSX 13 kb)

Additional file 9: **Figure S3.** Detailed NJ trees representing the genetic diversity among the Nod-independent *Aeschynomene* accessions. The trees were developed separately in DARWIN using the allelic data of 65 SSRs for the 2× (a), 4× (b) and 6× (c) taxa. Well-differentiated taxa are distinctly colored. Identified genotypes are marked with a red dot and numbered. Accessions are designated with their LSTM code mentioned in Additional file 1: Table S1 followed by their geographical origin. Species suspected to be morphological variants are marked with an asterisk. Taxon colours and genotype numbers are the same as in Fig. 3. (PPTX 187 kb)

Additional file 10: **Table S6.** Allelic diversity of the SSR markers (XLSX 22 kb)

Additional file 11: **Table S7.** Cross-species transferability of the SSR markers (XLSX 13 kb)

Additional file 12: **Table S8.** Scoring of mean allele number per SSR and species (XLSX 15 kb)

Additional file 13: **Table S9.** Observed heterozygosity (*Ho*) in *Aeschynomene* species (XLSX 14 kb)

Additional file 14: **Figure S4.** Comparison of the nodulation properties of *A. evenia* s.s. and *A. indica*. Different accessions were root inoculated with Bradyrhizobium ORS278 and BTAi1 and analysed at 14dpi. (a) Number of nodules per accession. (b) Acetylene-reducing activity (ARA). A.U. Arbitrary Unit. Error bars represent s.d. (*n* = 6). (PPTX 179 kb)
