# Supplementary material for: Naturally occurring variations in the nod-independent model legume Aeschynomene evenia and relatives: a resource for nodulation genetics
Source: BMC Plant Biol. 2018 Apr 3;18:54. doi: 10.1186/s12870-018-1260-2 (PMC5883870; doi:10.1186/s12870-018-1260-2)
Supplement: Supplementary file 4 — Doc. S1. ITS sequences obtained for the Nod-independent Aeschynomene accessions. (DOCX 30 kb) [file 12870_2018_1260_MOESM4_ESM.docx]

>Aciliata-48

GTAACAAGGTTTCCGTAGGTGAACCTGCGGAAGGATCATTGTTGATGCCTCGACCCAGCTAGACCTGCGAATGCGTTTTACTACCCGGGGTGATCGGGCTGCCTAGGCAGCTCGCCTCCCCGAACCCGTTGGGGCTGTGGCCATCCTTTGTGGCCCGGTCTCGACACAACAACAAACCCCGGCGCGGAATGCGCCAAGGAATCACAATCACAAGGCGCGCCCCCTCGACCCGGAAGCGGTGTTCGTCTGGGTGGTGTCGCAAAAAATTGAGTCCAAAATGACTCTCGGCAACGGATATCTCGGCTCTTGCATCGATGAAGAACGTAGCGAAATGCGATACTTGGTGTGAATTGCAGAATCCCGTGAACCATCGAGTCTTTGAACGCAAGTTGCGCCCGAAGCCATTAGGCTAAGGGCACGCCTGCCTGGGTGTCACCAATCGTCGCCCCCAACCTCACTGCCTTGTTGCGTGGAGAAGGGGTGAATGATGGCTTCCCGTGAGCACCGTCTCGCGGTTGGCTGAAAACATTCTCCGTGCCGGCGTGCAGCGCCGTGACACTTGGTGGTTGAGTTTACTCTCGAGGCCAGTCACGTGTGCTCCCTGTCGGTTCCGGAAGCATGGACCCGTGAGCGGCAAAGACCGCCCTTGATGCGACCTCAGGTCAGGCGGGGCTACCCGCTGAGTTTAAGCATATCAATAAGCG

>Aciliata_235

GTAACAAGGTTTCCGTAGGTGAaCCTGCGGAAGGaTCATTGTTGATGCCTCGACCCAGCTAGACCCGCGAATGCGTTTTACTACCCGGGGTGATCGGGCTGCCTAGGCAGCTCGCCTCCCCGACCCGTTGGGGCTGTGGCCATCCTTTGTGGCCCGGTCTCGACACAACAACAAACCCCGGCGCGGAATGCGCCAAGGAATCACAATCACAAGGCGCGCCCCCTCgACCCGGAaGCGGTGTTCGTCTGGGTGGCGTCGCAAAAAATCGAGTCCAAAATGACTCTCGGCAACGGATATCTCGGCTCTtGCATCGATgAAgAACGTAGCGAAaTGCGATACTTGGTGTGAATTGCAgAATCCCGTGAACCATCGAGTCTTTGAACGCAaGTTGCGCCCGAAGCCATTAGGCTAAGGGCACGCCtGCCTGGGTGTCACCAATCGTCGCCCCCAACCTCACTGCCTTGTTGCGTGGAGAAGGGGTGAATGATGGCTTCCCGTGAGCACCGTCTCGCGgTTGGCTGAAAACATTCTCCGTGCCGGCGTGCAGCGCCgTGACACTtGGTGGTTGAGTTTACTCTCGAGGCCAGTCACGTGTGCTCCCTGTCGGTTCCgGAAGCATGGACCCGTGAGCGGCAAAGACCGCCCTTGATGCgACCTCagGTCAggCGGGGCTACCCGCTGAGTTTAAGCATATCAaTAAGCG

>Aciliata_272-A

GTAACAAGGTTTCCGTAGGTGAaCCTGCGGAAGGaTCATTGTTGATGCCTCGACCCAGCTAGACCCGCGAATGCGTTTTACTACCCGGGGTGATCGGGCTGCCTAGGCAGCTCGCCTCCCCGACCCGTTGGGGCTGTGGCCATCCTTTGTGGCCCGGTCTCGACACAACAACAAACCCCGGCGCGGAATGCGCCAAGGAATCACAATCACAAGGCGCGCCCCCTCgACCCGGAaGCGGTGTTCGTCTGGGTGGCGTCGCAAAAAATCGAGTCCAAAATGACTCTCGGCAACGGATATCTCGGCTCTtGCATCGATgAAgAACGTAGCGAAaTGCGATACTTGGTGTGAATTGCAgAATCCCGTGAACCATCGAGTCTTTGAACGCAaGTTGCGCCCGAAGCCATTAGGCTAAGGGCACGCCtGCCTGGGTGTCACCAATCGTCGCCCCCAACCTCACTGCCTTGTTGCGTGGAGAAGGGGTGAATGATGGCTTCCCGTGAGCACCGTCTCGCGgTTGGCTGAAAACATTCTCCGTGCCGGCGTGCAGCGCCgTGACACTtGGTGGTTGAGTTTACTCTCGAGGCCAGTCACGTGTGCTCCCTGTCGGTTCCgGAAGCATGGACCCGTGAGCGGCAAAGACCGCCCTTGATGCgACCTCagGTCAggCGGGGCTACCCGCTGAGTTTAAGCATATCAaTAAGCG

>Aciliata_272-B

GTAACAAgGtTTCCgTAGGTGAACCTGCGGAAGGaTCATTGTTGATGCCTCGACCCAGCTAGACCCGCGAATGCGTTTAACTACCCGGGGTGATCGGGCTGCCTAGGCAGCTCGCCTCCCCGACCCGTTGGGGCTGTGGCCATCCTTTGTGGCCCGGTCTCGACACAACAACAAACCCCGGCGCGGAATGCGCCAAGGAATCACAATCACAAGGCGCCCCCTCGACCCGGAAGCGGTGTTCGTCTGGGTGGCGTCGCAAAAAATCGAGTCCAAAATGACTCTCGGCAACGGATATCTCGGCTCTTGCATCGATGAAGAACGTAGCGAAATGCGATACTTGGTGTGAATTGCAGAATCCCGTGAACCATCGAGTCTTTGAACGCAAGTTGCGCCCGAAGCCATTAGGCTAAGGGCACGCCTGCCTGGGTGTCACCAATCGTCACCCCCAACCTCACTGCCTTGTTGCGTGGAGAAGGGGTGAATGATGGCTTCCCGTGAGCACCGTCTCGCGGTTGGCTGAAAACATTCTCCGTGCCGGCGTGCAGCGCCGTGACACTTGGTGGTTGAGTTTACTCTCGAGGCCAGTCACGTGTGCTCCCTGTCGGTTCCGGAAGCATGGACCCGTGAGCGGCAAAGACCGCCCTTGATGCGACCTCAGGTCAGGCGGGGCTACCCGCtGAGTTTAAGCATATCAaTAAGCG

>Aciliata-330

GTAACAAgGtTTCCGTAGGTGAACCTGCGGAAGGatCATTGTTGATGCCTCGACCCAGCTAGACCCGCGAATGCGTTTTACTACCCGGGGTGATCGGGCTGCCTAGGCAGCTCGCCTCCCCGACCCGTTGGGGCTGTGGCCATCCTTTGTGGCCCGGTCTCGACACAACAACAAACCCCGGCGCGGAATGCGCCAAGGAATCACAATCACAAGGCGCGCCCCCTCGACCCGGAAGCGGTGTTCGTCTGGGTGGCGTCGCAAAAAATCGAGTCCAAAATGACTCTCGGCAACGGATATCTCGGCTCTTGCATCGATGAAGAACGTAGCGAAATGCGATACTTGGTGTGAATTGCAGAATCCCGTGAACCATCGAGTCTTTGAACGCAAGTTGCGCCCGAAGCCATTAGGCTAAGGGCACGCCTGCCTGGGTGTCACCAATCGTCGCCCCCAACCTCACTGCCTTGTTGCGTGGAGAAGGGGTGAATGATGGCTTCCCGTGAGCACCGTCTCGCGGTTGGCTGAAAACATTCTCCGTGCCGGCGTGCAGCGCCgTGACACTTGGTGGTTGAGTTTACTCTCGAGGCCAGTCACGTGTGCTCCCTGTCGGTTCCGGAAGCATGGACCCGTGAGCGGCAAAGACCGCCCTTGATGCGACCTCAGGTCAGGCGGGGCTACCCGCTGAGTTTAAGCATATCAaTAAGCG

>Aciliata-346

GTAACAAgGtTTCCgTAGGTGAACCTGCGGAAGGaTCATTGTTGATGCCTCGACCCAGCTAGACCCGCGAATGCGTTTAACTACCCGGGGTGATCGGGCTGCCTAGGCAGCTCGCCTCCCCGACCCGTTGGGGCTGTGGCCATCCTTTGTGGCCCGGTCTCGACACAACAACAAACCCCGGCGCGGAATGCGCCAAGGAATCACAATCACAAGGCGCCCCCTCGACCCGGAAGCGGTGTTCGTCTGGGTGGCGTCGCAAAAAATCGAGTCCAAAATGACTCTCGGCAACGGATATCTCGGCTCTTGCATCGATGAAGAACGTAGCGAAATGCGATACTTGGTGTGAATTGCAGAATCCCGTGAACCATCGAGTCTTTGAACGCAAGTTGCGCCCGAAGCCATTAGGCTAAGGGCACGCCTGCCTGGGTGTCACCAATCGTCACCCCCAACCTCACTGCCTTGTTGCGTGGAGAAGGGGTGAATGATGGCTTCCCGTGAGCACCGTCTCGCGGTTGGCTGAAAACATTCTCCGTGCCGGCGTGCAGCGCCGTGACACTTGGTGGTTGAGTTTACTCTCGAGGCCAGTCACGTGTGCTCCCTGTCGGTTCCGGAAGCATGGACCCGTGAGCGGCAAAGACCGCCCTTGATGCGACCTCAGGTCAGGCGGGGCTACCCGCtGAGTTTAAGCATATCAaTAAGCG

>Adeamii-24

GTAACAAGGTTTCCGTAGGTGAACCTGCGGAAGGATCATTGTTGATGCCTCAATCCGGACAGACCCGCGAATGCGTTTCACCGCCCGGGGCGGTCGAGCTGCCCGGGCAGCTCGCCTCCCCGGACCGTCGGGGCGTGGCCACCCTGTGCGGCCAGGTCCCGGCACAACAACAAACCCCGGCGCGGAATGCGCCAAGGAATTCACAATCGTCAGACGCTCCGGCTCGGCCCGGCAACGGTGCCCGTCCGGGTGGCGTCGTGATAATCGAGTCCAAAATGACTCTCGGCAACGGATATCTCGGCTCTTGCATCGATGAAGAACGTAGCGAAATGCGATACTTGGTGTGAATTGCAGAATCCCGTGAACCATCGAGTCTTTGAACGCAAGTTGCGCCCGAAGCCATTAGGCCAAGGGCACGCCTGCCTGGGTGTCACCAATCGCCGCCCCAACCCCTGCGCCTCCGGGCGCGGAGCAGGGTGAAAGCTGGCTTCCCGTGAGCGCTGCCTCGCGGTTGGCCGAAAACATTCTCCGTACCGGCGCGCGGCGCCGTGACGCTTGGTGGTAGAGTTTGTTCTCGAGGCCAGTCACGGGCGCCCCCGGTCGGCTGCGGAAACAGTGACCCGTGCGCGGCTCTGACCGTCCATGAAGCGACCTCAGGTCAGGCGGGGCCACCCGCTGAGTTTAAGCATATCAATAAGCG

>Adeamii-259

GTAACAAGGTTTCCGTAGGTGAACCTGCGGAAGGATCATTGTTGATGCCTCAATCCGGACAGACCCGCGAATGCGTTTCACCGCCCGGGGCGGTCGAGCTGCCCGGGCAGCTCGCCTCCCCGGACCGTCGGGGCGTGGCCACCCTGTGCGGCCAGGTCCCGGCACAACAACAAACCCCGGCGCGGAATGCGCCAAGGAATTCACAATCGTCAGACGCTCCGGCTCGGCCCGGCAACGGTGCCCGTCCGGGTGGCGTCGTGATAATCGAGTCCAAAATGACTCTCGGCAACGGATATCTCGGCTCTTGCATCGATGAAGAACGTAGCGAAATGCGATACTTGGTGTGAATTGCAGAATCCCGTGAACCATCGAGTCTTTGAACGCAAGTTGCGCCCGAAGCCATTAGGCCAAGGGCACGCCTGCCTGGGTGTCACCAATCGCCGCCCCAACCCCTGCGCCTCCGGGCGCGGAGCAGGGTGAAAGCTGGCTTCCCGTGAGCGCTGCCTCGCGGTTGGCCGAAAACATTCTCCGTACCGGCGCGCGGCGCCGTGACGCTTGGTGGTAGAGTTTGTTCTCGAGGCCAGTCACGGGCGCCCCCGGTCGGCTGCGGAAACAGTGACCCGTGCGCGGCTCTGACCGTCCATGAAGCGACCTCAGGTCAGGCGGGGCCACCCGCTGAGTTTAAGCATATCAATAAGCG

>Adeamii-340

GTAACAAGGTTTCCGTAGGTGAACCTGCGGAAGGATCATTGTTGATGCCTCAATCCGGACAGACCCGCGAATGCGTTTCACCGCCCGGGGCGGTCGAGCTGCCCGGGCAGCTCGCCTCCCCGGACCGTCGGGGCGTGGCCACCCTGTGCGGCCAGGTCCCGGCACAACAACAAACCCCGGCGCGGAATGCGCCAAGGAATTCACAATCGTCAGACGCTCCGGCTCGGCCCGGCAACGGTGCCCGTCCGGGTGGCGTCGTGATAATCGAGTCCAAAATGACTCTCGGCAACGGATATCTCGGCTCTTGCATCGATGAAGAACGTAGCGAAATGCGATACTTGGTGTGAATTGCAGAATCCCGTGAACCATCGAGTCTTTGAACGCAAGTTGCGCCCGAAGCCATTAGGCCAAGGGCACGCCTGCCTGGGTGTCACCAATCGCCGCCCCAACCCCTGCGCCTCCGGGCGCGGAGCAGGGTGAAAGCTGGCTTCCCGTGAGCGCTGCCTCGCGGTTGGCCGAAAACATTCTCCGTACCGGCGCGCGGCGCCGTGACGCTTGGTGGTAGAGTTTGTTCTCGAGGCCAGTCACGGGCGCCCCCGGTCGGCTGCGGAAACAGTGACCCGTGCGCGGCTCTGACCGTCCATGAAGCGACCTCAGGTCAGGCGGGGCCACCCGCTGAGTTTAAGCATATCAATAAGCG

>Adenticulata-12

GTAACAAGGTTTCCGTAGGTGAACCTGCGGAAG

GATCATTGTTGATGCCTCGACCCAGCTAGACCCGTGAATGCGTTTTACTACCCGGGGTGATCGGGCTGCCTAGGCAGCTCGCCTCCCTGACTCGTTGGGGCTCTGGCCGCCCTGTGTGGCCCGGTCCCGACACAACAACAAACCCCGGCGCGGAATGCGCCAAGGAATAACAATCACAAGGCGTGCCCCCTCGACCCGGAAGCGGTGTTCGTATGGGTGGCGTCGCAAAAAATTGAGTCCAAAATGACTCTCGGCAACGGATATCTCGGCTCTTGCATCGATGAAGAACGTAGCGAAATGCGATACTTGGTGTGAATTGCAGAATCCCGTGAACCATCGAGTCTTTGAACGCAAGTTGCGCCCGAAGCCATTAGGCTAAGGGCACGCCTGCCTGGGTGTCACCAATCGTCGCCCCCAACCTCACTGCCTCGTTGCGTGGGGAAGGGGTGAATGATGGCTTCCCGTGAGCACGGTCTCGCGGTTGGCTGAAAACGTTCTCCGTGCTGGCGTGCAGCGCCGTGACACTTGGTGGTTGAGTTTACCCTCGAGGCCAGTCACGTGTGCTCCCTGTCGGTTCCGGAAGCATGGACCCGTGAGCGGCAAAGACCGCTCTTGATGCGACCTCAGGTCAGGCGGGGCTACCCGCTGAGTTTAAGCATATCAATAAGCG

>Adenticulata-50

GTAACAAGGTTTCCGTAGGTGAACCTGCGGAAGGATCATTGTTGATGCCTCGACCCAGCTAGACCCGTGAATGCGTTTTACTACCCGGGGTGATCGGGCTGCCTAGGCAGCTCGCCTCCCTGACTCGTTGGGGCTCTGGCCGCCCTGTGTGGCCCGGTCCCGACACAACAACAAACCCCGGCGCGGAATGCGCCAAGGAATAACAATCACAAGGCGTGCCCCCTCGACCCGGAAGCGGTGTTCGTATGGGTGGCGTCGCAAAAAATTGAGTCCAAAATGACTCTCGGCAACGGATATCTCGGCTCTTGCATCGATGAAGAACGTAGCGAAATGCGATACTTGGTGTGAATTGCAGAATCCCGTGAACCATCGAGTCTTTGAACGCAAGTTGCGCCCGAAGCCATTAGGCTAAGGGCACGCCTGCCTGGGTGTCACCAATCGTCGCCCCCAACCTCACTGCCTCGTTGCGTGGGGAAGGGGTGAATGATGGCTTCCCGTGAGCACGGTCTCGCGGTTGGCTGAAAACGTTCTCCGTGCTGGCGTGCAGCGCCGTGACACTTGGTGGTTGAGTTTACCCTCGAGGCCAGTCACGTGTGCTCCCTGTCGGTTCCGGAAGCATGGACCCGTGAGCGGCAAAGACCGCTCTTGATGCGACCTCAGGTCAGGCGGGGCTACCCGCTGAGTTTAAGCATATCAATAAGCG

>Adenticulata-91

GTAACAAGGTTTCCGTAGGTGAACCTGCGGAAGGATCATTGTTGATGCCTCGACCCAGCTAGACCCGCGAATGCGTTTTACTACCCGGGGTGATCGGGCTGCCTAGGCAGCTCGCCTCCCTGACTCGTTGGGGCTCTGGCCGCCCTGTGTGGCCCGGTCCCGACACAACAACAAACCCCGGCGCGGAATGCGCCAAGGAATAACAATCACAAGGCGTGCCCCCTCGACCCGGAAGCGGTGTTCGTATGGGTGGCGTCGCAAAAAATTGAGTCCAAAATGACTCTCGGCAACGGATATCTCGGCTCTTGCATCGATGAAGAACGTAGCGAAATGCGATACTTGGTGTGAATTGCAGAATCCCGTGAACCATCGAGTCTTTGAACGCAAGTTGCGCCCGAAGCCATTAGGCTAAGGGCACGCCTGCCTGGGTGTCACCAATCGTCGCCCCCAACCTCACTGCCTCGTTGCGTGGGGAAGGGGTGAATGATGGCTTCCCGTGAGCACGGTCTCGCGGTTGGCTGAAAACGTTCTCCGTGCTGGCGTGCAGCGCCGTGACACTTGGTGGTTGAGTTTACCCTCGAGGCCAGTCACGTGTGCTCCCTGTCGGTTCCGGAAGCATGGACCCGTGAGCGGCAAAGACCGCCCTTGATGCGACCTCAGGTCAGGCGGGGCTACCCGCTGAGTTTAAGCATATCAATAAGCG

>Adenticulata-199

GTAACAAGGTTTCCGTAGGTGAACCTGCGGAAGGATCATTGTTGATGCCTCGACCCAGCTAGACCCGCGAATGCGTTTTACTACCCGGGGTGATCGGGCTGCCTAGGCAGCTCGCCTCCCTGACTCGTTGGGGCTCTGGCCGCCCTGTGTGGCCCGGTCCCGACACAACAACAAACCCCGGCGCGGAATGCGCCAAGGAATAACAATCACAAGGCGTGCCCCCTCGACCCGGAAGCGGTGTTCGTATGGGTGGCGTCGCAAAAAATTGAGTCCAAAATGACTCTCGGCAACGGATATCTCGGCTCTTGCATCGATGAAGAACGTAGCGAAATGCGATACTTGGTGTGAATTGCAGAATCCCGTGAACCATCGAGTCTTTGAACGCAAGTTGCGCCCGAAGCCATTAGGCTAAGGGCACGCCTGCCTGGGTGTCACCAATCGTCGCCCCCAACCTCACTGCCTCGTTGCGTGGGGAAGGGGTGAATGATGGCTTCCCGTGAGCACGGTCTCGCGGTTGGCTGAAAACGTTCTCCGTGCTGGCGTGCAGCGCCGTGACACTTGGTGGTTGAGTTTACCCTCGAGGCCAGTCACGTGTGCTCCCTGTCGGTTCCGGAAGCATGGACCCGTGAGCGGCAAAGACCGCCCTTGATGCGACCTCAGGTCAGGCGGGGCTACCCGCTGAGTTTAAGCATATCAATAAGCG

>Adenticulata_222

GTAACAAGGTTTCCGTAGGTGAacCTGCGGAAGGAtCATTGTTGATGCCTCGACCCAGCTAGACCCGTGAATGCGTTTTACTACCCGGGGTGATCGGGCTGCCTAGGCAGCTCGCCTCCCTGACTCGTTGGGGCTCTGGCCGCCCTGTGTGGCCCGGTCCCGACACAACAACAAACCCCGGCGCGGAATGCGCCAAGGAATAACAATCACAAGGCGTGCCCCCTCGACCCGGAAGCGGTGTTCGTATGGGTGGCGTCGCAAAAAATTGAGTCCAAAATGACTCTCGGCAACGGATATCTCGGCTCTTGCATCGATGAAGAACGTAGCGAAATGCGATACTTGGTGTGAATTGCAGAATCCCGTGAACCATCGAGTCTTTGAACGCAAGTTGCGCCCGAAGCCATTAGGCTAAGGGCACGCCTGCCTGGGTGTCACCAATCGTCGCCCCCAACCTCACTGCCTCGTTGCGTGGGGAAGGGGTGAATGATGGCTTCCCGTGAGCACGGTCTCGCGGTTGGCTGAAAACGTTCTCCGTGCTGGCGTGCAGCGCCGTGACACTTGGTGGTTGAGTTTACCCTCGAGGCCAGTCACGTGTGCTCCCTGTCGGTTCCGGAAGCATGGACCCGTGAGCGGCAAAGACCGCCCTTGATGCGACCTCAGGTCAGGCGGGGCTACCCGCTGAGTTTAAGCATATCAATAAGCG

>Adenticulata_230

GTAACAAGGTTTCCGTAGGTGaACCTGCGGAAGGATCATTGTTGATGCCTCAACCCAGCTAGACCCGCGAATACGTTTTACTACCCGGGGTGATTGGGCTGCCTAGGCAGCTCGCCTCCCCGACCCGTTGGGGCTCTGGCCGCCCTGTGTGGCTCGGTCCCGACACAACAACAAACCCCGGCGCGGAATGCGCCAAGGAATAACAATCACAAGGCGTGCCCCCTCGACCCGGAAGCGGTGTTCGTCTGGGTGGCGTCGCAAAAAAATGAGTCCAAAATGACTCTCGGCAACGGATATCTCGGCTCTTGCATCGATGAAGAACGTAGCGAAATGCGATACTTGGTGTGAATTGCAGAATCCCGTGAACCATCGAGTCTTTGAACGCAAGTTGCGCCCGAAGCCATTAGGCTAAGGGCACGCCTGCCTGGGTGTCACCAATCGTCGCCCCCAACCTCACTGCCTCGTTGCGTGGGGAAGGGGTGAATGATGGCTTCCCGTGAGCACGGTCTCGCGGTTGGCTGAAAACGTTCTCCGTGCTGGCGTGCAGCGCCGTGACACTTGGTGGTTGAGTTTACCCTCGAGGCCAGTCACGTGTGCTCCCTGTCGGTTCCGGAAGCATGGACCCGTGAGCGGCAAAGACCGCCCTTGATGCGACCTCAGGTCAGGCGGGGCTACCCGCTGAGTTTAAGCATATCAaTAAGCG

>Adenticulata_231

GTAACAAGGTTTCCGTAGGTGAacCTGCGGAAGGatCATTGTTGAtGCCTCGACCCagATaGACCCGCGAATGCGTTTTACTACCCGGGGTGATcGGGCTGCCTAGGCAGCTCGCCTCCCTGACTCGTTGGGGCTCTGGCCGCCCTGTGTGGCCCGGTCCCGACACAACAACAAACCCCGGCGCGGAATGCGCCAAGGAATAACAATCACAAGGCGTGCCCCCTCGACCCGGAAGCGGTGTTCGTATGGGTGGCGTCGCAAAAAATTGAGTCCAAAATGACTCTCGGCAACGGATATCTCGGCTCTTGCATCGATGAAGAACGTAGCGAAATGCGATACTTGGTGTGAATTGCAGAATCCCGTGAACCATCGAGTCTTTGAACGCAAGTTGCGCCCGAAGCCATTAGGCTAAGGGCACGCCTGCCTGGGTGTCACCAATCGTCGCCCCCAACCTCACTGCCTCGTTGCGTGGGGAAGGGGTGAATGATGGCTTCCCGTGAGCACGGTCTCGCGGTTGGCTGAAAACGTTCTCCGTGCTGGCGTGCAGCGCCGTGACACTTGGTGGTTGAGTTTACCCTCGAGGCCAGTCACGTGTGCTCCCTGTCGGTTCCGGAAGCATGGACCCGTGAGCGGCAAAGACCGCCCTTGATGCGACCTCAGGTCAGGCGGGGCTACCCGCTGAGTTTAAGCATATCAaTAAGCG

>Adenticulata_232

GTAACAAGGTTTCCGTAGGTGAacCTGCGGAAGGatCATTGTTGAtGCCTCGACCCAGCTAGACCCGcGAATGCGTTTTACTACCCGGGGTGATCGGGCTGCCTAGGCAGCTCGCCTCCCTGACTCGTTGGGGCTCTGGCCGCCCTGTGTGGCCCGGTCCCGACACAACAACAAACCCCGGCGCGGAATGCGCCAAGGAATAACAATCACAAGGCGTGCCCCCTCGACCCGGAAGCGGTGTTCGTATGGGTGGCGTCGCAAAAAATTGAGTCCAAAATGACTCTCGGCAACGGATATCTCGGCTCTTGCATCGATGAAGAACGTAGCGAAATGCGATACTTGGTGTGAATTGCAGAATCCCGTGAACCATCGAGTCTTTGAACGCAAGTTGCGCCCGAAGCCATTAGGCTAAGGGCACGCCTGCCTGGGTGTCACCAATCGTCGCCCCCAACCTCACTGCCTCGTTGCGTGGGGAAGGGGTGAATGATGGCTTCCCGTGAGCACGGTCTCGCGGTTGGCTGAAAACGTTCTCCGTGCTGGCGTGCAGCGCCGTGACACTTGGTGGTTGAGTTTACCCTCGAGGCCAGTCACGTGTGCTCCCTGTCGGTTCCGGAAGCATGGACCCGTGAGCGGCAAAGACCGCCCTTGATGCGACCTCAGGTCAGGCGGGGCTACCCGCTGAGTTTAAGCATATCAATAAGCG

>Adenticulata_234

GTAACAAGGTTTCCGTAGGTGaAcCTGCGGAAGGAtCATTGTTGATGCCTCAACCCAGCTAGACCCGCGAATACGTTTTACTACCCGGGGTGATTGGGCTGCCTAGGCAGCTCGCCTCCCCGACCCGTTGGGGCTCTGGCCGCCCTGTGTGGCTCGGTCCCGACACAACAACAAACCCCGGCGCGGAATGCGCCAAGGAATAACAATCACAAGGCGTGCCCCCTCGACCCGGAAGCGGTGTTCGTCTGGGTGGCGTCGCAAAAAAATGAGTCCAAAATGACTCTCGGCAACGGATATCTCGGCTCTTGCATCGATGAAGAACGTAGCGAAATGCGATACTTGGTGTGAATTGCAGAATCCCGTGAACCATCGAGTCTTTGAACGCAAGTTGCGCCCGAAGCCATTAGGCTAAGGGCACGCCTGCCTGGGTGTCACCAATCGTCGCCCCCAACCTCACTGCCTCGTTGCGTGGGGAAGGGGTGAATGATGGCTTCCCGTGAGCACGGTCTCGCGGTTGGCTGAAAACGTTCTCCGTGCTGGCGTGCAGCGCCgTGACACTTGGTGGTTGAGTTTACCCTCGAGGCCAGTCACGTGTGCTCCCTGTCGGTTCCGGAAGCATGGACCCGTGAGCGGCAAAGACCGCCCTTGATGCGACCTCAGGTCAGGCGGGGCTACCCGCTGAGTTTAAGCATATCAaTAAGCG

>Adenticulata-238

GTAACAAGGTTTCCGTAGGTGAACcTGCGGAAGGATCATTGTTGATGCCTCAACCCAGCTAGACCCGCGAATACGTTTTACTACCTGGGGTGATCGGGCTGCCTAGGCAGCTCGCCTCCCCGACCCGTTGGGGCTCTGGCCGCCCTGTGTGGCTCGGTCCCGACACAACAACAAACCCCGGCGCGGAATGCGCCAAGGAATAACAATCACAAGGCGTGCCCCCTCGACCCGGAAGCGGTGTTCGTCTGGGTGGCGTCGCAAAAAATCGAGTCCAAAATGACTCTCGGCAACGGATATCTCGGCTCTTGCATCGATGAAGAACGTAGCGAAATGCGATACTTGGTGTGAATTGCAGAATCCCGTGAACCATCGAGTCTTTGAACGCAAGTTGCGCCCGAAGCCATTAGGCTAAGGGCACGCCTGCCTGGGTGTCACCAATCGTCGCCCCCAACCTCACTGCCTCGTTGCGTGGGGAAGGGGTGAATGATGGCTTCCCGTGAGCACGGTCTCGCGGTTGGCTGAAAACGTTCTCCGTGCTGGCGTGCAGCGCCGTGACACTTGGTGGTTGAGTTTACCCTCGAGGCCAGTCACGTGTGCTCCCTGTCGGTTCCGGAAGCATGGACCCGTGAGCGGCAAAGACCGCCCTTGATGCGACCTCAGGTCAGGCGGGGCTACCCGCTGAGTTTAAGCATATCAATAAGCG

>Adenticulata_254

GTAACAAGGTTTCCGTAGGTGAacCTGCGGAAGGAtCATTGTTGATGCCTCGACCCaGCTAGACCCGTGAATGCGTTTTACTACCCGGGGTGATCGGGCTGCCTAGGCAGCTCGCCTCCCTGACTCGTTGGGGCTCTGGCCGCCCTGTGTGGCCCGGTCCCGACACAACAACAAACCCCGGCGCGGAATGCGCCAAGGAATAACAATCACAAGGCGTGCCCCCTCGACCCGGAAGCGGTGTTCGTATGGGTGGCGTCGCAAAAAATTGAGTCCAAAATGACTCTCGGCAACGGATATCTCGGCTCTTGCATCGATGAAGAACGTAGCGAAATGCGATACTTGGTGTGAATTGCAGAATCCCGTGAACCATCGAGTCTTTGAACGCAAGTTGCGCCCGAAGCCATTAGGCTAAGGGCACGCCTGCCTGGGTGTCACCAATCGTCGCCCCCAACCTCACTGCCTCGTTGCGTGGGGAAGGGGTGAATGATGGCTTCCCGTGAGCACGGTCTCGCGGTTGGCTGAAAACGTTCTCCGTGCTGGCGTGCAGCGCCGTGACACTTGGTGGTTGAGTTTACCCTCGAGGCCAGTCACGTGTGCTCCCTGTCGGTTCCGGAAGCATGGACCCGTGAGCGGCAAAGACCGCTCTTGATGCGACCTCAGGTCAGGCGGGGCTACCCGCTGAGTTTAAGCATATCAATAAGCG

>Adenticulata-265

GTAACAAGGTTTCCGTAgGTGAacCTGCGGAAGGatCATTGTTGATGCCTCGACCCAGATAGACCCGCGAATGCGTTTTACTACCCGGGGTGATCGGGCTGCCTAGGCAGCTCGCCTCCCTGACTCGTTGGGGCTCTGGCCGCCCTGTGTGGCCCGGTCCCGACACAACAACAAACCCCGGCGCGGAATGCGCCAAGGAATAACAATCACAAGGCGTGCCCCCTCGACCCGGAAGCGGTGTTCGTATGGGTGGCGTCGCAAAAAATTGAGTCCAAAATGACTCTCGGCAACGGATATCTCGGCTCTTGCATCGATGAAGAACGTAGCGAAATGCGATACTTGGTGTGAATTGCAGAATCCCGTGAACCATCGAGTCTTTGAACGCAAGTTGCGCCCGAAGCCATTAGGCTAAGGGCACGCCTGCCTGGGTGTCACCAATCGTCGCCCCCAACCTCACTGCCTCGTTGCGTGGGGAAGGGGTGAATGATGGCTTCCCGTGAGCACGGTCTCGCGGTTGGCTGAAAACGTTCTCCGTGCTGGCGTGCAGCGCCGTGACACTTGGTGGTTGAGTTTACCCTCGAGGCCAGTCACGTGTGCTCCCTGTCGGTTCCGGAAGCATGGACCCGTGAGCGGCAAAGACCGCCCTTGATGCGACCTCAGGTCAGGCGGGGCTACCCGCTGAGTTTAAGCATATCAaTAAGCG

>Adenticulata_267

GTAACAAGGTTTCCGTAGGTGAacCTGCGGAAGGatcATTGTTGAtGCCTCAACCCaGCTAGACCCGCGAATACGTTTTACTACCCGGGGTGATTGGGCTGCCTAGGCAGCTCGCCTCCCCGACCCGTTGGGGCTCTGGCCGCCCTGTGTGGCTCGGTCCCGACACAACAACAAACCCCGGCGCGGAATGCGCCAAGGAATAACAATCACAAGGCGTGCCCCCTCGACCCGGAAGCGGTGTTCGTCTGGGTGGCGTCGCAAAAAAATGAGTCCAAAATGACTCTCGGCAACGGATATCTCGGCTCTTGCATCGATGAAGAACGTAGCGAAATGCGATACTTGGTGTGAATTGCAGAATCCCGTGAACCATCGAGTCTTTGAACGCAAGTTGCGCCCGAAGCCATTAGGCTAAGGGCACGCCTGCCTGGGTGTCACCAATCGTCGCCCCCAACCTCACTGCCTCGTTGCGTGGGGAAGGGGTGAATGATGGCTTCCCGTGAGCACGGTCTCGCGGTTGGCTGAAAACGTTCTCCGTGCTGGCGTGCAGCGCCGTGACACTTGGTGGTTGAGTTTACCCTCGAGGCCAGTCACGTGTGCTCCCTGTCGGTTCCGGAAGCATGGACCCGTGAGCGGCAAAGACCGCCCTTGATGCGACCTCAGGTCAGGCGGGGCTACCCGCTGAGTTTAAGCATATCAaTAAGCG

>Adenticulata_269

GTAACAAGGTTTCCGTAGGTGAaCCTGCGGAAGGatCATTGTTGATGCCTCAACCCAGCTAGACCCGCGAATACGTTTTACTACCCGGGGTGATTGGGCTGCCTAGGCAGCTCGCCTCCCCGACCCGTTGGGGCTCTGGCCGCCCTGTGTGGCTCGGTCCCGACACAACAACAAACCCCGGCGCGGAATGCGCCAAGGAATAACAATCACAAGGCGTGCCCCCTCGACCCGGAAGCGGTGTTCGTCTGGGTGGCGTCGCAAAAAAATGAGTCCAAAATGACTCTCGGCAACGGATATCTCGGCTCTTGCATCGATGAAGAACGTAGCGAAATGCGATACTTGGTGTGAATTGCAGAATCCCGTGAACCATCGAGTCTTTGAACGCAAGTTGCGCCCGAAGCCATTAGGCTAAGGGCACGCCTGCCTGGGTGTCACCAATCGTCGCCCCCAACCTCACTGCCTCGTTGCGTGGGGAAGGGGTGAATGATGGCTTCCCGTGAGCACGGTCTCGCGGTTGGCTGAAAACGTTCTCCGTGCTGGCGTGCAGCGCCGTGACACTTGGTGGTTGAGTTTACCCTCGAGGCCAGTCACGTGTGCTCCCTGTCGGTTCCGGAAGCATGGACCCGTGAGCGGCAAAGACCGCCCTTGATGCGACCTCAGGTCAGGCGGGGCTACCCGCTGAGTTTAAGCATATCAaTAAGCG

>Adenticulata_270

GTAACAAGGTTTCCGTAGGTGAACCTGCGGAAGGaTCATTGTTGATGCCTCGACCCAGCTAGACCCGTGAATGCGTTTTACTACCCGGGGTGATCGGGCTGCCTAGGCAGCTCGCCTCCCTGACTCGTTGGGGCTCTGGCCGCCCTGTGTGGCCCGGTCCcGACACAACAACAAACCCCGGCGCGGAATGCGCCAAGGAATAACAATCACAAGGCGTGCCCCCTCGACCCGGAAGCGGTGTTCGTATGGGTGGCGTCGCAAAAAATTGAGTCCAAAATGACTCTCGGCAACGGATATCTCGGCTCTTGCATCGATGAAGAACGTAGCGAAATGCGATACTTGGTGTGAATTGCAGAATCCCGTGAACCATCGAGTCTTTGAACGCAAGTTGCGCCCGAAGCCATTAGGCTAAGGGCACGCCTGCCTGGGTGTCACCAATCGTCGCCCCCAACCTCACTGCCTCGTTGCGTGGGGAAGGGGTGAATGATGGCTTCCCGTGAGCACGGTCTCGCGGTTGGCTGAAAACGTTCTCCGTGCTGGCGTGCAGCGCCGTGACACTTGGTGGTTGAGTTTACCCTCGAGGCCAGTCACGTGTGCTCCCTGTCGGTTCCGGAAGCATGGACCCGTGAGCGGCAAAGACCGCTCTTGATGCGACCTCAgGTCAgGCGGGGCTACCCGCTGAGTTTAAGCATATCAaTAAGCG

>Adenticulata_273-A

GTAACAAGGTTTCCGTAGGTGAaCCTGCGGAAGGAtCATTGTTGATgCCTCgACCCaGCTAGACCcGCGAATgCGTTTTACTACCCGGGGTGATCGGGCTGCCTAGGCAGCTCGCCTCCCCGACCCGTTGGGGCTCTGGCCGCCcTGTGTGGCcCGGTCCCGACACAACAACAAACCCCGGCGCGGAATGCGCCAAGGAATAACAATCACAAGGCGTGCCCCCTCGACCCGGAAGCGGTGTTCGTaTGGGTGGCGTCGCAaAAAATCGAGTCCAAAaTGACTCTCGGCAAcGGATATCTCGGCTCTTGCATCGATGAAGAACGTAGCGAAaTGCGATACTTGGTGTGAATTGCAGAATCCCGTGAACCATCGAGTCTTTGAACGCAAGTTGCGCCCGAAGCCATTAGGCTAAGGGCACGCCTGCCTGGGTGTCACCaATCGTCGCCCCCAACCTCACTGCCTCGTTGCGTGGGGAAGGGGTGAATGATGGCTTCCCGTGAGCACGGTCTCGCGGTTGGCTGAAAACGTTCTCCGTGCTGGCGTGCAGCGCCGTGACACTTGGTGGTTGAGTTTACCCTCGAGGCCAGTCACGTGTGCTCCCTGTCGGTTCCGGAAGCATGGACCCGTGAGCGGCAAAGACCGCCCTTGATGCGACCTCAGGTCAGGCGGGGCTACCCGCTGAGTTTAAGCATATCAATAAGCG

>Adenticulata_273-B

GTAACAAGGTTTCcGTAGGTGAACcTGCGGAAGGATCATTGTTGATgCCTCaACCCAGCTAGACCcGCGAaTaCGTTTTACTACCCGGGGTGATCGGGCTGCCTAGGCAGCTCGCCTCCCCGACCCGTTGGGGCTCTGGCCGCCcTGTGTGGCtCGGTCCCGACACAACAACAAACCCCGGCGCGGAATGCGCCAAGGAATAACAATCACAAGGCGTGCCCCCTCGACCCGGAAGCGGTGTTCGTcTGGGTGGCGTCGCAAAAAATCGAGTCCAAAATGACTCTCGGCAACGGATATCTCGGCTCTTGCATCGATGAAGAACGTAGCGAAATGCGATACTTGGTGTGAATTGCAGAATCCCGTGAACCATCGAGTCTTTGAACGCAAGTTGCGCCCGAAGCCATTAGGCTAAGGGCACGCCTGCCTGGGTGTCACCAATCGTCGCCCCCAACCTCACTGCCTCGTTGCGTGGGGAAGGGGTGAATGATGGCTTCCCGTGAGCACGGTCTCGCGGTTGGCTGAAAACGTTCTCCGTGCTGGCGTGCAGCGCCGTGACACTTGGTGGTTGAGTTTACCCTCGAGGCCAGTCACGTGTGCTCCCTGTCGGTTCCGGAAGCATGGACCCGTGAGCGGCAAAGACCGCCCTTGATGCGACCTCAGGTCAGGCGGGGCTACCCGCtGAGTTTAAGCATATCAATAAGCG

>Adenticulata_331

GTAACAAGGTTTCCGTAgGTGAACCTGCGGAaGGATCATTGTTGATGCCTCGACCCAGATAGACCCGCGAATGCGTTTTACTACCCGGGGTGATCGGGCTGCCTAGGCAGCTCGCCTCCCTGACTCGTTGGGGCTCTGGCCGCCCTGTGTGGCCCGGTCCCGACACAACAACAAACCCCGGCGCGGAATGCGCCAAGGAATAACAATCACAAGGCGTGCCCCCTCGACCCGGAAGCGGTGTTCGTATGGGTGGCGTCGCAAAAAATTGAGTCCAAAATGACTCTCGGCAACGGATATCTCGGCTCTTGCATCGATGAAGAACGTAGCGAAATGCGATACTTGGTGTGAATTGCAGAATCCCGTGAACCATCGAGTCTTTGAACGCAAGTTGCGCCCGAAGCCATTAGGCTAAGGGCACGCCTGCCTGGGTGTCACCAATCGTCGCCCCCAACCTCACTGCCTCGTTGCGTGGGGAAGGGGTGAATGATGGCTTCCCGTGAGCACGGTCTCGCGGTTGGCTGAAAACGTTCTCCGTGCTGGCGTGCAGCGCCGTGACACTTGGTGGTTGAGTTTACCCTCGAGGCCAGTCACGTGTGCTCCCTGTCGGTTCCGGAAGCATGGACCCGTGAGCGGCAAAGACCGCCCTTGATGCGACCTCAgGTCAgGCGGGGCTACCCGCTGAGTTTAAGCATATCAaTAAGCG

>Adenticulata_332

GTAACAAGGTTTCCgTAGGTGAACCTGCGGAAGGatCATTGTTGATGCCTCGACCCAGCTAGACCCGTGAATGCGTTTTACTACCCGGGGTGATCGGGCTGCCTAGGCAGCTCGCCTCCCTGACTCGTTGGGGCTCTGGCCGCCCTGTGTGGCCCGGTCCCGACACAACAACAAACCCCGGCGCGGAATGCGCCAAGGAATAACAATCACAAGGCGTGCCCCCTCGACCCGGAAGCGGTGTTCGTATGGGTGGCGTCGCAAAAAATTGAGTCCAAAATGACTCTCGGCAACGGATATCTCGGCTCTTGCATCGATGAAGAACGTAGCGAAATGCGATACTTGGTGTGAATTGCAGAATCCCGTGAACCATCGAGTCTTTGAACGCAAGTTGCGCCCGAAGCCATTAGGCTAAGGGCACGCCTGCCTGGGTGTCACCAATCGTCGCCCCCAACCTCACTGCCTCGTTGCGTGGGGAAGGGGTGAATGATGGCTTCCCGTGAGCACGGTCTCGCGGTTGGCTGAAAACGTTCTCCGTGCTGGCGTGCAGCGCCGTGACACTTGGTGGTTGAGTTTACCCTCGAGGCCAGTCACGTGTGCTCCCTGTCGGTTCCGGAAGCATGGACCCGTGAGCGGCAAAGACCGCCCTTGATGCGACCTCAGGTCAGGCGGGGCTACCCGCTGAGTTTAAGCATATCAATAAGCG

>Adenticulata_333

GTAACAAGGTTTCcGTAGGTGAaCCTGCGGAAGGaTCATTGTTGATGCCTCAACCCAGCTAGACCCGCGAATACGTTTTACTACCCGGGGTGATTGGGCTGCCTAGGCAGCTCGCCTCCTCGACCCGTTGGGGCTCTGGCCGCCCTGTGTGGCTCGGTCCCGACACAACAACAAACCCCGGCGCGGAATGCGCCAAGGAATATCAATCACAAGGCGTGCCCCCTCGACCCGGAAGCGGTGTTCGTCTGGGTGGCGTCGCAAAAAAATGAGTCCAAAATGACTCTCGGCAACGGATATCTCGGCTCTTGCATCGATGAAGAACGTAGCGAAATGCGATACTTGGTGTGAATTGCAGAATCCCGTGAACCATCGAGTCTTTGAACGCAAGTTGCGCCCGAAGCCATTAGGCTAAGGGCACGCCTGCCTGGGTGTCACCAATCGTCGCCCCCAACCTCACTGCCTCGTTGCGTGGGGAAGGGGTGAATGATGGCTTCCCGTGAGCACGGTCTCGCGGTTGGCTGAAAACGTTCTCCGTGCTGGCGTGCAGCGCCGTGACACTTGGTGGTTGAGTTTACCCTCGAGGCCAGTCACGTGTGCTCCCTGTCGGTTCCGGAAGCATGGACCCGTGAGCGGCAAAGACCGCCCTTGATGCGACCTCAGGTCAGGCGGGGCTACCCGCTGAGTTTAAGCATATCAATAAGCG

>Adenticulata_335

GTAACAAGGTTTCCgTAGGTGAaCCTGCGGAAGGatCATTGTTGATGCCTCAACCCAGCTAGACCCGCGAATACGTTTTACTACCCGGGGTGATTGGGCTGCCTAGGCAGCTCGCCTCCCCGACCCGTTGGGGCTCTGGCCGCCCTGTGTGGCTCGGTCCCGACACAACAACAAACCCCGGCGCGGAATGCGCCAAGGAATAACAATCACAAGGCGTGCCCCCTCGACCCGGAAGCGGTGTTCGTCTGGGTGGCGTCGCAAAAAAATGAGTCCAAAATGACTCTCGGCAACGGATATCTCGGCTCTTGCATCGATGAAGAACGTAGCGAAATGCGATACTTGGTGTGAATTGCAGAATCCCGTGAACCATCGAGTCTTTGAACGCAAGTTGCGCCCGAAGCCATTAGGCTAAGGGCACGCCTGCCTGGGTGTCACCAATCGTCGCCCCCAACCTCACTGCCTCGTTGCGTGGGGAAGGGGTGAATGATGGCTTCCCGTGAGCACGGTCTCGCGGTTGGCTGAAAACGTTCTCCGTGCTGGCGTGCAGCGCCGTGACACTTGGTGGTTGAGTTTACCCTCGAGGCCAGTCACGTGTGCTCCCTGTCGGTTCCGGAAGCATGGACCCGTGAGCGGCAAAGACCGCCCTTGATGCGACCTCAGGTCAGGCGGGGCTACCCGCTGAGTTTAAGCATATCAaTAAGCG

>Adenticulata_349

GTAACAAGGTTTCCgTAGGTGAaCCTGCGGAAGGatCATTGTTGATGCCTCGACCCAGCTAGACCCGtGAATGCGTTTTACTACCCGGGGTGATCGGGCTGCCTAGGCAGCTCGCCTCCCTGACTCGTTGGGGCTCTGGCCGCCCTGTGTGGCCCGGTCCCGACACAACAACAAACCCCGGCGCGGAATGCGCCAAGGAATAACAATCACAAGGCGTGCCCCCTCGACCCGGAAGCGGTGTTCGTATGGGTGGCGTCGCAAAAAATTGAGTCCAAAATGACTCTCGGCAACGGATATCTCGGCTCTTGCATCGATGAAGAACGTAGCGAAATGCGATACTTGGTGTGAATTGCAGAATCCCGTGAACCATCGAGTCTTTGAACGCAAGTTGCGCCCGAAGCCATTAGGCTAAGGGCACGCCTGCCTGGGTGTCACCAATCGTCGCCCCCAACCTCACTGCCTCGTTGCGTGGGGAAGGGGTGAATGATGGCTTCCCGTGAGCACGGTCTCGCGGTTGGCTGAAAACGTTCTCCGTGCTGGCGTGCAGCGCCGTGACACTTGGTGGTTGAGTTTACCCTCGAGGCCAGTCACGTGTGCTCCCTGTCGGTTCCGGAAGCATGGACCCGTGAGCGGCAAAGACCGCcCTTGATGCGACCTCAGGTCAGGCGGGGCTACCCGCTGAGTTTAAGCATATCAATAAGCG

>Adenticulata_351

GTAACAAGGTTTCCgTAGGTGAACCTGCGGAAGGatCATTGTTGATGCCTCAACCCAGCTAGACCCGCGAATACGTTTTACTACCCGGGGTGATCGGGCTGCCTAGGCAGCTCGCCTCCCCGACCCGTTGGGGCTCTGGCCGCCCTGTGTGGCTCGGTCCCGACACAACAACAAACCCCGGCGCGGAATGCGCCAAGGAATAACAATCACAAGGCGTGCCCCCTCGACCCGGAAGCGGTGTTCGTCTGGGTGGCGTCGCAAAAAATCGAGTCCAAAATGACTCTCGGCAACGGATATCTCGGCTCTTGCATCGATGAAGAACGTAGCGAAATGCGATACTTGGTGTGAATTGCAGAATCCCGTGAACCATCGAGTCTTTGAACGCAAGTTGCGCCCGAAGCCATTAGGCTAAGGGCACGCCTGCCTGGGTGTCACCAATcGTCGCCCCCAACCTCACTGCCTCGTTGCGTGGGGAAGGGGTGAATGATGGCTTCCCGTGAGCACGGTCTCGCGGTTGGCTGAAAACGTTCTCCGTGCTGGCGTGCAGCGCCGTGACACTTGGTGGTTGAGTTTACCCTCGAGGCCAGTCACGTGTGCTCCCTGTCGGTTCCGGAAGCATGGACCCGTGAGCGGCAAAGACCGCCCTTGATGCGACCTCAgGTCAGGCGGGGCTACCCGCTGAGTTTAAGCATATCAaTAAGCG

>Adenticulata_352

GTAACAAGGTTTCCgTAGGTGAaCCTGCGGAAGGatCATTGTTGATGCCTCGACCCaGCTAGACCCGTGAATGCGTTTTACTACCCGGGGTGATCGGGCTGCCTAGGCAGCTCGCCTCCCTGACTCGTTGGGGCTCTGGCCGCCCTGTGTGGCCCGGTCCCGACACAACAACAAACCCCGGCGCGGAATGCGCCAAGGAATAACAATCACAAGGCGTGCCCCCTCGACCCGGAAGCGGTGTTCGTATGGGTGGCGTCGCAAAAAATTGAGTCCAAAATGACTCTCGGCAACGGATATCTCGGCTCTTGCATCGATGAAGAACGTAGCGAAATGCGATACTTGGTGTGAATTGCAGAATCCCGTGAACCATCGAGTCTTTGAACGCAAGTTGCGCCCGAAGCCATTAGGCTAAGGGCACGCCTGCCTGGGTGTCACCAATCGTCGCCCCCAACCTCACTGCCTCGTTGCGTGGGGAAGGGGTGAATGATGGCTTCCCGTGAGCACGGTCTCGCGGTTGGCTGAAAACGTTCTCCGTGCTGGCGTGCAGCGCCGTGACACTTGGTGGTTGAGTTTACCCTCGAGGCCAGTCACGTGTGCTCCCTGTCGGTTCCGGAAGCATGGACCCGTGAGCGGCAAAGACCGCCCTTGATGCGACCTCAGGTCAGGCGGGGCTACCCGCTGAGTTTAAGCATATCAaTAAGCG

>Aevenia_ssp_evenia-21-A

GTAACAAGGTTTCCGTAGGTGAACCTGCGGAAGGATCATTGTTGATGCCTCGACCCAGCTAGACCCGCGAATGTGTTTTACAACTCGGGGTGATCGGGTTGCCTAGGCAGCTCGCCTCCCCGACCCGTTGGGGCTCTTGCCACCCTGTGTGGCTCGGTCCCGACACAACAACAAACCCCGGCGCGGAATGCGCCAAGGAATCACAAACACAAGGCGTGCCCCCTCGACCCGGAAGCGGTGTTCGTCTGGGTGGCGTCGCAAAAAATTGAGTCCAAAATGACTCTCGGCAACGGATATCTCGGCTCTTGCATCGATGAAGAACGTAGCGAAATGCGATACTTGGTGTGAATTGCAGAATCCCGTGAACCATCGAGTCTTTGAACGCAAGTTGCGCCCGAAGCCATTAGGCTAAGGGCACGCCTGCCTGGGTGTCACCAATCGTCGCCCCCAACCTCACTGCCTTGTTGCGTGGAGAAGGGGTGAATGATGGCTTCCCGTGAGCACAGTCTCGCGGTTGGCTGAAAACGTTCTCCGTGCCGGCGTGCAGTGCCGTGACACTTGGTGGTTGAGTTTACCCTCGAGGCCAGTCACGTGTGCTCCCTGTCGGTTCCGGAAGCATGGACCCGTGAGCGGCAAAGACCGCCCTTGATGCGACCTCAGGTCAGGCGGGGCTACCCGCTGAGTTTAAGCATATCAATAAGCG

>Aevenia_ssp_evenia-21-B

GTAACAAGGTTTCCGTAGGTGAACCTGCGGAAGGATCATTGTTGATGCCTCGACCCAGCTAGACCCGCGAATGTGTTTTACTACCCGGGGTGATCGGGATGCCTAGGCAGCTCGCCTCCCCGACCCGTTGGGGCTCTGGCCACCCTGTGTGGCTCGGTCCCGACACAACAACAAACCCCGGCGCGGAATGCGCCAAGGAATAACAAACACAAGGCGTGCCCCCTCGACCCGGAAGCGGTGTTCGTCTGGGTGGCGTCGCAAAAAATTGAGTCCAAAATGACTCTCGGCAACGGATATCTCGGCTCTTGCATCGATGAAGAACGTAGCGAAATGCGATACTTGGTGTGAATTGCAGAATCCCGTGAACCATCGAGTCTTTGAACGCAAGTTGCGCCCGAAGCCATTAGGCTAAGGGCACGCCTGCCTGGGTGTCACCAATCGTCGCCCCCAACCTCACTGCCTTGTTGCGTGGAGAAGGGGTGAATGATGGCTTCCCGTGAGCACAGTCTCGCGGTTGGCTGAAGACGTTCTCCGTGCCGGCGTGCAGTGCCGTGACACTTGGTGGTTGAGTTTACCCTCGAGGCCAGTCACGTGTGCTCCCTGTCGGTTCCGGAAGCATGGACCCGTGAGCGGCAAAGACCGCCCTTGATGCGACCTCAGGTCAGGCGGGGCTACCCGCTGAGTTTAAGCATATCAATAAGCG

>Aevenia_ssp_evenia-29

GTAACAAGGTTTCCGTAGGTGAACCTGCGGAAGGATCATTGTTGATGCCTCGACCCAGCTAGAcCCgcgAATGTGTTTTACAACTCGGGGTGATCGGGTTGCCTGGGCAGCTCGCCTCCCCGACCCGTTGGGGCTCTTTCCACCCTGTGTGGCTCGGTCCCGACACAACAACAAACCCCGGCGCGGAATGCGCCAAGGAATCACAAACACAAGGCGTGCCCCCTCGACCCGGAAGCGGTGTTCGTCTGGGTGGCGTCGCAAAAAATTGAGTCCAAAATGACTCTCGGCAACGGATATCTCGGCTCTTGCATCGATGAAGAACGTAGCGAAATGCGATACTTGGTGTGAATTGCAGAATCCCGTGAACCATCGAGTCTTTGAACGCAAGTTGCGCCCGAAGCCATTAGGCTAAGGGCACGCCTGCCTGGGTGTCACCAATCGTCGCCCCCAACCTCACTGCCTTGTTGCGTGGAGAAGGGGTGAATGATGGCTTCCCGTGAGCACAGTCTCGCGGTTGGCTGAAAACGTTCTCCGTGCCGGCGTGCAGTGCCGTGACACTTGGTGGTTGAGTTTACCCTCGAGGCCAGTCACGTGTGCTCCCTGTCGGTTCCGGAAGCATGGACCCGTGAGCGGCAAAGACCGCCCTTGATGCGACCTCAGGTCAGGCGGGGCTACCCGCTGAGTTTAAGCATATCAATAAGCG

>Aevenia_ssp_evenia-45

GTAACAAGGTTTCCGTAGGTGAACCTGCGGAAGGATCATTGTTGATGCCTCGACCCAGCTAGACCCGCGAATGTGTTTTACTACCCGGGGTGATCGGGCTGCCTAGGCAGCTCGCCTCCCCGACCCGTTGGGGCTCTGGCCACCCTGTGTGGCTCGGTCCCGACACAACAACAAACCCCGGCGCGGAATGCGCCAAGGAATCACAAACACAAGGCGTGCCCCCTCGACCCGGAAGCGGTGTTCGTCTGGGTGGCGTCGCAAAAAATTGAGTCCAAAATGACTCTCGGCAACGGATATCTCGGCTCTTGCATCGATGAAGAACGTAGCGAAATGCGATACTTGGTGTGAATTGCAGAATCCCGTGAACCATCGAGTCTTTGAACGCAAGTTGCGCCCGAAGCCATTAGGCTAAGGGCACGCCTGCCTGGGTGTCACCAATCGTCGCCCCCAACCTCACTGCCTTGTTGCGTGGAGAAGGGGTGAATGATGGCTTCCCGTGAGCACAGTCTCGCGGTTGGCTGAAAACGTTCTCCGTGCCGGCGTGCAGCGCCGTGACACTTGGTGGTTGAGTTTACCCTCGAGGCCAGTCACGTGTGCTCCCTGTCGGTTCCGGAAGCATGGACCCGTGAGCGGCAAAGACCGCCCTTGATGCGACCTCAGGTCAGGCGGGGCTACCCGCTGAGTTTAAGCATATCAATAAGCG

>Aevenia_ssp_evenia-57-A

GTAACAAGGTTTCCGTAGGTGAACCTGCGGAAGGATCATTGTTGATGCCTCGACCCAGCTAGACCCGCGAATGTGTTTTACAACTCGGGGTGATCGGGTTGCCTAGGCAGCTCGCCTCCCCGACCCGTTGGGGCTCTTGCCACCCTGTGTGGCTCGGTCCCGACACAACAACAAACCCCGGCGCGGAATGCGCCAAGGAATCACAAACACAAGGCGTGCCCCCTCGACCCGGAAGCGGTGTTCGTCTGGGTGGCGTCGCAAAAAATTGAGTCCAAAATGACTCTCGGCAACGGATATCTCGGCTCTTGCATCGATGAAGAACGTAGCGAAATGCGATACTTGGTGTGAATTGCAGAATCCCGTGAACCATCGAGTCTTTGAACGCAAGTTGCGCCCGAAGCCATTAGGCTAAGGGCACGCCTGCCTGGGTGTCACCAATCGTCGCCCCCAACCTCACTGCCTTGTTGCGTGGAGAAGGGGTGAATGATGGCTTCCCGTGAGCACAGTCTCGCGGTTGGCTGAAAACGTTCTCCGTGCCGGCGTGCAGTGCCGTGACACTTGGTGGTTGAGTTTACCCTCGAGGCCAGTCACGTGTGCTCCCTGTCGGTTCCGGAAGCATGGACCCGTGAGCGGCAAAGACCGCCCTTGATGCGACCTCAGGTCAGGCGGGGCTACCCGCTGAGTTTAAGCATATCAATAAGCG

>Aevenia_ssp_evenia-57-B

GTAACAAGGTTTCCGTAGGTGAACCTGCGGAAGGATCATTGTTGATGCCTCGACCCAGCTAGACCCGCGAATGTGTTTTACTACCCGGGGTGATCGGGATGCCTAGGCAGCTCGCCTCCCCGACCCGTTGGGGCTCTGGCCACCCTGTGTGGCTCGGTCCCGACACAACAACAAACCCCGGCGCGGAATGCGCCAAGGAATAACAAACACAAGGCGTGCCCCCTCGACCCGGAAGCGGTGTTCGTCTGGGTGGCGTCGCAAAAAATTGAGTCCAAAATGACTCTCGGCAACGGATATCTCGGCTCTTGCATCGATGAAGAACGTAGCGAAATGCGATACTTGGTGTGAATTGCAGAATCCCGTGAACCATCGAGTCTTTGAACGCAAGTTGCGCCCGAAGCCATTAGGCTAAGGGCACGCCTGCCTGGGTGTCACCAATCGTCGCCCCCAACCTCACTGCCTTGTTGCGTGGAGAAGGGGTGAATGATGGCTTCCCGTGAGCACAGTCTCGCGGTTGGCTGAAGACGTTCTCCGTGCCGGCGTGCAGTGCCGTGACACTTGGTGGTTGAGTTTACCCTCGAGGCCAGTCACGTGTGCTCCCTGTCGGTTCCGGAAGCATGGACCCGTGAGCGGCAAAGACCGCCCTTGATGCGACCTCAGGTCAGGCGGGGCTACCCGCTGAGTTTAAGCATATCAATAAGCG

>Aevenia_ssp_evenia-75

GTAACAAGGTTTCCGTAGGTGAACCTGCGGAAGGATCATTGTTGATGCCTCGACCCAGCTAGACCCGCGAATGTGTTTTACAACTCGGGGTGATCGGGTTGCCTGGGCAGCTCGCCTCCCCGACCCGTTGGGGCTCTTTCCACCCTGTGTGGCTCGGTCCCGACACAACAACAAACCCCGGCGCGGAATGCGCCAAGGAATCACAAACACAAGGCGTGCCCCCTCGACCCGGAAGCGGTGTTCGTCTGGGTGGCGTCGCAAAAAATTGAGTCCAAAATGACTCTCGGCAACGGATATCTCGGCTCTTGCATCGATGAAGAACGTAGCGAAATGCGATACTTGGTGTGAATTGCAGAATCCCGTGAACCATCGAGTCTTTGAACGCAAGTTGCGCCCGAAGCCATTAGGCTAAGGGCACGCCTGCCTGGGTGTCACCAATCGTCGCCCCCAACCTCACTGCCTTGTTGCGTGGAGAAGGGGTGAATGATGGCTTCCCGTGAGCACAGTCTCGCGGTTGGCTGAAAACGTTCTCCGTGCCGGCGTGCAGTGCCGTGACACTTGGTGGTTGAGTTTACCCTCGAGGCCAGTCACGTGTGCTCCCTGTCGGTTCCGGAAGCATGGACCCGTGAGCGGCAAAGACCGCCCTTGATGCGACCTCAGGTCAGGCGGGGCTACCCGCTGAGTTTAAGCATATCAATAAGCG

>Aevenia_ssp_evenia-76-A

GTAACAAGGTTTCCGTAGGTGAACCTGCGGAAGGATCATTGTTGATGCCTCGACCCAGCTAGACCCGCGAATGTGTTTTACAACTCGGGGTGATCGGGTTGCCTAGGCAGCTCGCCTCCCCGACCCGTTGGGGCTCTTGCCACCCTGTGTGGCTCGGTCCCGACACAACAACAAACCCCGGCGCGGAATGCGCCAAGGAATCACAAACACAAGGCGTGCCCCCTCGACCCGGAAGCGGTGTTCGTCTGGGTGGCGTCGCAAAAAATTGAGTCCAAAATGACTCTCGGCAACGGATATCTCGGCTCTTGCATCGATGAAGAACGTAGCGAAATGCGATACTTGGTGTGAATTGCAGAATCCCGTGAACCATCGAGTCTTTGAACGCAAGTTGCGCCCGAAGCCATTAGGCTAAGGGCACGCCTGCCTGGGTGTCACCAATCGTCGCCCCCAACCTCACTGCCTTGTTGCGTGGAGAAGGGGTGAATGATGGCTTCCCGTGAGCACAGTCTCGCGGTTGGCTGAAAACGTTCTCCGTGCCGGCGTGCAGTGCCGTGACACTTGGTGGTTGAGTTTACCCTCGAGGCCAGTCACGTGTGCTCCCTGTCGGTTCCGGAAGCATGGACCCGTGAGCGGCAAAGACCGCCCTTGATGCGACCTCAGGTCAGGCGGGGCTACCCGCTGAGTTTAAGCATATCAATAAGCG

>Aevenia_ssp_evenia-76-B

GTAACAAGGTTTCCGTAGGTGAACCTGCGGAAGGATCATTGTTGATGCCTCGACCCAGCTAGACCCGCGAATGTGTTTTACTACCCGGGGTGATCGGGATGCCTAGGCAGCTCGCCTCCCCGACCCGTTGGGGCTCTGGCCACCCTGTGTGGCTCGGTCCCGACACAACAACAAACCCCGGCGCGGAATGCGCCAAGGAATAACAAACACAAGGCGTGCCCCCTCGACCCGGAAGCGGTGTTCGTCTGGGTGGCGTCGCAAAAAATTGAGTCCAAAATGACTCTCGGCAACGGATATCTCGGCTCTTGCATCGATGAAGAACGTAGCGAAATGCGATACTTGGTGTGAATTGCAGAATCCCGTGAACCATCGAGTCTTTGAACGCAAGTTGCGCCCGAAGCCATTAGGCTAAGGGCACGCCTGCCTGGGTGTCACCAATCGTCGCCCCCAACCTCACTGCCTTGTTGCGTGGAGAAGGGGTGAATGATGGCTTCCCGTGAGCACAGTCTCGCGGTTGGCTGAAGACGTTCTCCGTGCCGGCGTGCAGTGCCGTGACACTTGGTGGTTGAGTTTACCCTCGAGGCCAGTCACGTGTGCTCCCTGTCGGTTCCGGAAGCATGGACCCGTGAGCGGCAAAGACCGCCCTTGATGCGACCTCAGGTCAGGCGGGGCTACCCGCTGAGTTTAAGCATATCAATAAGCG

>Aevenia_ssp_evenia-78

GTAACAAGGTTTCCGTAGGTGAACCTGCGGAAGGATCATTGTTGATGCCTCGACCCAGCTAGACCCGCGAATGTGTTTTACAACTCGGGGTGATCGGGTTGCCTAGGCAGCTCGCCTCCCCGACCCGTTGGGGCTCTGGCCACCMTGTGTGGCTCGGTCCCGACACAACAACAAACCCCGGCGCGGAATGCGCCAAGGAATCACAAACACAAGGCGTGCCCCCTCGACCCGGAAGCGGTGTTCGTCTGGGTGGYGTCGCAAAAAATTGAGTCCAAAATGACTCTCGGCAACGGATATCTCGGCTCTTGCATCGATGAAGAACGTAGCGAAATGCGATACTTGGTGTGAATTGCAGAATCCCGTGAACCATCGAGTCTTTGAACGCAAGTTGCGCCCGAAGCCATTAGGCTAAGGGCACGCCTGCCTGGGTGTCACCAATCGTCGCCCCCAACCTCACTGCCTTGTTGCGTGGAGAAGGGGTGAATGATGGCTTCCCGTGAGCACAGTCTCGCGGTTGGCTGAAAACGTTCTCCGTGCCGGCGTGCAGTGCCGTGACACTTGGTGGTTGAGTTTACCCTCGAGGCCAGTCACGTGTGCTCCCTGTCGGTTCCGGAAGCATGGACCCGTGAGCGGCAAAGACCGCCCTTGATGCGACCTCAGGTCAGGCGGGGCTACCCGCTGAGTTTAAGCATATCAATAAGCG

>Aevenia_ssp_evenia-79

GTAACAAGGTTTCCGTAGGTGAACCTGCGGAAGGATCATTGTTGATGCCTCGACCCAGCTAGACCCGCGAATGTGTTTTACAACTCGGGGTGATCGGGTTGCCTAGGCAGCTCGCCTCCCCGACCCGTTGGGGCTCTGGCCACCCTGTGTGGCTCGGTCCCGACACAACAACAAACCCCGGCGCGGAATGCGCCAAGGAATCACAAACACAAGGCGTGCCCCCTCGACCCGGAAGCGGTGTTCGTCTGGGTGGTGTCGCAAAAAATTGAGTCCAAAATGACTCTCGGCAACGGATATCTCGGCTCTTGCATCGATGAAGAACGTAGCGAAATGCGATACTTGGTGTGAATTGCAGAATCCCGTGAACCATCGAGTCTTTGAACGCAAGTTGCGCCCGAAGCCATTAGGCTAAGGGCACGCCTGCCTGGGTGTCACCAATCGTCGCCCCCAACCTCACTGCCTTGTTGCGTGGAGAAGGGGTGAATGATGGCTTCCCGTGAGCACAGTCTCGCGGTTGGCTGAAAACGTTCTCCGTGCCGGCGTGCAGTGCCGTGACACTTGGTGGTTGAGTTTACCCTCGAGGCCAGTCACGTGTGCTCCCTGTCGGTTCCGGAAGCATGGACCCGTGAGCGGCAAAGACCGCCCTTGATGCGACCTCAGGTCAGGCGGGGCTACCCGCTGAGTTTAAGCATATCAATAAGCG

>Aevenia_ssp_evenia-80

GTAACAAGGTTTCCGTAGGTGAACCTGCGGAAGGATCATTGTTGATGCCTCGACCCAGCTAGACCCGCRAATGTGTTTTACAACTCGGGGTGATCGGGTTGCCTWGGCAGCTCGCCTCCCCGACCCGTTGGGGCTCTGGCCACCCTGTGTGGCTCGGTCCCGACACAACAACAAACCCCGGCGCGGAATGCGCCAAGGAATCACAAACACAAGGCGTGCCCCCTCGACCCGGAAGCGGTGTTCGTCTGGGTGGYGTCGCAAAAAATTGAGTCCAAAATGACTCTCGGCAACGGATATCTCGGCTCTTGCATCGATGAAGAACGTAGCGAAATGCGATACTTGGTGTGAATTGCAGAATCCCGTGAACCATCGAGTCTTTGAACGCAAGTTGCGCCCGAAGCCATTAGGCTAAGGGCACGCCTGCCTGGGTGTCACCAATCGTCGCCCCCAACCTCACTGCCTTGTTGCGTGGAGAAGGGGTGAATGATGGCTTCCCGTGAGCACAGTCTCGCGGTTGGCTGAAAACGTTCTCCGTGCCGGCGTGCAGTGCCGTGACACTTGGTGGTTGAGTTTACCCTCGAGGCCAGTCAYGTGTGCTCCCTGTCGGTTCCGGAAGCATGGACCCGTGAGCGGCAAAGACCGCCCTTGATGCGACCTCAGGTCAGGCGGGGCTACCCGCTGAGTTTAAGCATATCAATAAGCG

>Aevenia_ssp_evenia–81

GTAACAAGGTTTCCGTAGGTGAACCTGCGGAAGGATCATTGTTGATGCCTCGACCCAGCTAGACCCGCGAATGTGTTTTACAACTCGGGGTGATCGGGTTGCCTAGGCAGCTCGCCTCCCCGACCCGTTGGGGCTCTGGCCACCCTGTGTGGCTCGGTCCCGACACAACAACAAACCCCGGCGCGGAATGCGCCAAGGAATCACAAACACAAGGCGTGCCCCCTCGACCCGGAAGCGGTGTTCGTCTGGGTGGTGTCGCAAAAAATTGAGTCCAAAATGACTCTCGGCAACGGATATCTCGGCTCTTGCATCGATGAAGAACGTAGCGAAATGCGATACTTGGTGTGAATTGCAGAATCCCGTGAACCATCGAGTCTTTGAACGCAAGTTGCGCCCGAAGCCATTAGGCTAAGGGCACGCCTGCCTGGGTGTCACCAATCGTCGCCCCCAACCTCACTGCCTTGTTGCGTGGAGAAGGGGTGAATGATGGCTTCCCGTGAGCACAGTCTCGCGGTTGGCTGAAAACGTTCTCCGTGCCGGCGTGCAGTGCCGTGACACTTGGTGGTTGAGTTTACCCTCGAGGCCAGTCACGTGTGCTCCCTGTCGGTTCCGGAAGCATGGACCCGTGAGCGGCAAAGACCGCCCTTGATGCGACCTCAGGTCAGGCGGGGCTACCCGCTGAGTTTAAGCATATCAATAAGCG

>Aevenia_ssp_evenia-83

GTAACAAGGTTTCCGTAGGTGAACCTGCGGAAGGATCATTGTTGATGCCTCGACCCAGCTAGACCCGCGAATGTGTTTTACAACTCGGGGTGATCGGGTTGCCTAGGCAGCTCGCCTCCCCGACCCGTTGGGGCTCTGGCCACCCTGTGTGGCTCGGTCCCGACACAACAACAAACCCCGGCGCGGAATGCGCCAAGGAATCACAAACACAAGGCGTGCCCCCTCGACCCGGAAGCGGTGTTCGTCTGGGTGGTGTCGCAAAAAATTGAGTCCAAAATGACTCTCGGCAACGGATATCTCGGCTCTTGCATCGATGAAGAACGTAGCGAAATGCGATACTTGGTGTGAATTGCAGAATCCCGTGAACCATCGAGTCTTTGAACGCAAGTTGCGCCCGAAGCCATTAGGCTAAGGGCACGCCTGCCTGGGTGTCACCAATCGTCGCCCCCAACCTCACTGCCTTGTTGCGTGGAGAAGGGGTGAATGATGGCTTCCCGTGAGCACAGTCTCGCGGTTGGCTGAAAACGTTCTCCGTGCCGGCGTGCAGTGCCGTGACACTTGGTGGTTGAGTTTACCCTCGAGGCCAGTCACGTGTGCTCCCTGTCGGTTCCGGAAGCATGGACCCGTGAGCGGCAAAGACCGCCCTTGATGCGACCTCAGGTCAGGCGGGGCTACCCGCTGAGTTTAAGCATATCAATAAGCG

>Aevenia_ssp_evenia-87

GTAACAAGGTTTCCGTAGGTGAACCTGCGGAAGGATCATTGTTGATGCCTCGACCCAGCTAGACCCGCGAATGTGTTTTACAACTCGGGGTGATCGGGTTGCCTAGGCAGCTCGCCTCCCCGACCCGTTGGGGCTCTGGCCACCCTGTGTGGCTCGGTCCCGACACAACAACAAACCCCGGCGCGGAATGCGCCAAGGAATCACAAACACAAGGCGTGCCCCCTCGACCCGGAAGCGGTGTTCGTCTGGGTGGCGTCGCAAAAAATTGAGTCCAAAATGACTCTCGGCAACGGATATCTCGGCTCTTGCATCGATGAAGAACGTAGCGAAATGCGATACTTGGTGTGAATTGCAGAATCCCGTGAACCATCGAGTCTTTGAACGCAAGTTGCGCCCGAAGCCATTAGGCTAAGGGCACGCCTGCCTGGGTGTCACCAATCGTCGCCCCCAACCTCACTGCCTTGTTGCGTGGAGAAGGGGTGAATGATGGCTTCCCGTGAGCACAGTCTCGCGGTTGGCTGAAAACGTTCTCCGTGCCGGCGTGCAGTGCCGTGACACTTGGTGGTTGAGTTTACCCTCGAGGCCAGTCACGTGTGCTCCCTGTCGGTTCCGGAAGCATGGACCCGTGAGCGGCAAAGACCGCCCTTGATGCGACCTCAGGTCAGGCGGGGCTACCCGCTGAGTTTAAGCATATCAATAAGCG

>Aevenia_ssp_evenia-89

GTAACAAGGTTTCCGTAGGTGAACCTGCGGAAGGATCATTGTTGATGCCTCGACCCAGCTAGACCCGCGAATGTGTTTTACAACTCGGGGTGATCGGGTTGCCTAGGCAGCTCGCCTCCCCGACCCGTTGGGGCTCTGGCCACCCTGTGTGGCTCGGTCCCGACACAACAACAAACCCCGGCGCGGAATGCGCCAAGGAATCACAAACACAAGGCGTGCCCCCTCGACCCGGAAGCGGTGTTCGTCTGGGTGGTGTCGCAAAAAATTGAGTCCAAAATGACTCTCGGCAACGGATATCTCGGCTCTTGCATCGATGAAGAACGTAGCGAAATGCGATACTTGGTGTGAATTGCAGAATCCCGTGAACCATCGAGTCTTTGAACGCAAGTTGCGCCCGAAGCCATTAGGCTAAGGGCACGCCTGCCTGGGTGTCACCAATCGTCGCCCCCAACCTCACTGCCTTGTTGCGTGGAGAAGGGGTGAATGATGGCTTCCCGTGAGCACAGTCTCGCGGTTGGCTGAAAACGTTCTCCGTGCCGGCGTGCAGTGCCGTGACACTTGGTGGTTGAGTTTACCCTCGAGGCCAGTCACGTGTGCTCCCTGTCGGTTCCGGAAGCATGGACCCGTGAGCGGCAAAGACCGCCCTTGATGCGACCTCAGGTCAGGCGGGGCTACCCGCTGAGTTTAAGCATATCAATAAGCG

>Aevenia_ssp_evenia-90

GTAACAAGGTTTCCGTAGGTGAACCTGCGGAAGGATCATTGTTGATGCCTCGACCCAGCTAGACCCGCGAATGTGTTTTACAACTCGGGGTGATCGGGTTGCCTAGGCAGCTCGCCTCCCCGACCCGTTGGGGCTCTGGCCACCCTGTGTGGCTCGGTCCCGACACAACAACAAACCCCGGCGCGGAATGCGCCAAGGAATCACAAACACAAGGCGTGCCCCCTCGACCCGGAAGCGGTGTTCGTCTGGGTGGCGTCGCAAAAAATTGAGTCCAAAATGACTCTCGGCAACGGATATCTCGGCTCTTGCATCGATGAAGAACGTAGCGAAATGCGATACTTGGTGTGAATTGCAGAATCCCGTGAACCATCGAGTCTTTGAACGCAAGTTGCGCCCGAAGCCATTAGGCTAAGGGCACGCCTGCCTGGGTGTCACCAATCGTCGCCCCCAACCTCACTGCCTTGTTGCGTGGAGAAGGGGTGAATGATGGCTTCCCGTGAGCACAGTCTCGCGGTTGGCTGAAAACGTTCTCCGTGCCGGCGTGCAGTGCCGTGACACTTGGTGGTTGAGTTTACCCTCGAGGCCAGTCACGTGTGCTCCCTGTCGGTTCCGGAAGCATGGACCCGTGAGCGGCAAAGACCGCCCTTGATGCGACCTCAGGTCAGGCGGGGCTACCCGCTGAGTTTAAGCATATCAATAAGCG

>Aevenia_ssp_evenia-101

GTAACAAGGTTTCCGTAGGTGAACCTGCGGAAGGATCATTGTTGATGCCTCGACCCAGCTAGACCCGCGAATGTGTTTTACAACTCGGGGTGATCGGGTTGCCTAGGCAGCTCGCCTCCCCGAMYCGTTGGGGCTCTGGCCACCCTGTGTGGCTCGGTCCCGACACAACAACAAACCCCGGCGCGGAATGCGCCAAGGAATCACAAACACAAGGCGTGCCCCCTCGACCCGGAAGCGGTGTTCGTCTGGGTGGCGTCGCAAAAAATTGAGTCCAAAATGACTCTCGGCAACGGATATCTCGGCTCTTGCATCGATGAAGAACGTAGCGAAATGCGATACTTGGTGTGAATTGCAGAATCCCGTGAACCATCGAGTCTTTGAACGCAAGTTGCGCCCGAAGCCATTAGGCTAAGGGCACGCCTGCCTGGGTGTCACCAATCGTCGCCCCYAACCTCACTGCCTTGTTGCGTGGAGAAGGGGTGAATGATGGCTTCCCGTGAGCACAGTCTCGCGGTTGGCTGAAAACGTTCTCCGTGCCGGCGTGCAGTGCCGTGACACTTGGTGGTTGAGTTTACCCTCGAGGCCAGTCACGTGTGCTCCCTGTCGGTTCCGGAAGCATGGACCCGTGAGCGGCAAAGACCGCCCTTGATGCGACCTCAGGTCAGGCGGGGCTACCCGCTGAGTTTAAGCATATCAATAAGCG

>Aevenia_ssp_evenia-107-A

GTAACAAGGTTTCCGTAGGTGAACCTGCGGAAGGATCATTGTTGATGCCTCGACCCAGCTAGACCCGCGAATGTGTTTTACAACTCGGGGTGATCGGGTTGCCTAGGCAGCTCGCCTCCCCGACCCGTTGGGGCTCTTGCCACCCTGTGTGGCTCGGTCCCGACACAACAACAAACCCCGGCGCGGAATGCGCCAAGGAATCACAAACACAAGGCGTGCCCCCTCGACCCGGAAGCGGTGTTCGTCTGGGTGGCGTCGCAAAAAATTGAGTCCAAAATGACTCTCGGCAACGGATATCTCGGCTCTTGCATCGATGAAGAACGTAGCGAAATGCGATACTTGGTGTGAATTGCAGAATCCCGTGAACCATCGAGTCTTTGAACGCAAGTTGCGCCCGAAGCCATTAGGCTAAGGGCACGCCTGCCTGGGTGTCACCAATCGTCGCCCCCAACCTCACTGCCTTGTTGCGTGGAGAAGGGGTGAATGATGGCTTCCCGTGAGCACAGTCTCGCGGTTGGCTGAAAACGTTCTCCGTGCCGGCGTGCAGTGCCGTGACACTTGGTGGTTGAGTTTACCCTCGAGGCCAGTCACGTGTGCTCCCTGTCGGTTCCGGAAGCATGGACCCGTGAGCGGCAAAGACCGCCCTTGATGCGACCTCAGGTCAGGCGGGGCTACCCGCTGAGTTTAAGCATATCAATAAGCG

>Aevenia_ssp_evenia-107-B

GTAACAAGGTTTCCGTAGGTGAACCTGCGGAAGGATCATTGTTGATGCCTCGACCCAGCTAGACCCGCGAATGTGTTTTACTACCCGGGGTGATCGGGATGCCTAGGCAGCTCGCCTCCCCGACCCGTTGGGGCTCTGGCCACCCTGTGTGGCTCGGTCCCGACACAACAACAAACCCCGGCGCGGAATGCGCCAAGGAATAACAAACACAAGGCGTGCCCCCTCGACCCGGAAGCGGTGTTCGTCTGGGTGGCGTCGCAAAAAATTGAGTCCAAAATGACTCTCGGCAACGGATATCTCGGCTCTTGCATCGATGAAGAACGTAGCGAAATGCGATACTTGGTGTGAATTGCAGAATCCCGTGAACCATCGAGTCTTTGAACGCAAGTTGCGCCCGAAGCCATTAGGCTAAGGGCACGCCTGCCTGGGTGTCACCAATCGTCGCCCCCAACCTCACTGCCTTGTTGCGTGGAGAAGGGGTGAATGATGGCTTCCCGTGAGCACAGTCTCGCGGTTGGCTGAAGACGTTCTCCGTGCCGGCGTGCAGTGCCGTGACACTTGGTGGTTGAGTTTACCCTCGAGGCCAGTCACGTGTGCTCCCTGTCGGTTCCGGAAGCATGGACCCGTGAGCGGCAAAGACCGCCCTTGATGCGACCTCAGGTCAGGCGGGGCTACCCGCTGAGTTTAAGCATATCAATAAGCG

>Aevenia_ssp_evenia-127-A

GTAACAAGGTTTCCGTAGGTGAACCTGCGGAAGGATCATTGTTGATGCCTCGACCCAGCTAGACCCGCGAATGTGTTTTACAACTCGGGGTGATCGGGTTGCCTAGGCAGCTCGCCTCCCCGACCCGTTGGGGCTCTTGCCACCCTGTGTGGCTCGGTCCCGACACAACAACAAACCCCGGCGCGGAATGCGCCAAGGAATCACAAACACAAGGCGTGCCCCCTCGACCCGGAAGCGGTGTTCGTCTGGGTGGCGTCGCAAAAAATTGAGTCCAAAATGACTCTCGGCAACGGATATCTCGGCTCTTGCATCGATGAAGAACGTAGCGAAATGCGATACTTGGTGTGAATTGCAGAATCCCGTGAACCATCGAGTCTTTGAACGCAAGTTGCGCCCGAAGCCATTAGGCTAAGGGCACGCCTGCCTGGGTGTCACCAATCGTCGCCCCCAACCTCACTGCCTTGTTGCGTGGAGAAGGGGTGAATGATGGCTTCCCGTGAGCACAGTCTCGCGGTTGGCTGAAAACGTTCTCCGTGCCGGCGTGCAGTGCCGTGACACTTGGTGGTTGAGTTTACCCTCGAGGCCAGTCACGTGTGCTCCCTGTCGGTTCCGGAAGCATGGACCCGTGAGCGGCAAAGACCGCCCTTGATGCGACCTCAGGTCAGGCGGGGCTACCCGCTGAGTTTAAGCATATCAATAAGCG

>Aevenia_ssp_evenia-127-B

GTAACAAGGTTTCCGTAGGTGAACCTGCGGAAGGATCATTGTTGATGCCTCGACCCAGCTAGACCCGCGAATGTGTTTTACTACCCGGGGTGATCGGGATGCCTAGGCAGCTCGCCTCCCCGACCCGTTGGGGCTCTGGCCACCCTGTGTGGCTCGGTCCCGACACAACAACAAACCCCGGCGCGGAATGCGCCAAGGAATAACAAACACAAGGCGTGCCCCCTCGACCCGGAAGCGGTGTTCGTCTGGGTGGCGTCGCAAAAAATTGAGTCCAAAATGACTCTCGGCAACGGATATCTCGGCTCTTGCATCGATGAAGAACGTAGCGAAATGCGATACTTGGTGTGAATTGCAGAATCCCGTGAACCATCGAGTCTTTGAACGCAAGTTGCGCCCGAAGCCATTAGGCTAAGGGCACGCCTGCCTGGGTGTCACCAATCGTCGCCCCCAACCTCACTGCCTTGTTGCGTGGAGAAGGGGTGAATGATGGCTTCCCGTGAGCACAGTCTCGCGGTTGGCTGAAGACGTTCTCCGTGCCGGCGTGCAGTGCCGTGACACTTGGTGGTTGAGTTTACCCTCGAGGCCAGTCACGTGTGCTCCCTGTCGGTTCCGGAAGCATGGACCCGTGAGCGGCAAAGACCGCCCTTGATGCGACCTCAGGTCAGGCGGGGCTACCCGCTGAGTTTAAGCATATCAATAAGCG

>Aevenia_ssp_evenia-132

GTAACAAGGTTTCCGTAGGTGAACCTGCGGAAGGatCATTGTTGATGCCTCGACCCAGCTAGACCCGCGAATGTGTTTTACTACCCGGGGTGATCGGGATGCCTAGGCAGCTCGCCTCCCCGACCCGTTGGGGCTCTGGcCACCCTGTGTGGCTCGGTCCCGACACAACAACAAACCCCGGCGCGgAATGCGCCAAGGAATAACAAACACAAGGCGCGCCCTCTCgACCCGGAAGCGGTGTTCGTCTGGGTGGCGTCGCAAAAAAATTGAGTCCAAAATGACTCTCGGCAACGGATATCTCGGCTCTTGCATCGATGAAGAACGTAGCGAAATGCGATACTTGGTGTGAATTGCAGAATCCCGTGAACCATCgAGTCTTTGAACGCAAGTTGCGCCCGAAGCCATTAGGCTAAGGGCACGCCTGCCTGGGTGTCACCAATCGTCGCCCCCAACCTCACTGCCTTGTTGCGTGGAGAAGGGGTGAATGATGGCTTCCCGTGAGCACAGTCTCGTGGTTGGCTGAAAACGTTCTCCGTGCCGGCGTGCAGCGCCGTGACACTTGGTGGTTGAGTTTACCCTCGAGGCCAGTCACGTGTGCTCCCTGTCgGTTCCGGAAgCATGGACCCgTGAGCGGCAAAgACCGCCCTTGATGCGACCTCAGGTCAGGCGGGGCTACCCGCTgAGTTTAAGCATATCAATAAgCG

>Aevenia_ssp_evenia-141

GTAACAAGGTTTCCGTAGGTGAACCTGCGGAAGGatCATTGTTGATGCCTCGACCCAGCTAGACCCGCGAATGTGTTTTACAACTCGGGGTGATCGGGTTGCCTGGGCAGCTCGCCTCCCCGACCCGTTGGGGCTCTTTCCACCCTGTGTGGCTCGGTCCCGACACAACAACAAACCCCGGCGCGGAATGCGCCAAGGAATCACAAACACAAGGCGTGCCCCCTCGACCCGGAAGCGGTGTTCGTCTGGGTGGCGTCGCAAAAAATTGAGTCCAAAATGACTCTCGGCAACGGATATCTCGGCTCTTGCATCGATGAAGAACGTAGCGAAATGCGATACTTGGTGTGAATTGCAGAATCCCGTGAACCATCGAGTCTTTGAACGCAAGTTGCGCCCGAAGCCATTAGGCTAAGGGCACGCCTGCCTGGGTGTCACCAATCGTCGCCCCCAACCTCACTGCCTTGTTGCGTGGAGAAGGGGTGAATGATGGCTTCCCGTGAGCACAGTCTCGCGGTTGGCTGAAAACGTTCTCCGTGCCGGCGTGCAGTGCCGTGACACTTGGTGGTTGAGTTTACCCTCGAGGCCAGTCACGTGTGCTCCCTGTCGGTTCCGGAAGCATGGACCCGTGAGCGGCAAAgACCGCCCTTGATGCGACCTCAGGTCAgGCGGGGCTACCCGCTGAGTTTAAGCATATCAATAAGCG

>Aevenia_ssp_evenia-143

GTAACAAGGTTTCCGTAGGTGAACCTGCGGAAGGAtCATTGTTGATGCCTCGACCCAGCTAGACCCGCGAATGTGTTTTACTACCCGGGGTGATCGGGCTGCCTAGGCAGCTCGCCTCCCCGACCCGTTGGGGCTCTGGCCACCCTGTGTGGCTCGGTCCCGACACAACAACAAACCCCGGCGCGGAATGCGCCAAGGAATCACAAACACAAGGCGTGCCCCCTCGACCCGGAAGCGGTGTTCGTCTGGGTGGCGTCGCAAAAAAATTGAGTCCAAAATGACTCTCGGCAACGGATATCTCGGCTCTTGCATCGATGAAGAACGTAGCGAAATGCGATACTTGGTGTGAATTGCAGAATCCCGTGAACCATCGAGTCTTTGAACGCAAGTTGCGCCCGAAGCCATTAGGCTAAGGGCACGCCTGCCTGGGTGTCACCAATCGTCGCCCCCAACCTCACTGCCTTGTTGCGTGGAGAAGGGGTGAATGATGGCTTCCCGTGAGCACAGTCTCGCGGTTGGCTGAAAACGTTCTCCGTGCCGGCGTGCAGCGCCGTGACACTTGGTGGTTGAGTTTACCCTCGAGGCCAGTCACGTGTGCTCCCTGTCGGTTCCGGAAGCATGGACCCGTGAGCGGCAAAGACCGCCCTTGATGCGACCTCAGGTCAGGCGGGGCTACCCGCTGAgTTTAAGCATATCAATAAGCG

>Aevenia_ssp_evenia-144

GTAACAAGGTTTCCGTAGGTGAACCTGCGGAAGGAtCATTGTTGATGCCTCGACCCAGCTAGACCCGCGAATGTGTTTTACAACTCGGGGTGATCGGGTTGCCTAGGCAGCTCGCCTCCCCGACCCGTTGGGGCTCTTGCCACCCTGTGTGGCTCGGTCCCGACACAACAACAAACCCCGGCGCGGAATGCGCCAAGGAATCACAAACACAAGGCGTGCCCCCTCGACCCGGAAGCGGTGTTCGTCTGGGTGGCGTCGCAAAAAATTGAGTCCAAAATGACTCTCGGCAACGGATATCTCGGCTCTTGCATCGATGAAGAACGTAGCGAAATGCGATACTTGGTGTGAATTGCAGAATCCCGTGAACCATCGAGTCTTTGAACGCAAGTTGCGCCCGAAGCCATTAGGCTAAGGGCACGCCTGCCTGGGTGTCACCAATCGTCGCCCCCAACCTCACTGCCTTGTTGCGTGGAGAAGGGGTGAATGATGGCTTCCCGTGAGCACAGTCTCGCGGTTGGCTGAAAACGTTCTCCGTGCCGGCGTGCAGTGCCGTGACACTTGGTGGTTGAGTTTACCCTCGAGGCCAGTCACGTGTGCTCCCTGTCGGTTCCGGAAGCATGGACCCGTGAGCGGCAAAGACCGCCCTTGATGCGACCTCAGGTCAGGCGGGGCTACCCGCTGAGTTTAAGCATATCAATAAGCG

>Aevenia_ssp_evenia-153

GTAACAAGGTTTCCGTAGGTGAACCTGCGGAAGGAtCATTGTTGATGCCTCGACCCAGCTAGACCCGCGAATGTGTTTTACAACTCGGGGTGATCGGGTTGCCTAGGCAGCTCGCCTCCCCGACCCGTTGGGGCTCTTGCCACCCTGTGTGGCTCGGTCCCGACACAACAACAAACCCCGGCGCGGAATGCGCCAAGGAATCACAAACACAAGGCGTGCCCCCTCGACCCGGAAGCGGTGTTCGTCTGGGTGGCGTCGCAAAAAATTGAGTCCAAAATGACTCTCGGCAACGGATATCTCGGCTCTTGCATCGATGAAGAACGTAGCGAAATGCGATACTTGGTGTGAATTGCAGAATCCCGTGAACCATCGAGTCTTTGAACGCAAGTTGCGCCCGAAGCCATTAGGCTAAGGGCACGCCTGCCTGGGTGTCACCAATCGTCGCCCCCAACCTCACTGCCTTGTTGCGTGGAGAAGGGGTGAATGATGGCTTCCCGTGAGCACAGTCTCGCGGTTGGCTGAAAACGTTCTCCGTGCCGGCGTGCAGTGCCGTGACACTTGGTGGTTGAGTTTACCCTCGAGGCCAGTCACGTGTGCTCCCTGTCGGTTCCGGAAGCATGGACCCGTGAGCGGCAAAGACCGCCCTTGATGCGACCTCAGGTCAGGCGGGGCTACCCGCtGAGTtTAAGCATATCAATAAGCG

>Aevenia_ssp_evenia-172

GTAACAAGGTTTCCGTAGGTGAACCTGCGGAAGGATCATTGTTGATGCCTCGACCCAGCTAGACCCGCGAATGTGTTTTACAACTCGGGGTGATCGGGTTGCCTAGGCAGCTCGCCTCCCAGACCCGTTGGGGCTCTTGCCACCCTGTGTGGCTCGGTCCCGACACAACAACAAACCCCGGCGCGGAATGCGCCAAGGAATCACAAACACAAGGCGTGCCCCCTCGACCCGGAAGCGGTGTTCGTCTGGGTGGCGTCGCAAAAAATTGAGTCCAAAATGACTCTCGGCAACGGATATCTCGGCTCTTGCATCGATGAAGAACGTAGCGAAATGCGATACTTGGTGTGAATTGCAGAATCCCGTGAACCATCGAGTCTTTGAACGCAAGTTGCGCCCGAAGCCATTAGGCTAAGGGCACGCCTGCCTGGGTGTCACCAATCGTCGCCCCCAACCTCACTGCCTTGTTGCGTGGAGAAGGGGTGAATGATGGCTTCCCGTGAGCACAGTCTCGCGGTTGGCTGAAAACGTTCTCCGTGCCGGCGTGCAGTGCCGTGACACTTGGTGGTTGAGTTTACCCTCGAGGCCAGTCACGTGTGCTCCCTGTCGGTTCCGGAAGCATGGACCCGTGAGCGGCAAAGACCGCCCTTGATGCGACCTCAGGTCAGGCGGGGCTACCCGCTGAGTTTAAGCATATCAATAAGCG

>Aevenia_ssp_evenia-174-A

GTAACAAGGTTTCCGTAGGTGAACCTGCGGAAGGATCATTGTTGATGCCTCGACCCAGCTAGACCCGCGAATGTGTTTTACAACTCGGGGTGATCGGGTTGCCTAGGCAGCTCGCCTCCCCGACCCGTTGGGGCTCTTGCCACCCTGTGTGGCTCGGTCCCGACACAACAACAAACCCCGGCGCGGAATGCGCCAAGGAATCACAAACACAAGGCGTGCCCCCTCGACCCGGAAGCGGTGTTCGTCTGGGTGGCGTCGCAAAAAATTGAGTCCAAAATGACTCTCGGCAACGGATATCTCGGCTCTTGCATCGATGAAGAACGTAGCGAAATGCGATACTTGGTGTGAATTGCAGAATCCCGTGAACCATCGAGTCTTTGAACGCAAGTTGCGCCCGAAGCCATTAGGCTAAGGGCACGCCTGCCTGGGTGTCACCAATCGTCGCCCCCAACCTCACTGCCTTGTTGCGTGGAGAAGGGGTGAATGATGGCTTCCCGTGAGCACAGTCTCGCGGTTGGCTGAAAACGTTCTCCGTGCCGGCGTGCAGTGCCGTGACACTTGGTGGTTGAGTTTACCCTCGAGGCCAGTCACGTGTGCTCCCTGTCGGTTCCGGAAGCATGGACCCGTGAGCGGCAAAGACCGCCCTTGATGCGACCTCAGGTCAGGCGGGGCTACCCGCTGAGTTTAAGCATATCAATAAGCG

>Aevenia_ssp_evenia-174-B

GTAACAAGGTTTCCGTAGGTGAACCTGCGGAAGGATCATTGTTGATGCCTCGACCCAGCTAGACCCGCGAATGTGTTTTACTACCCGGGGTGATCGGGATGCCTAGGCAGCTCGCCTCCCCGACCCGTTGGGGCTCTGGCCACCCTGTGTGGCTCGGTCCCGACACAACAACAAACCCCGGCGCGGAATGCGCCAAGGAATAACAAACACAAGGCGTGCCCCCTCGACCCGGAAGCGGTGTTCGTCTGGGTGGCGTCGCAAAAAATTGAGTCCAAAATGACTCTCGGCAACGGATATCTCGGCTCTTGCATCGATGAAGAACGTAGCGAAATGCGATACTTGGTGTGAATTGCAGAATCCCGTGAACCATCGAGTCTTTGAACGCAAGTTGCGCCCGAAGCCATTAGGCTAAGGGCACGCCTGCCTGGGTGTCACCAATCGTCGCCCCCAACCTCACTGCCTTGTTGCGTGGAGAAGGGGTGAATGATGGCTTCCCGTGAGCACAGTCTCGCGGTTGGCTGAAGACGTTCTCCGTGCCGGCGTGCAGTGCCGTGACACTTGGTGGTTGAGTTTACCCTCGAGGCCAGTCACGTGTGCTCCCTGTCGGTTCCGGAAGCATGGACCCGTGAGCGGCAAAGACCGCCCTTGATGCGACCTCAGGTCAGGCGGGGCTACCCGCTGAGTTTAAGCATATCAATAAGCG

>Aevenia_ssp_evenia-182

GTAACAAGGTTTccGTAGGTGAACCTGCGGAAGGATCATTGTTGATGCCTCGACCCAGCTAGACCCGCGAATGTGTTTTACAACTCGGGGTGATCGGGTTGCCTGGGCAGCTCGCCTCCCCGACCCGTTGGGGCTCTTTCCACCCTGTGTGGCTCGGTCCCGACACAACAACAAACCCCGGCGCGGAATGCGCCAAGGAATCACAAACACAAGGCGTGCCCCCTCGACCCGGAAGCGGTGTTCGTCTGGGTGGCGTCGCAAAAAATTGAGTCCAAAATGACTCTCGGCAACGGATATCTCGGCTCTTGCATCGATGAAGAACGTAGCGAAATGCGATACTTGGTGTGAATTGCAGAATCCCGTGAACCATCGAGTCTTTGAACGCAAGTTGCGCCCGAAGCCATTAGGCTAAGGGCACGCCTGCCTGGGTGTCACCAATCGTCGCCCCCAACCTCACTGCCTTGTTGCGTGGAGAAGGGGTGAATGATGGCTTCCCGTGAGCACAGTCTCGCGGTTGGCTGAAAACGTTCTCCGTGCCGGCGTGCAGTGCCGTGACACTTGGTGGTTGAGTTTACCCTCGAGGCCAGTCACGTGTGCTCCCTGTCGGTTCCGGAAGCATGGACCCGTGAGCGGCAAAGACCGCCCTTGATGCGACCTCAGGTCAGGCGGGGCTACCCGCTGAGTTTAAGCATATCAATAAGCG

>Aevenia_ssp_evenia-202

GTAACAAGGTTTCCGTAGGTGAACCTGCGGAAGGatCATTGTTGATGCCTCGACCCAGCTAGACCCGCGAATGTGTTTTACAACTCGGGGTGATCGGGTTGCCTAGGCAGCTCGCCTCCCCGACCCGTTGGGGCTCTGGCCACCCTGTGTGGCTCGGTCCCGACACAACAACAAACCCCGGCGCGGAATGCGCCAAGGAATCACAAACACAAGGCGTGCCCCCTCGACCCGGAAGCGGTGTTCGTCTGGGTGGCGTCGCAAAAAATTGAGTCCAAAATGACTCTCGGCAACGGATATCTCGGCTCTTGCATCGATGAAGAACGTAGCGAAATGCGATACTTGGTGTGAATTGCAGAATCCCGTGAACCATCGAGTCTTTGAACGCAAGTTGCGCCCGAAGCCATTAGGCTAAGGGCACGCCTGCCTGGGTGTCACCAATCGTCGCCCCCAACCTCACTGCCTTGTTGCGTGGAGAAGGGGTGAATGATGGCTTCCCGTGAGCACAGTCTCGCGGTTGGCTGAAAACGTTCTCCGTGCCGGCGTGCAGTGCCGTGACACTTGGTGGTTGAGTTTACCCTCGAGGCCAGTCACGTGTGCTCCCTGTCGGTTCCGGAAGCATGGACCCGTGAGCGGCAAAGACCGCCCTTGATGCGACCTCAGGTCAGGCGGGGCTACCCGCTGAGTTTAAGCATATCAATAAGCG

>Aevenia_ssp_evenia-350

GTAACAAGGTTTCCgTAGGTGAACCTGCGGAAGGatCATTGTTGATGCCTCGACCCAGCTAGACCCGCGAATGTGTTTTACAACTCGGGGTGATCGGGTTGCCTAGGCAGCTCGCCTCCCCGACCCGTTGGGGCTCTGGCCACCcTGTGTGGCTCGGTCCCGACACAACAACAAACCCCGGCGCGGAATGCGCCAAGGAATCACAAACACAAGGCGTGCCCCCTCGACCCGGAAGCGGTGTTCGTCTGGGTGGYGTCGCAAAAAATTGAGTCCAAAATGACTCTCGGCAACGGATATCTCGGCTCTTGCATCGATGAAGAACGTAGCGAAATGCGATACTTGGTGTGAATTGCAGAATCCCGTGAACCATCGAGTCTTTGAACGCAAGTTGCGCCCGAAGCCATTAGGCTAAGGGCACGCCTGCCTGGGTGTCACCAATCGTCGCCCCCAACCTCACTGCCTTGTTGCGTGGAGAAGGGGTGAATGATGGCTTCCCGTGAGCACAGTCTCGCGGTTGGCTGAAAACGTTCTCCGTGCCGGCGTGCAGTGCCGTGACACTTGGTGGTTGAGTTTACCCTCGAGGCCAGTCACGTGTGCTCCCTGTCGGTTCCGGAAGCATGGACCCGTGAGCGGCAAAGACCGCCCTTGATGCGACCTCAGGTCAGGCGGGGCTACCCGCtgagttaaGCATATCAaTAAGCG

>Aevenia_ssp_serrulata-14-A

GTAACAAGGTTTCCGTAGGTGAACCTGCGGAAGGATCATTGTTGATGCCTCGACCCAGCTAGACCCGCGAATGCGTTTTACCACCCGGGGTGATCGGGCTGCCTAGGCAGCTCGCTTCCCCGACCCGTTGGGGCTCTGGCCACCCTGTGTGGCCCGGTCCCGACACAACAACAAACCCCGGCGCGGAATGCGCCAAGGAATCACAATCACAAGGCGTGCCCCCTCGACCCGGAAGCGGTGTTCGTCAGGGTGGCGTCGCAAAAAATTGAGTCCAAAATGACTCTCGGCAACGGATATCTCGGCTCTTGCATCGATGAAGAACGTAGCGAAATGCGATACTTGGTGTGAATTGCAGAATCCCGTGAACCATCGAGTCTTTGAACGCAAGTTGCGCCCGAAGCCATTAGGCTAAGGGCACGCCTGCCTGGGTGTCACCAATCGTCGCCCCCAACCTCACTGCCTTGTTGCGTGGAGAAGGGGTGAATGATGGCTTCCCGTGAGCACAGTCTTGCGGTTGGCTGAAAACGTTCTCCGTGCCGGCGTGCAGCGTCGTGACACTTGGTGGTTGAGTTTACCCTCGAGGCCAGTCACGTGTGCTCCCTGTCGGTTCCGGAAGCATGGACCCGTGAGCGGCAAAGACCGCCCTTGATGCGACCTCAGGTCAGGCGGGGCTACCCGCTGAGTTTAAGCATATCAATAAGCG

>Aevenia_ssp_serrulata-14-B

GTAACAAGGTTTCCGTAGGTGAACCTGCGGAAGGATCATTGTTGATGCCTCGACCCAGCTAGACCCGCGAATGCGTTTTACCACTCGGGGTGATCGGGCTGCCTAAGGCAGCTCGCTTCCCTGACCCGTTGGGGCTCTGGCCACCCTGTGTGGCCTGGTCCCGACACAACAACAAACCCCGGCGCGGAATGCGCCAAGGAATCACAATCACAAGGCGTGCCCCCTCGACCCGGAAGCGGTGTTCGTCTGGGTGGCGTCGCAAAAAATTGAGTCCAAAATGACTCTCGGCAACGGATATCTCGGCTCTTGCATCGATGAAGAACGTAGCGAAATGCGATACTTGGTGTGAATTGCAGAATCCCGTGAACCATCGAGTCTTTGAACGCAAGTTGCGCCCGAAGCCATTAGGCTAAGGGCACGCCTGCCTGGGTGTCACCAATCGTCGCCCCCAACCTCACTGCCTTGTTGCGTGGAGAAGGGGTGAATGATGGCTTCCCGTGAGCACAGTCTTGCGGTTGGCTGAAAATGTTCTCCGTGCCGGCGTGCAGCGTCGTGACACTTGGTGGTTGAGTTTACCCTCGAGGCCAGTCACGTGTGCTCCCTGTTGGTTCCGGAAGCATGGACCCGTGAGCGGCAAAGACCGCCCTTGATGCGACCTCAGGTCAGGCGGGGCTACCCGCTGAGTTTAAGCATATCAATAAGCG

>Aevenia_ssp_serrulata-65

GTAACAAGGTTTCCGTAGGTGAACCTGCGGAAGGATCATTGTTGATGCCTCGACCCAGCTAGACCCGCGAATGCGTTTTACCACCCGGGGTGATCGGGCTGCCTAGGCAGCTCGCTTCCCCGACCCGTTGGGGCTCTGGCCACCCTGTGTGGCCCGGTCCCGACACAACAACAAACCCCGGCGCGGAATGCGCCAAGGAATCACAATCACAAGGCGTGCCCCCTCGACCCGGAAGCGGTGTTCGTCAGGGTGGCGTCGCAAAAAATTGAGTCCAAAATGACTCTCGGCAACGGATATCTCGGCTCTTGCATCGATGAAGAACGTAGCGAAATGCGATACTTGGTGTGAATTGCAGAATCCCGTGAACCATCGAGTCTTTGAACGCAAGTTGCGCCCGAAGCCATTAGGCTAAGGGCACGCCTGCCTGGGTGTCACCAATCGTCGCCCCCAACCTCACTGCCTTGTTGCGTGGAGAAGGGGTGAATGATGGCTTCCCGTGAGCACAGTCTTGCGGTTGGCTGAAAACGTTCTCCGTGCCGGCGTGCAGCGTCGTGACACTTGGTGGTTGAGTTTACCCTCGAGGCCAGTCACGTGTGCTCCCTGTCGGTTCCGGAAGCATGGACCCGTGAGCGGCAAAGACCGCCCTTGATGCGACCTCAGGTCAGGCGGGGCTACCCGCTGAGTTTAAGCATATCAATAAGCG

>Aevenia_ssp_serrulata-66

GTAACAAGGTTTCCGTAGGTGAACCTGCGGAAGGATCATTGTTGATGCCTCGACCCAGCTAGACCCGCGAATGCGTTTTACCACCCGGGGTGATCGGGCTGCCTAGGCAGCTCGCTTCCCCGACCCGTTGGGGCTCTGGCCACCCTGTGTGGCCCGGTCCCGACACAACAACAAACCCCGGCGCGGAATGCGCCAAGGAATCACAATCACAAGGCGTGCCCCCTCGACCCGGAAGCGGTGTTCGTCAGGGTGGCGTCGCAAAAAATTGAGTCCAAAATGACTCTCGGCAACGGATATCTCGGCTCTTGCATCGATGAAGAACGTAGCGAAATGCGATACTTGGTGTGAATTGCAGAATCCCGTGAACCATCGAGTCTTTGAACGCAAGTTGCGCCCGAAGCCATTAGGCTAAGGGCACGCCTGCCTGGGTGTCACCAATCGTCGCCCCCAACCTCACTGCCTTGTTGCGTGGAGAAGGGGTGAATGATGGCTTCCCGTGAGCACAGTCTTGCGGTTGGCTGAAAACGTTCTCCGTGCCGGCGTGCAGCGTCGTGACACTTGGTGGTTGAGTTTACCCTCGAGGCCAGTCACGTGTGCTCCCTGTCGGTTCCGGAAGCATGGACCCGTGAGCGGCAAAGACCGCCCTTGATGCGACCTCAGGTCAGGCGGGGCTACCCGCTGAGTTTAAGCATATCAATAAGCG

>Aevenia_ssp_serrulata-71-A

GTAACAAGGTTTCCGTAGGTGAACCTGCGGAAGGATCATTGTTGATGCCTCGACCCAGCTAGACCCGCGAATGCGTTTTACCACCCGGGGTGATCGGGCTGCCTAGGCAGCTCGCTTCCCCGACCCGTTGGGGCTCTGGCCACCCTGTGTGGCCCGGTCCCGACACAACAACAAACCCCGGCGCGGAATGCGCCAAGGAATCACAATCACAAGGCGTGCCCCCTCGACCCGGAAGCGGTGTTCGTCAGGGTGGCGTCGCAAAAAATTGAGTCCAAAATGACTCTCGGCAACGGATATCTCGGCTCTTGCATCGATGAAGAACGTAGCGAAATGCGATACTTGGTGTGAATTGCAGAATCCCGTGAACCATCGAGTCTTTGAACGCAAGTTGCGCCCGAAGCCATTAGGCTAAGGGCACGCCTGCCTGGGTGTCACCAATCGTCGCCCCCAACCTCACTGCCTTGTTGCGTGGAGAAGGGGTGAATGATGGCTTCCCGTGAGCACAGTCTTGCGGTTGGCTGAAAACGTTCTCCGTGCCGGCGTGCAGCGTCGTGACACTTGGTGGTTGAGTTTACCCTCGAGGCCAGTCACGTGTGCTCCCTGTCGGTTCCGGAAGCATGGACCCGTGAGCGGCAAAGACCGCCCTTGATGCGACCTCAGGTCAGGCGGGGCTACCCGCTGAGTTTAAGCATATCAATAAGCG

>Aevenia_ssp_serrulata-71-B

GTAACAAGGTTTCCGTAGGTGAACCTGCGGAAGGATCATTGTTGATGCCTCGACCCAGCTAGACCCGCGAATGCGTTTTACCACTCGGGGTGATCGGGCTGCCTAAGGCAGCTCGCTTCCCTGACCCGTTGGGGCTCTGGCCACCCTGTGTGGCCTGGTCCCGACACAACAACAAACCCCGGCGCGGAATGCGCCAAGGAATCACAATCACAAGGCGTGCCCCCTCGACCCGGAAGCGGTGTTCGTCTGGGTGGCGTCGCAAAAAATTGAGTCCAAAATGACTCTCGGCAACGGATATCTCGGCTCTTGCATCGATGAAGAACGTAGCGAAATGCGATACTTGGTGTGAATTGCAGAATCCCGTGAACCATCGAGTCTTTGAACGCAAGTTGCGCCCGAAGCCATTAGGCTAAGGGCACGCCTGCCTGGGTGTCACCAATCGTCGCCCCCAACCTCACTGCCTTGTTGCGTGGAGAAGGGGTGAATGATGGCTTCCCGTGAGCACAGTCTTGCGGTTGGCTGAAAATGTTCTCCGTGCCGGCGTGCAGCGTCGTGACACTTGGTGGTTGAGTTTACCCTCGAGGCCAGTCACGTGTGCTCCCTGTTGGTTCCGGAAGCATGGACCCGTGAGCGGCAAAGACCGCCCTTGATGCGACCTCAGGTCAGGCGGGGCTACCCGCTGAGTTTAAGCATATCAATAAGCG

>Aevenia_ssp_serrulata-72

GTAACAAGGTTTCCGTAGGTGAACCTGCGGAAGGATCATTGTTGATGCCTCGACCCAGCTAGACCCGCGAATGCGTTTTACCACTCGGGGTGATCGGGCTGCCTAAGGCAGCTCGCTTCCCTGACCCGTTGGGGCTCTGGCCACCCTGTGTGGCCTGGTCCCGACACAACAACAAACCCCGGCGCGGAATGCGCCAAGGAATCACAATCACAAGGCGTGCCCCCTCGACCCGGAAGCGGTGTTCGTCTGGGTGGCGTCGCAAAAAATTGAGTCCAAAATGACTCTCGGCAACGGATATCTCGGCTCTTGCATCGATGAAGAACGTAGCGAAATGCGATACTTGGTGTGAATTGCAGAATCCCGTGAACCATCGAGTCTTTGAACGCAAGTTGCGCCCGAAGCCATTAGGCTAAGGGCACGCCTGCCTGGGTGTCACCAATCGTCGCCCCCAACCTCACTGCCTTGTTGCGTGGAGAAGGGGTGAATGATGGCTTCCCGTGAGCACAGTCTTGCGGTTGGCTGAAAATGTTCTCCGTGCCGGCGTGCAGCGTCGTGACACTTGGTGGTTGAGTTTACCCTCGAGGCCAGTCACGTGTGCTCCCTGTTGGTTCCGGAAGCATGGACCCGTGAGCGGCAAAGACCGCCCTTGATGCGACCTCAGGTCAGGCGGGGCTACCCGCTGAGTTTAAGCATATCAATAAGCG

>Aevenia_ssp_serrulata-74

GTAACAAGGTTTCCGTAGGTGAACCTGCGGAAGGATCATTGTTGATGCCTCGACCCAGCTAGACCCGCGAATGCGTTTTACCACTCGGGGTGATCGGGCTGCCTAAGGCAGCTCGCTTCCCTGACCCGTTGGGGCTCTGGCCACCCTGTGTGGCCTGGTCCCGACACAACAACAAACCCCGGCGCGGAATGCGCCAAGGAATCACAATCACAAGGCGTGCCCCCTCGACCCGGAAGCGGTGTTCGTCTGGGTGGCGTCGCAAAAAATTGAGTCCAAAATGACTCTCGGCAACGGATATCTCGGCTCTTGCATCGATGAAGAACGTAGCGAAATGCGATACTTGGTGTGAATTGCAGAATCCCGTGAACCATCGAGTCTTTGAACGCAAGTTGCGCCCGAAGCCATTAGGCTAAGGGCACGCCTGCCTGGGTGTCACCAATCGTCGCCCCCAACCTCACTGCCTTGTTGCGTGGAGAAGGGGTGAATGATGGCTTCCCGTGAGCACAGTCTTGCGGTTGGCTGAAAATGTTCTCCGTGCCGGCGTGCAGCGTCGTGACACTTGGTGGTTGAGTTTACCCTCGAGGCCAGTCACGTGTGCTCCCTGTTGGTTCCGGAAGCATGGACCCGTGAGCGGCAAAGACCGCCCTTGATGCGACCTCAGGTCAGGCGGGGCTACCCGCTGAGTTTAAGCATATCAATAAGCG

>Aevenia_ssp_serrulata-258

GTAACAAGGTTTCCGTAGGTGAACCTGCGGAAGGaTCATTGTTGATGCCTCGACCCAGCTAGACCCGCGAATGCGTTTTACCACTCGGGGTGATCGGGCTGCCTAAGGCAGCTCGCTTCCCTGACCCGTTGGGGCTCTGGCCACCCTGTGTGGCCTGGTCCCGACACAACAACAAACCCCGGCGCGGAATGCGCCAAGGAATCACAATCACAAGGCGTGCCCCCTCGACCCGGAAGCGGTGTTCGTCTGGGTGGCGTCGCAAAAAATTGAGTCCAAAATGACTCTCGGCAACGGATATCTCGGCTCTTGCATCGATgAAgAACGTAGCGAAATGCGATACTTGGTGTGAATTGCAgAATCCCGTGAACCATCGAGTCTTTGAACGCAAGTTGCGCCCGAAGCCATTAGGCTAAGGGCACGCCTGCCTGGGTGTCACCAATCGTCGCCCCCAACCTCACTGCCTTGTTGCGTGGAGAAGGGGTGAATGATGGCTTCCCGTGAGCACAGTCTTGCGGTTGGCTGAAAATGTTCTCCGTGCCGGCGTGCAGCGTCgTGACACTTGGTGGTTGAGTTTACCCTCGAGGCCAGTCACGTGTGCTCCCTGTTGGTTCCGGAAGCATGGACCCGTGAGCGGCAAAGACCGCCCTTGATGCGACCTCAGGTCAGGCGGGGCTACCCGCTGAGTTTAAGCATATCAaTAAGCG

>Aevenia_ssp_serrulata-323-A

GTAACAAGGTTTCCGTAGGTGAACCTGCGGAAGGATCATTGTTGATGCCTCGACCCAGCTAGACCCGCGAATGCGTTTTACCACCCGGGGTGATCGGGCTGCCTAGGCAGCTCGCTTCCCCGACCCGTTGGGGCTCTGGCCACCCTGTGTGGCCCGGTCCCGACACAACAACAAACCCCGGCGCGGAATGCGCCAAGGAATCACAATCACAAGGCGTGCCCCCTCGACCCGGAAGCGGTGTTCGTCAGGGTGGCGTCGCAAAAAATTGAGTCCAAAATGACTCTCGGCAACGGATATCTCGGCTCTTGCATCGATGAAGAACGTAGCGAAATGCGATACTTGGTGTGAATTGCAGAATCCCGTGAACCATCGAGTCTTTGAACGCAAGTTGCGCCCGAAGCCATTAGGCTAAGGGCACGCCTGCCTGGGTGTCACCAATCGTCGCCCCCAACCTCACTGCCTTGTTGCGTGGAGAAGGGGTGAATGATGGCTTCCCGTGAGCACAGTCTTGCGGTTGGCTGAAAACGTTCTCCGTGCCGGCGTGCAGCGTCGTGACACTTGGTGGTTGAGTTTACCCTCGAGGCCAGTCACGTGTGCTCCCTGTCGGTTCCGGAAGCATGGACCCGTGAGCGGCAAAGACCGCCCTTGATGCGACCTCAGGTCAGGCGGGGCTACCCGCTGAGTTTAAGCATATCAATAAGCG

>Aevenia_ssp_serrulata-323-B

GTAACAAGGTTTCCGTAGGTGAACCTGCGGAAGGATCATTGTTGATGCCTCGACCCAGCTAGACCCGCGAATGCGTTTTACCACTCGGGGTGATCGGGCTGCCTAAGGCAGCTCGCTTCCCTGACCCGTTGGGGCTCTGGCCACCCTGTGTGGCCTGGTCCCGACACAACAACAAACCCCGGCGCGGAATGCGCCAAGGAATCACAATCACAAGGCGTGCCCCCTCGACCCGGAAGCGGTGTTCGTCTGGGTGGCGTCGCAAAAAATTGAGTCCAAAATGACTCTCGGCAACGGATATCTCGGCTCTTGCATCGATGAAGAACGTAGCGAAATGCGATACTTGGTGTGAATTGCAGAATCCCGTGAACCATCGAGTCTTTGAACGCAAGTTGCGCCCGAAGCCATTAGGCTAAGGGCACGCCTGCCTGGGTGTCACCAATCGTCGCCCCCAACCTCACTGCCTTGTTGCGTGGAGAAGGGGTGAATGATGGCTTCCCGTGAGCACAGTCTTGCGGTTGGCTGAAAATGTTCTCCGTGCCGGCGTGCAGCGTCGTGACACTTGGTGGTTGAGTTTACCCTCGAGGCCAGTCACGTGTGCTCCCTGTTGGTTCCGGAAGCATGGACCCGTGAGCGGCAAAGACCGCCCTTGATGCGACCTCAGGTCAGGCGGGGCTACCCGCTGAGTTTAAGCATATCAATAAGCG

>Aevenia_ssp_serrulata-326-A

GTAACAAGGTTTCCGTAGGTGAACCTGCGGAAGGATCATTGTTGATGCCTCGACCCAGCTAGACCCGCGAATGCGTTTTACCACCCGGGGTGATCGGGCTGCCTAGGCAGCTCGCTTCCCCGACCCGTTGGGGCTCTGGCCACCCTGTGTGGCCCGGTCCCGACACAACAACAAACCCCGGCGCGGAATGCGCCAAGGAATCACAATCACAAGGCGTGCCCCCTCGACCCGGAAGCGGTGTTCGTCAGGGTGGCGTCGCAAAAAATTGAGTCCAAAATGACTCTCGGCAACGGATATCTCGGCTCTTGCATCGATGAAGAACGTAGCGAAATGCGATACTTGGTGTGAATTGCAGAATCCCGTGAACCATCGAGTCTTTGAACGCAAGTTGCGCCCGAAGCCATTAGGCTAAGGGCACGCCTGCCTGGGTGTCACCAATCGTCGCCCCCAACCTCACTGCCTTGTTGCGTGGAGAAGGGGTGAATGATGGCTTCCCGTGAGCACAGTCTTGCGGTTGGCTGAAAACGTTCTCCGTGCCGGCGTGCAGCGTCGTGACACTTGGTGGTTGAGTTTACCCTCGAGGCCAGTCACGTGTGCTCCCTGTCGGTTCCGGAAGCATGGACCCGTGAGCGGCAAAGACCGCCCTTGATGCGACCTCAGGTCAGGCGGGGCTACCCGCTGAGTTTAAGCATATCAATAAGCG

>Aevenia_ssp_serrulata-326-B

GTAACAAGGTTTCCGTAGGTGAACCTGCGGAAGGATCATTGTTGATGCCTCGACCCAGCTAGACCCGCGAATGCGTTTTACCACTCGGGGTGATCGGGCTGCCTAAGGCAGCTCGCTTCCCTGACCCGTTGGGGCTCTGGCCACCCTGTGTGGCCTGGTCCCGACACAACAACAAACCCCGGCGCGGAATGCGCCAAGGAATCACAATCACAAGGCGTGCCCCCTCGACCCGGAAGCGGTGTTCGTCTGGGTGGCGTCGCAAAAAATTGAGTCCAAAATGACTCTCGGCAACGGATATCTCGGCTCTTGCATCGATGAAGAACGTAGCGAAATGCGATACTTGGTGTGAATTGCAGAATCCCGTGAACCATCGAGTCTTTGAACGCAAGTTGCGCCCGAAGCCATTAGGCTAAGGGCACGCCTGCCTGGGTGTCACCAATCGTCGCCCCCAACCTCACTGCCTTGTTGCGTGGAGAAGGGGTGAATGATGGCTTCCCGTGAGCACAGTCTTGCGGTTGGCTGAAAATGTTCTCCGTGCCGGCGTGCAGCGTCGTGACACTTGGTGGTTGAGTTTACCCTCGAGGCCAGTCACGTGTGCTCCCTGTTGGTTCCGGAAGCATGGACCCGTGAGCGGCAAAGACCGCCCTTGATGCGACCTCAGGTCAGGCGGGGCTACCCGCTGAGTTTAAGCATATCAATAAGCG

>Afilosa-64

GTAACAAGGTTTCCGTAGGTGAACCTGCGGAAGGATCATTGTTGATGCCTCAATCCAGCTAGACCCGCGAATTCGTTTTACTACCCGGGGCGATCGAGCTACCTCGGCAGCTCGCCTCCCCGAAACGTTGGGGCGTTGCCGCCCCGTGTGGCCTCGTCCCAGCGCAACAACAAACCCCGGCGCGGAATGCGCCAAGGAATTCACAACCGTAAGGCGCGCCCCATCGACCTGGCAACGGTGTTCGTTTGGGTGGCGTTGCGAAAATCGAGTCTAAAACGACTCTCGGCAACGGATATCTCGGCTCCTGCATCGATGAAGAACGTAGCGAAATGCGATACTTGGTGTGAATTGCAGAATCCCGTGAACCATCGAGTCTTTGAACGCAAGTTGCGCCTGAAGCCATTAGGCTAAGGGCATGCCTGCCTGGGTGTCACTAATCGTCGCCCCAACCCAGTGCCTTCGGGCGTGGAGGGGGGTGAATGCTGGCTTCCTGTGAGCATCGTCTCGCTGTTGGCTGAAAACGTTCTCTGTGCCGGCGTGCAGCGCTGTGATACATGGTGGTTGAGTCTATTCTCGATGCCTGTCACAGGTGCCCCCTGTCGGTTCTGGAAACATTGACCCTTGATGCGAGCGACCTCAGGTCAGGCGGGGCTACCCGCTGAGTTTAAGCATATCAATAAGCG

>Afilosa-117

GTAACAAGGTTTCCGTAGGTGAACCTGCGGAAGGATCATTGTTGATGCCTCAATCCAGCTAGACCCGCGAATTCGTTTTACTACCCGGGGCGATCGAGCTACCTCGGCAGCTCGCCTCCCCGAAACGTTGGGGCGTTGCCGCCCCGTGTGGCCTCGTCCCAGCGCAACAACAAACCCCGGCGCGGAATGCGCCAAGGAATTCACAACCGTAAGGCGCGCCCCATCGACCTGGCAACGGTGTTCGTTTGGGTGGCGTTGCGAAAATCGAGTCTAAAACGACTCTCGGCAACGGATATCTCGGCTCCTGCATCGATGAAGAACGTAGCGAAATGCGATACTTGGTGTGAATTGCAGAATCCCGTGAACCATCGAGTCTTTGAACGCAAGTTGCGCCTGAAGCCATTAGGCTAAGGGCATGCCTGCCTGGGTGTCACTAATCGTCGCCCCAACCCAGTGCCTTCGGGCGTGGAGGGGGGTGAATGCTGGCTTCCTGTGAGCATCGTCTCGCTGTTGGCTGAAAACGTTCTCTGTGCCGGCGTGCAGCGCTGTGATACATGGTGGTTGAGTCTATTCTCGATGCCTGTCACAGGTGCCCCCTGTCGGTTCTGGAAACATTGACCCTTGATGCGAGCGACCTCAGGTCAGGCGGGGCTACCCGCTGAGTTTAAGCATATCAATAAGCG

>Aindica_4x_20

GTAACAAGGTTTCCGTAGGTGAACCTGCGGAAGGATCATTGTTGATGCCTCGACCCAGCTAGACCCGCGAATGTGTTTTACAACTCGGGGTGATCGGGTTGCCTAGGCAGCTCGCCTCCCCGACCCGTTGGGTCTCTGGCCACCCTGTGTGGCTCGGTCCCGACACAACAACAAACCCCGGCGCGGAATGCGCCAAGGAATCACAAACACAAGGCGTGCCCCCTCGACCCGGAAGCGGTGTTCGTCTGGGTGGCGTCGCAAAAAATTGAGTCCAAAATGACTCTCGGCAACGGATATCTCGGCTCTTGCATCGATGAAGAACGTAGCGAAATGCGATACTTGGTGTGAATTGCAGAATCCCGTGAACCATCGAGTCTTTGAACGCAAGTTGCGCCCGAAGCCATTAGGCTAAGGGCACGCCTGCCTGGGTGTCACCAATCGTTGCCCCCAACCTCACTGCCTTGTTGCGTGGAGAAGGGGTGAATGATGGCTTCCCGTGAGCACAGTCTCGCGGTTGGCTGAAAACGTTCTCCGTGCCGGCGTGCAGTGCCGTGACACTTGGTGGTTGAGTTTACCCTCGAGGCCAGTCACGTGTGCTCCCTGTCGGTTCCGGAAGCATGGACCCGTGAGCGGCAAAGACCGCCCTTGATGCGACCTCAGGTCAGGCGGGGCTACCCGCTGAGTTTAAGCATATCAATAAGCG

>Aindica_4x_94

GTAACAAGGTTTCCGTAGGTGAACCTGCGGAAGGATCATTGTTGATGCCTCGACCCAGCTAGACCCGCGAATGTGTTTTACAACTCGGGGTGATCGGGTTGCCTAGGCAGCTCGCCTCCCCGACCCGTTGGGTCTCTGGCCACCCTGTGTGGCTCGGTCCCGACACAACAACAAACCCCGGCGCGGAATGCGCCAAGGAATCACAAACACAAGGCGTGCCCCCTCGACCCGGAAGCGGTGTTCGTCTGGGTGGCGTCGCAAAAAATTGAGTCCAAAATGACTCTCGGCAACGGATATCTCGGCTCTTGCATCGATGAAGAACGTAGCGAAATGCGATACTTGGTGTGAATTGCAGAATCCCGTGAACCATCGAGTCTTTGAACGCAAGTTGCGCCCGAAGCCATTAGGCTAAGGGCACGCCTGCCTGGGTGTCACCAATCGTTGCCCCCAACCTCACTGCCTTGTTGCGTGGAGAAGGGGTGAATGATGGCTTCCCGTGAGCACAGTCTCGCGGTTGGCTGAAAACGTTCTCCGTGCCGGCGTGCAGTGCCGTGACACTTGGTGGTTGAGTTTACCCTCGAGGCCAGTCACGTGTGCTCCCTGTCGGTTCCGGAAGCATGGACCCGTGAGCGGCAAAGACCGCCCTTGATGCGACCTCAGGTCAGGCGGGGCTACCCGCTGAGTTTAAGCATATCAATAAGCG

>Aindica_4x_99

GTAACAAGGTTTCCGTAGGTGAACCTGCGGAAGGATCATTGTTGATGCCTCGACCCAGCTAGACCCGCGAATGTGTTTTACAACTCGGGGTGATCGGGTTGCCTAGGCAGCTCGCCTCCCCGACCCGTTGGGTCTCTGGCCACCCTGTGTGGCTCGGTCCCGACACAACAACAAACCCCGGCGCGGAATGCGCCAAGGAATCACAAACACAAGGCGTGCCCCCTCGACCCGGAAGCGGTGTTCGTCTGGGTGGCGTCGCAAAAAATTGAGTCCAAAATGACTCTCGGCAACGGATATCTCGGCTCTTGCATCGATGAAGAACGTAGCGAAATGCGATACTTGGTGTGAATTGCAGAATCCCGTGAACCATCGAGTCTTTGAACGCAAGTTGCGCCCGAAGCCATTAGGCTAAGGGCACGCCTGCCTGGGTGTCACCAATCGTTGCCCCCAACCTCACTGCCTTGTTGCGTGGAGAAGGGGTGAATGATGGCTTCCCGTGAGCACAGTCTCGCGGTTGGCTGAAAACGTTCTCCGTGCCGGCGTGCAGTGCCGTGACACTTGGTGGTTGAGTTTACCCTCGAGGCCAGTCACGTGTGCTCCCTGTCGGTTCCGGAAGCATGGACCCGTGAGCGGCAAAGACCGCCCTTGATGCGACCTCAGGTCAGGCGGGGCTACCCGCTGAGTTTAAGCATATCAATAAGCG

>Aindica_4x_103

GTAACAAGGTTTCCGTAGGTGAACCTGCGGAAGGATCATTGTTGATGCCTCGACCCAGCTAGACCCGCGAATGTGTTTTACAACTCGGGGTGATCGGGTTGCCTAGGCAGCTCGCCTCCCCGACCCGTTGGGTCTCTGGCCACCCTGTGTGGCTCGGTCCCGACACAACAACAAACCCCGGCGCGGAATGCGCCAAGGAATCACAAACACAAGGCGTGCCCCCTCGACCCGGAAGCGGTGTTCGTCTGGGTGGCGTCGCAAAAAATTGAGTCCAAAATGACTCTCGGCAACGGATATCTCGGCTCTTGCATCGATGAAGAACGTAGCGAAATGCGATACTTGGTGTGAATTGCAGAATCCCGTGAACCATCGAGTCTTTGAACGCAAGTTGCGCCCGAAGCCATTAGGCTAAGGGCACGCCTGCCTGGGTGTCACCAATCGTTGCCCCCAACCTCACTGCCTTGTTGCGTGGAGAAGGGGTGAATGATGGCTTCCCGTGAGCACAGTCTCGCGGTTGGCTGAAAACGTTCTCCGTGCCGGCGTGCAGTGCCGTGACACTTGGTGGTTGAGTTTACCCTCGAGGCCAGTCACGTGTGCTCCCTGTCGGTTCCGGAAGCATGGACCCGTGAGCGGCAAAGACCGCCCTTGATGCGACCTCAGGTCAGGCGGGGCTACCCGCTGAGTTTAAGCATATCAATAAGCG

>Aindica_4x_121

GTAACAAGGTTTCCGTAGGTGAACCTGCGGAAGGatCATTGTTGATGCCTCGACCCAGCTAGACCCGCGAATGTGTTTTACAACTCGGGGTGATCGGGTTGCCTAGGCAGCTCGCCTCCCCGACCCGTTGGGTCTCTGGCCACCCTGTGTGGCTCGGTCCCGACACAACAACAAACCCCGGCGCGGAATGCGCCAAGGAATCACAAACACAAGGCGTGCCCCCTCGACCCGGAAGCGGTGTTCGTCTGGGTGGCGTCGCAAAAAATTGAGTCCAAAATGACTCTCGGCAACGGATATCTCGGCTCTTGCATCGATGAAGAACGTAGCGAAATGCGATACTTGGTGTGAATTGCAGAATCCCGTGAACCATCGAGTCTTTGAACGCAAGTTGCGCCCGAAGCCATTAGGCTAAGGGCACGCCTGCCTGGGTGTCACCAATCGTTGCCCCCAACCTCACTGCCTTGTTGCGTGGAGAAGGGGTGAATGATGGCTTCCCGTGAGCACAGTCTCGCGGTTGGCTGAAAACGTTCTCCGTGCCGGCGTGCAGTGCCGTGACACTTGGTGGTTGAGTTTACCCTCGAGGCCAGTCACGTGTGCTCCCTGTCGGTTCCGGAAGCATGGACCCGTGAGCGGCAAAGACCGCCCTTGATGCGACCTCAGGTCAGGCGGGGCTACCCGCTGAGTTTAAGCATATCAATAAGCG

>Aindica_4x_152

GTAACAAGGTTTCCGTAGGTGAACCTGCGGAAGGAtCATTGTTGATGCCTCGACCCAGCTAGACCCGCGAATGTGTTTTACAACTCGGGGTGATCGGGTTGCCTAGGCAGCTCGCCTCCCCGACCCGTTGGGTCTCTGGCCAACCCTGTGTGGCTCGGTCCCGACACAACAACAAACCCCGGCGCGGAATGCGCCAAGGAATCACAAACACAAGGCGTGCCCCCTCGACCCGGAAGCGGTGTTCGTCTGGGTGGCGTCGCAAAAAATTGAGTCCAAAATGACTCTCGGCAACGGATATCTCGGCTCTTGCATCGATGAAGAACGTAGCGAAATGCGATACTTGGTGTGAATTGCAGAATCCCGTGAACCATCGAGTCTTTGAACGCAAGTTGCGCCCGAAGCCATTAGGCTAAGGGCACGCCTGCCTGGGTGTCACCAATCGTTGCCCCCAACCTCACTGCCTTGTTGCGTGGAGAAGGGGTGAATGATGGCTTCCCGTGAGCACAGTCTCGCGGTTGGCTGAAAACGTTCTCCGTGCCGGCGTGCAGTGCCGTGACACTTGGTGGTTGAGTTTACCCTCGAGGCCAGTCACGTGTGCTCCCTGTCGGTTCCGGAAGCATGGACCCGTGAGCGGCAAAGACCGCCCTTGATGCGACCTCAGGTCAGGCGGGGCTACCCGCTGAGTTTAAGCATATCAATAAGCG

>Aindica_4x_157

GTAACAAGGTTTCCGTAGGTGAACCTGCGGAAGGatCATTGTTGATGCCTCGACCCAGCTAGACCCGCGAATGTGTTTTACAACTCGGGGTGATCGGGTTGCCTAGGCAGCTCGCCTCCCCGACCCGTTGGGTCTCTGGCCACCCTGTGTGGCTCGGTCCCGACACAACAACAAACCCCGGCGCGGAATGCGCCAAGGAATCACAAACACAAGGCGTGCCCCCTCGACCCGGAAGCGGTGTTCGTCTGGGTGGCGTCGCAAAAAATTGAGTCCAAAATGACTCTCGGCAACGGATATCTCGGCTCTTGCATCGATGAAGAACGTAGCGAAATGCGATACTTGGTGTGAATTGCAGAATCCCGTGAACCATCGAGTCTTTGAACGCAAGTTGCGCCCGAAGCCATTAGGCTAAGGGCACGCCTGCCTGGGTGTCACCAATCGTTGCCCCCAACCTCACTGCCTTGTTGCGTGGAGAAGGGGTGAATGATGGCTTCCCGTGAGCACAGTCTCGCGGTTGGCTGAAAACGTTCTCCGTGCCGGCGTGCAGTGCCGTGACACTTGGTGGTTGAGTTTACCCTCGAGGCCAGTCACGTGTGCTCCCTGTCGGTTCCGGAAGCATGGACCCGTGAGCGGCAAAGACCGCCCTTGATGCGACCTCAGGTCAGGCGGGGCTACCCGCTGAGTTTAAGCATATCAATAAGCG

>Aindica_4x_173

GTAACAAGGTTTCCGTAGGTGaaCCTGCGGAAGGatCATTGTTGATGCCTCGACCCAGCTAGACCCGCGAATGTGTTTTACAACTCGGGGTGATCGGGTTGCCTAGGCAGCTCGCCTCCCCGACCCGTTGGATCTCTGGCCACCCTGTGTGGCTCGGTCCCGACACAACAACAAACCCCGGCGCGGAATGCGCCAAGGAATCACAATCACAAGGCGTGCCCCCTCGACCCGGAAGCGGTGTTCGTCTGGGTGGCGTCGCAAAAAATTGAGTCCAAAATGACTCTCGGCAACGGATATCTCGGCTCTTGCATCGATGAAGAACGTAGCGAAATGCGATACTTGGTGTGAATTGCAGAATCCCGTGAACCATCGAGTCTTTGAACGCAAGTTGCGCCCGAAGCCATTAGGCTAAGGGCACGCCTGCCTGGGTGTCACCAATCGTCGCCCCCAACCTCACTGCCTTGTTGCGTGGAGAAGGGGTGAATGATGGCTTCCCGTGAGCACAGTCTCGCGGTTGGCTGAAAATGTTCTCCGTGCCGGCGTGCAGTGCCGTGACACTTGGTGGTTGAGTTTACCCTCGAGGCCAGTCACGTGTGCTCCCTGTCGGTTCCGGAAGCATGGACCCGTGAGCGGCAAAGACCGCCCTTGATGCGACCTCAGGTCAGGCGGGGCTACCCGCTGAGTTTAAGCATATCAATAAGCG

>Aindica_4x_189

GTAACAAGGTTTCCGTAGGTGAACCTGCGGaaGGatCATTGTTGATGCCTCGACCCAGCTAGACCCGCGAATGTGTTTTACAACTCGGGGTGGTCGGGTTGCCTAGGCAGCTCGCCTCCCCGACCCGTTGGATCTCTGGCCACCCTGTGTGGCTCGGTCCCGACACAACAACAAACCCCGGCGCGGAATGCGCCAAGGAATCACAATCACAAGGCGTGCCCCCTCGACCCGGAAGCGGTGTTCGTCTGGGTGGCGTCGCAAAAAATTGAGTCCAAAATGACTCTCGGCAACGGATATCTCGGCTCTTGCATCGATGAAGAACGTAGCGAAATGCGATACTTGGTGTGAATTGCAGAATCCCGTGAACCATCGAGTCTTTGAACGCAAGTTGCGCCCGAAGCCATTAGGCTAAGGGCACGCCTGCCTGGGTGTCACCAATCGTCGCCCCCAACCTCACTGCCTTGTTGCGTGGAGAAGGGGTGAATGATGGCTTCCCGTGAGCACAGTCTCGCGGTTGGCTGAAAACGTTCTCCGTGCCGGCGTGCAGTGCCGTGACACTTGGTGGTTGAGTTTAWCCTCGAGGCCAGTCACGTGTGCTCCCTGTCGGTTCCGGAAGCATGGACCCGTGAGCGGCAAAGACCGCCCTTGATGCGACCTCAGGTCAGGCGGGGCTACCCGCTGAGTTTAAGCATATCAATAAGCG

>Aindica_4x_201

GTAACAAGGTTTCCGTAgGtgaACCTGCGGAAGGatcATTGTTGATGCCTCGACCCAGCTAGACCCGCGAATGTGTTTTACAACTCGGGGTGATCGGGTTGCCTAGGTAGCTCGCCTCCCCGACCCGTTGGGTCTCTGGCCACCCTGTGTGGCTCGGTCCCGACACAACAACAAACCCCGGCGCGGAATGCGCCAAGGAATCACAAACACAAGGCGTGCCCCCTCGACCCGGAAGCGGTGTTCGTCTGGGTGGCGTCGCAAAAAATTGAGTCCAAAATGACTCTCGGCAACGGATATCTCGGCTCTTGCATCGATGAAGAACGTAGCGAAATGCGATACTTGGTGTGAATTGCAGAATCCCGTGAACCATCGAGTCTTTGAACGCAAGTTGCGCCCGAAGCCATTAGGCTAAGGGCACGCCTGCCTGGGTGTCACCAATCGTTGCCCCCAACCTCACTGCCTTGTTGCGTGGAGAAGGGGTGAATGATGGCTTCCCGTGAGCACAGTCTCGCGGTTGGCTGAAAACGTTCTCCGTGCCGGCGTGCAGTGCCGTGACACTTGGTGGTTGAGTTTACCCTCGAGGCCAGTCACGTGTGCTCCCTGTCGGTTCCGGAAGCATGGACCCGTGAGCGGCAAAGACCGCCCTTGATGCGACCTCAGGTCAGGCGGGGCTACCCGCTGAGTTTAAGCATATCAATAAGCG

>Aindica_4x_205

GTAACAAGGTTTCCGTAgGTGAACCTGCGGAAGGatCATTGTTGATGCCTCGACCCaGCTAGACCCGCGAATGTGTTTTACAACTCGGGGTGATCGGGTTGCCTAGGCAGCTCGCCTCCCCGACCCGTTGGATCTCTGGCCACCCTGTGTGGCTCGGTCCCGACACAACAACAAACCCCGGCGCGGAATGCGCCAAGGAATCACAATCACAAGGCGTGCCCCCTCGACCCGGAAGCGGTGTTCGTCTGGGTGGCGTCGCAAAAAATTGAGTCCAAAATGACTCTCGGCAACGGATATCTCGGCTCTTGCATCGATGAAGAACGTAGCGAAATGCGATACTTGGTGTGAATTGCAGAATCCCGTGAACCATCGAGTCTTTGAACGCAAGTTGCGCCCGAAGCCATTAGGCTAAGGGCACGCCTGCCTGGGTGTCACCAATCGTCGCCCCCAACCTCACTGCCTTGTTGCGTGGAGAAGGGGTGAATGATGGCTTCCCGTGAGCACAGTCTCGCGGTTGGCTGAAAATGTTCTCCGTGCCGGCGTGCAGTGCCGTGACACTTGGTGGTTGAGTTTACCCTCGAGGCCAGTCACGTGTGCTCCCTGTCGGTTCCGGAAGCATGGACCCGTGAGCGGCAAAGACCGCCCTTGATGCGACCTCAGGTCAGGCGGGGCTACCCGCTGAGTTTAAGCATATCAATAAGCG

>Aindica_4x_207

GTAACAAGGTTTCCGTAGGTGAaCCTGCGGAAGGatcATTGTTGAtGCCTCGACCCaGCTAGACCCGCGAATGTGTTTTACAACTCGGGGTGATCGGGTTGCCTAGGCAGCTCGCCTCCCCGACCCGTTGGATCTCTGGCCACCCTGTGTGGCTCGGTCCCGACACAACAACAAACCCCGGCGCGGAATGCGCCAAGGAATCACAATCACAAGGCGTGCCCCCTCGACCCGGAAGCGGTGTTCGTCTGGGTGGCGTCGCAAAAAATTGAGTCCAAAATGACTCTCGGCAACGGATATCTCGGCTCTTGCATCGATGAAGAACGTAGCGAAATGCGATACTTGGTGTGAATTGCAGAATCCCGTGAACCATCGAGTCTTTGAACGCAAGTTGCGCCCGAAGCCATTAGGCTAAGGGCACGCCTGCCTGGGTGTCACCAATCGTCGCCCCCAACCTCACTGCCTTGTTGCGTGGAGAAGGGGTGAATGATGGCTTCCCGTGAGCACAGTCTCGCGGTTGGCTGAAAATGTTCTCCGTGCCGGCGTGCAGTGCCGTGACACTTGGTGGTTGAGTTTACCCTCGAGGCCAGTCACGTGTGCTCCCTGTCGGTTCCGGAAGCATGGACCCGTGAGCGGCAAAGACCGCCCTTGATGCGACCTCAGGTCAGGCGGGGCTACCCGCTGAGTTTAAGCATATCAATAAGCG

>Aindica_4x_210

GTAACAAGGTTTCCGTAGGTGAAcCTGCGGAAGGAtCATTGTTGATGCCTCGACCCAGCTAGACCCGCGAATGTGTTTTACAACTCGGGGTGATCGGGTTGCCTAGGCAGCTTGCCTCCCCGACCCGTTGGATCTCTGGCCACCCTGTGTGGCTCGGTCCCGACACAACAACAAACCCCGGCGCGGAATGCGCCAAGGAATCACAATCACAAGGCGTGCCCCCTCGACCCGGAAGCGGTGTTCGTCTGGGTGGCGTCGCAAAAAATTGAGTCCAAAATGACTCTCGGCAACGGATATCTCGGCTCTTGCATCGATGAAGAACGTAGCGAAATGCGATACTTGGTGTGAATTGCAGAATCCCGTGAACCATCGAGTCTTTGAACGCAAGTTGCGCCCGAAGCCATTAGGCTAAGGGCACGCCTGCCTGGGTGTCACCAATCGTCGCCCCCAACCTCACTGCCTTGTTGCGTGGAGAAGGGGTGAATGATGGCTTCCCGTGAGCACAGTCTCGCGGTTGGCTGAAAATGTTCTCCGTGCCGGCGTGCAGTGCCGTGACACTTGGTGGTTGAGTTTACCCTCGAGGCCAGTCACGTGTGCTCCCTGTCGGTTCCGGAAGCATGGACCCGTGAGCGGCAAAGACCGCCCTTGATGCGACCTCAGGTCAGGCGGGGCTACCCGCTGAGTTTAAGCATATCAATAAGCG

>Aindica_4x_211

GTAACAAGGTTTCCGTAGGTGAACcTGCGGAAGGATCATTGTTGATGCCTCGACCCAGCTAGACCCGCGAATGTGTTTTACAACTCGGGGTGATCGGGTTGCCTAGGCAGCTCGCCTCCCCGACCCGTTGGATCTCTGGCCACCCTGTGTGGCTCGGTCCCGACACAACAACAAACCCCGGCGCGGAATGCGCCAAGGAATCACAATCACAAGGCGTGCCCCCTCGACCCGGAAGCGGTGTTCGTCTGGGTGGCGTCGCAAAAAATTGAGTCCAAAATGACTCTCGGCAACGGATATCTCGGCTCTTGCATCGATGAAGAACGTAGCGAAATGCGATACTTGGTGTGAATTGCAGAATCCCGTGAACCATCGAGTCTTTGAACGCAAGTTGCGCCCGAAGCCATTAGGCTAAGGGCACGCCTGCCTGGGTGTCACCAATCGTCGCCCCCAACCTCACTGCCTTGTTGCGTGGAGAAGGGGTGAATGATGGCTTCCCGTGAGCACAGTCTCGCGGTTGGCTGAAAATGTTCTCCGTGCCGGCGTGCAGTGCCGTGACACTTGGTGGTTGAGTTTACCCTCGAGGCCAGTCACGTGTGCTCCCTGTCGGTTCCGGAAGCATGGACCCGTGAGCGGCAAAGACCGCCCTTGATGCGACCTCAGGTCAGGCGGGGCTACCCGCTGAgTTTAAGCATATCAATAAGCG

>Aindica_4x_220

GTAACAAGGTTTCCGTAGGTGAACcTGCGGAAGGATCATTGTTGATGCCTCGACCCAGCTAGACCCGCGAATGTGTTTTACAACTCGGGGTGATCGGGTTGCCTAGGCAGCTCGCCTCCCCGACCCGTTGGATCTCTGGCCACCCTGTGTGGCTCGGTCCCGACACAACAACAAACCCCGGCGCGGAATGCGCCAAGGAATCACAATCACAAGGCGTGCCCCCTCGACCCGGAAGCGGTGTTCGTCTGGGTGGCGTCGCAAAAAATTGAGTCCAAAATGACTCTCGGCAACGGATATCTCGGCTCTTGCATCGATGAAGAACGTAGCGAAATGCGATACTTGGTGTGAATTGCAGAATCCCGTGAACCATCGAGTCTTTGAACGCAAGTTGCGCCCGAAGCCATTAGGCTAAGGGCACGCCTGCCTGGGTGTCACCAATCGTCGCCCCCAACCTCACTGCCTTGTTGCGTGGAGAAGGGGTGAATGATGGCTTCCCGTGAGCACAGTCTCGCGGTTGGCTGAAAATGTTCTCCGTGCCGGCGTGCAGTGCCGTGACACTTGGTGGTTGAGTTTACCCTCGAGGCCAGTCACGTGTGCTCCCTGTCGGTTCCGGAAGCATGGACCCGTGAGCGGCAAAGACCGCCCTTGATGCGACCTCAGGTCAGGCGGGGCTACCCGCTGAgTTTAAGCATATCAATAAGCG

>Aindica_4x_226

GTAACAAGGTTTCCGTAGGTGAACCTGCGGAAGGAtCATTGTTGATGCCTCGACCCAGCTAGACCCGCGAATGTGTTTTACAACTCGGGGTGATCGGGTTGCCTAGGCAGCTCGCCTCCCCGACCCGTTGGGTCTTTGGCCACCCTGTGTGGCTCGGTCCCGACACAACAACAAACCCCGGCGCGGAATGCGCCAAGGAATCACAAACACAAGGCGTGCCCCCTCGACCCGGAAGCGGTGTTCGTCTGGGTGGCGTCGCAAAAAATTGAGTCCAAAATGACTCTCGGCAACGGATATCTCGGCTCTTGCATCGATGAAGAACGTAGCGAAATGCGATACTTGGTGTGAATTGCAGAATCCCGTGAACCATCGAGTCTTTGAACGCAAGTTGCGCCCGAAGCCATTAGGCTAAGGGCACGCCTGCCTGGGTGTCACCAATCGTTGCCCCCAACCTCACTGCCTTGTTGCGTGGAGAAGGGGTGAATGATGGCTTCCCGTGAGCACAGTCTCGCGGTTGGCTGAAAACGTTCTCCGTGCCGGCGTGCAGTGCCGTGACACTTGGTGGTTGAGTTTACCCTCGAGGCCAGTCACGTGTGCTCCCTGTCGGTTCCGGAAGCATGGACCCGTGAGCGGCAAAGACCGCCCTTGATGCGACCTCAgGTCAgGCGGGGCTACCCGCTGAGTTTAAGCATATCAATAAGCG

>Aindica_4x_239

GTAACAAGGTTTCCGTAGGTGAACCTGCGGAAGGatCATTGTTGATGCCTCGACCCAGCTAGACCCGCGAATGTGTTTTACAACTCGGGGTGATCGGGTTGCCTAGGCAGCTCGCCTCCCCGACCCGTTGGGTCTCTGGCCACCCTGTGTGGCTCGGTCCCGACACAACAACAAACCCCGGCGCGGAATGCGCCAAGGAATCACAAACACAAGGCGTGCCCCCTCGACCCGGAAGCGGTGTTCGTCTGGGTGGCGTCGCAAAAAATTGAGTCCAAAATGACTCTCGGCAACGGATATCTCGGCTCTTGCATCGATGAAGAACGTAGCGAAATGCGATACTTGGTGTGAATTGCAGAATCCCGTGAACCATCGAGTCTTTGAACGCAAGTTGCGCCCGAAGCCATTAGGCTAAGGGCACGCCTGCCTGGGTGTCACCAATCGTTGCCCCCAACCTCACTGCCTTGTTGCGTGGAGAAGGGGTGAATGATGGCTTCCCGTGAGCACAGTCTCGCGGTTGGCTGAAAACGTTCTCCGTGCCGGCGTGCAGTGCCGTGACACTTGGTGGTTGAGTTTACCCTCGAGGCCAGTCACGTGTGCTCCCTGTCGGTTCCGGAAGCATGGACCCGTGAGCGGCAAAGACCGCCCTTGATGCGACCTCAGGTCAGGCGGGGCTACCCGCTGAGTTTAAGCATATCAATAAGCG

>Aindica_4x_276

GTAACAAGGTTTCCgTAGGTGAaCCTGCGGAAGGaTCATTGTTGATGCCTCGACCCAGCTAGACCCGCGAATGTGTTTTACAACTCGGGGTGATCGGGTTGCCTAGGCAGCTCGCCTCCCCGACCCGTTGGATCTCTGGCCACCCTGTGTGGCTCGGTCCCGACACAACAACAAACCCCGGCGCGGAATGCGCCAAGGAATCACAATCACAAGGCGTGCCCCCTCGACCCGGAAGCGGTGTTCGTCTGGGTGGCGTCGCAAAAAATTGAGTCCAAAATGACTCTCGGCAACGGATATCTCGGCTCTTGCATCGATGAAGAACGTAGCGAAATGCGATACTTGGTGTGAATTGCAGAATCCCGTGAACCATCGAGTCTTTGAACGCAAGTTGCGCCCGAAGCCATTAGGCTAAGGGCACGCCTGCCTGGGTGTCACCAATCGTCGCCCCCAACCTCACTGCCTTGTTGCGTGGAGAAGGGGTGAATGATGGCTTCCCGTGAGCACAGTCTCGCGGTTGGCTGAAAATGTTCTCCGTGCCGGCGTGCAGTGCCGTGACACTTGGTGGTTGAGTTTACCCTCGAGGCCAGTCACGTGTGCTCCCTGTCGGTTCCGGAAGCATGGACCCGTGAGCGGCAAAGACCGCCCTTGATGCGACCTCAGGTCAGGCGGGGCTACCCGCTGAGTTTAAGCATATCAATAAGCG

>Aindica_4x_277

GTAACAAGGTTTCCGTAGGTGAACCTGCGGAAGGAtCATTGTTGATGCCTCGACCCAGCTAGACCCGCGAATGTGTTTTACAACTCGGGGTGATCGGGTTGCCTAGGCAGCTCGCCTCCCCGACCCGTTGGGTCTCTGGCCAACCCTGTGTGGCTCGGTCCCGACACAACAACAAACCCCGGCGCGGAATGCGCCAAGGAATCACAAACACAAGGCGTGCCCCCTCGACCCGGAAGCGGTGTTCGTCTGGGTGGCGTCGCAAAAAATTGAGTCCAAAATGACTCTCGGCAACGGATATCTCGGCTCTTGCATCGATGAAGAACGTAGCGAAATGCGATACTTGGTGTGAATTGCAGAATCCCGTGAACCATCGAGTCTTTGAACGCAAGTTGCGCCCGAAGCCATTAGGCTAAGGGCACGCCTGCCTGGGTGTCACCAATCGTTGCCCCCAACCTCACTGCCTTGTTGCGTGGAGAAGGGGTGAATGATGGCTTCCCGTGAGCACAGTCTCGCGGTTGGCTGAAAACGTTCTCCGTGCCGGCGTGCAGTGCCGTGACACTTGGTGGTTGAGTTTACCCTCGAGGCCAGTCACGTGTGCTCCCTGTCGGTTCCGGAAGCATGGACCCGTGAGCGGCAAAGACCGCCCTTGATGCGACCTCAGGTCAGGCGGGGCTACCCGCTGAGTTTAAGCATATCAATAAGCG

>Aindica_4x_278

GTAACAAGGTTTCCGTAGGTGAaCCTGCGGAAGGAtCATTGTTGATGCCTCGACCCAGCTAGACCCGCGAATGTGTTTTACAACTCGGGGTGATCGGGTTGCCTAGGTAGCTCGCCTCCCCGACCCGTTGGGTCTCTGGCCACCCTGTGTGGCTCGGTCCCGACACAACAACAAACCCCGGCGCGGAATGCGCCAAGGAATCACAAACACAAGGCGTGCCCCCTCGACCCGGAAGCGGTGTTCGTCTGGGTGGCGTCGCAAAAAATTGAGTCCAAAATGACTCTCGGCAACGGATATCTCGGCTCTTGCATCGATGAAGAACGTAGCGAAATGCGATACTTGGTGTGAATTGCAGAATCCCGTGAACCATCGAGTCTTTGAACGCAAGTTGCGCCCGAAGCCATTAGGCTAAGGGCACGCCTGCCTGGGTGTCACCAATCGTTGCCCCCAACCTCACTGCCTTGTTGCGTGGAGAAGGGGTGAATGATGGCTTCCCGTGAGCACAGTCTCGCGGTTGGCTGAAAACGTTCTCCGTGCCGGCGTGCAGTGCCGTGACACTTGGTGGTTGAGTTTACCCTCGAGGCCAGTCACGTGTGCTCCCTGTCGGTTCCGGAAGCATGGACCCGTGAGCGGCAAAGACCGCCCTTGATGCGACCTCAGGTCAGGCGGGGCTACCCGCTGAGTTTAAGCATATCAATAAGCG

>Aindica_4x_279

GTAACAAGGTTTCCGTAGGTGAacctGCGGaAGGatCATTGTTGATGCCTCGACCCAGCTAGACCCGCGAATGTGTTTTACAACTCGGGGTGATCGGGTTGCCTAGGCAGCTCGCCTCCCCGACCCGTTGGATCTCTGGCCACCCTGTGTGGCTCGGTCCCGACACAACAACAAACCCCGGCGCGGAATGCGCCAAGGAATCACAATCACAAGGCGTGCCCcCTCGACCCGGAAGCGGTGTTCGTCTGGGTGGCGTCGCAAAAAATTGAGTCCAAAATGACTCTCGGCAACGGATATCTCGGCTCTTGCATCGATGAAGAACGTAGCGAAATGCGATACTTGGTGTGAATTGCAGAATCCCGTGAACCATCGAGTCTTTGAACGCAAGTTGCGCCCGAAGCCATTAGGCTAAGGGCACGCCTGCCTGGGTGTCACCAATCGTCGCCCCCAACCTCACTGCCTTGTTGCGTGGAGAAGGGGTGAATGATGGCTTCCCGTGAGCACAGTCTCGCGGTTGGCTGAAAATGTTCTCCGTGCCGGCGTGCAGTGCCGTGACACTTGGTGGTTGAGTTTACCCTCGAGGCCAGTCACGTGTGCTCCCTGTCGGTTCCGGAAGCATGGACCCGTGAGCGGCAAAGACCGCCCTTGATGCGACCTCAGGTCAGGCGGGGCTACCCGCTGAGTTTAAGCATATCAATAAGCG

>Aindica_4x_280

GTAACAAGGTTTCCGTAGGTGAaCCTGCGGAAGGaTCATTGTTGATGCCTCGACCCAGCTAGACCCGCGAATGTGTTTTACAACTCGGGGTGATCGGGTTGCCTAGGTAGCTCGCCTCCCCGACCCGTTGGGTCTCTGGCCACCCTGTGTGGCTCGGTCCCGACACAACAACAAACCCCGGCGCGGAATGCGCCAAGGAATCACAAACACAAGGCGTGCCCCCTCGACCCGGAAGCGGTGTTCGTCTGGGTGGCGTCGCAAAAAATTGAGTCCAAAATGACTCTCGGCAACGGATATCTCGGCTCTTGCATCGATGAAGAACGTAGCGAAATGCGATACTTGGTGTGAATTGCAGAATCCCGTGAACCATCGAGTCTTTGAACGCAAGTTGCGCCCGAAGCCATTAGGCTAAGGGCACGCCTGCCTGGGTGTCACCAATCGTTGCCCCCAACCTCACTGCCTTGTTGCGTGGAGAAGGGGTGAATGATGGCTTCCCGTGAGCACAGTCTCGCGGTTGGCTGAAAACGTTCTCCGTGCCGGCGTGCAGTGCCGTGACACTTGGTGGTTGAGTTTACCCTCGAGGCCAGTCACGTGTGCTCCCTGTCGGTTCCGGAAGCATGGACCCGTGAGCGGCAAAGACCGCCCTTGATGCGACCTCAGGTCAGGCGGGGCTACCCGCTGAGTTTAAGCATATCAATAAGCG

>Aindica_4x_357

GTAACAAGGTTTCCgTAGGTGAACCTGCGGAAGGATCATTGTTGATGCCTCGACCCAGCTAGACCCGCGAATGTGTTTTACAACTCGGGGTGATCGGGTTGCCTAGGCAGCTCGCCTCCCCGACCCGTTGGATCTCTGGCCACCCTGTGTGGCTCGGTCCCGACACAACAACAAACCCCGGCGCGGAATGCGCCAAGGAATCACAATCACAAGGCGTGCCCCCTCGACCCGGAAGCGGTGTTCGTCTGGGTGGCGTCGCAAAAAATTGAGTCCAAAATGACTCTCGGCAACGGATATCTCGGCTCTTGCATCGATGAAGAACGTAGCGAAATGCGATACTTGGTGTGAATTGCAGAATCCCGTGAACCATCGAGTCTTTGAACGCAAGTTGCGCCCGAAGCCATTAGGCTAAGGGCACGCCTGCCTGGGTGTCACCAATCGTCGCCCCCAACCTCACTGCCTTGTTGCGTGGAGAAGGGGTGAATGATGGCTTCCCGTGAGCACAGTCTCGCGGTTGGCTGAAAATGTTCTCCGTGCCGGCGTGCAGTGCCGTGACACTTGGTGGTTGAGTTTACCCTCGAGGCCAGTCACGTGTGCTCCCTGTCGGTTCCGGAAGCATGGACCCGTGAGCGGCAAAGACCGCCCTTGATGCGACCTCAGGTCAGGCGGGGCTACCCGCTGAGTTTAAGCATATCAATAAGCGG

>Aindica_6x_Africa_19-A

GTAACAAGGTTTCCGTAGGTGAACCTGCGGAAGGATCATTGTTGATGCCTCGACCCAGCTAGACCCGCGAATGTGTTTTACAACTCGGGGTGATCGGGTTGCCTAGGCAGCTCGCCTCCCCGACCCGTTGGGGCTCTGGCCACCCTGTGTGGCTCGGTCCCGACACAACAACAAACCCCGGCGCGGAATGCGCCAAGGAATCACAAACACAAGGCGTGCCCCCTCGACCCGGAAGCGGTGTTCGTCTGGGTGGCGTCGCAAAAAATTGAGTCCAAAATGACTCTCGGCAACGGATATCTCGGCTCTTGCATCGATGAAGAACGTAGCGAAATGCGATACTTGGTGTGAATTGCAGAATCCCGTGAACCATCGAGTCTTTGAACGCAAGTTGCGCCCGAAGCCATTAGGCTAAGGGCACGCCTGCCTGGGTGTCACCAATCGTCGCCCCCAACCTCACTGCCTTGTTGCGTGGAGAAGGGGTGAATGATGGCTTCCCGTGAGCACAGTCTCGCGGTTGGCTGAAAACGTTCTCCGTGCCGGCGTGCAGTGCCGTGACACTTGGTGGTTGAGTTTACCCTCGAGGCCAGTCACGTGTGCTCCCTGTCGGTTCCGGAAGCATGGACCCGTGAGCGGCAAAGACCGCCCTTGATGCGACCTCAGGTCAGGCGGGGCTACCCGCTGAGTTTAAGCATATCAATAAGCG

>Aindica_6x_Africa_19-B

GTAACAAGGTTTCCGTAGGTGAACCTGCGGAAGGATCATTGTTGATGCCTCGACCCAGCTAGACCCGCGAATGTGTTTTACTaCCCgGGGTGATCGGGCTGCCTAGGCAGCTCGCCTCCCCGACCCGTTGGGGCTCTGGCCACCCTGTGTGGCTCGGTCCCGACACAACAACAAACCCCGGCGCGGAATGCGCCAAGGAATAACAAACACAAGGCGTGCCCCCTCGACCCGGAAGCGGTGTTCGTCTGGGTGGCGTCGCAAAAAATTGAGTCCAAAATGACTCTCGGCAACGGATATCTCGGCTCTTGCATCGATGAAGAACGTAGCGAAATGCGATACTTGGTGTGAATTGCAGAATCCCGTGAACCATCGAGTCTTTGAACGCAAGTTGCGCCCGAAGCCATTAGGCTAAGGGCACGCCTGCCTGGGTGTCACCAATCGTCGCCCCCAACCTCACTGCCTTGTTGCGTGGAGAAGGGGTGAATGATGGCTTCCCGTGAGCACAGTCTCGTGGTTGGCTGAAAACGTTCTCCGTGCCGGCGTGCAGCGCCGTGACACTTGGTGGTTGAGTTTACCCTCGAGGCCAGTCACGTGTGCTCCCTGTCGGTTTCGGAAGCATGGACCCGTGAGCGGCAAAGATCGCCCTTGATGCGACCTCAGGTCAGGCGGGGCTACCCGCTGAGTTTAAGCATATCAATAAGCG

>Aindica_6x_Africa_29-A

GTAACAAGGTTTCCGTAGGTGAACCTGCGGAAGGATCATTGTTGATGCCTCGACCCAGCTAGACCCGCGAATGTGTTTTACAACTCgGGGTGATCGGGTTGCCTAGGCAGCTCGCCTCCCCGACCCGTTGGGGCTCTGGCCACCCTGTGTGGCTCGGTCCCGACACAACAACAAACCCCGGCGCGGAATGCGCCAAGGAATCACAAACACAAGGCGTGCCCCCTCGACCCGGAAGCGGTGTTCGTCTGGGTGGCGTCGCAAAAAATTGAGTCCAAAATGACTCTCGGCAACGGATATCTCGGCTCTTGCATCGATGAAGAACGTAGCGAAATGCGATACTTGGTGTGAATTGCAGAATCCCGTGAACCATCGAGTCTTTGAACGCAAGTTGCGCCCGAAGCCATTAGGCTAAGGGCACGCCTGCCTGGGTGTCACCAATCGTCGCCCCCAACCTCACTGCCTTGTTGCGTGGAGAAGGGGTGAATGATGGCTTCCCGTGAGCACAGTCTCGTGGTTGGCTGAAAACGTTCTCCGTGCCGGCGTGCAGTGCCGTGACACTTGGTGGTTGAGTTTACCCTCGAGGCCAGTCACGTGTGCTCCCTGTCGGTTCCGGAAGCATGGACCCGTGAGCGGCAAAGACCGCCCTTGATGCGACCTCAGGTCAGGCGGGGCTACCCGCTGAGTTTAAGCATATCAATAAGCG

>Aindica_6x_Africa_29-B

GTAACAAGGTTTCCGTAGGTGAACCTGCGGAAGGATCATTGTTGATGCCTCGACCCAGCTAGACCCGCGAATGTGTTTTACTaCCCgGGGTGATCGGGCTGCCTAGGCAGCTCGCCTCCCCGACCCGTTGGGGCTCTGGCCACCCTGTGTGGCTCGGTCCCGACACAACAACAAACCCCGGCGCGGAATGCGCCAAGGAATAACAAACACAAGGCGTGCCCCCTCGACCCGGAAGCGGTGTTCGTCTGGGTGGCGTCGCAAAAAATTGAGTCCAAAATGACTCTCGGCAACGGATATCTCGGCTCTTGCATCGATGAAGAACGTAGCGAAATGCGATACTTGGTGTGAATTGCAGAATCCCGTGAACCATCGAGTCTTTGAACGCAAGTTGCGCCCGAAGCCATTAGGCTAAGGGCACGCCTGCCTGGGTGTCACCAATCGTCGCCCCCAACCTCACTGCCTTGTTGCGTGGAGAAGGGGTGAATGATGGCTTCCCGTGAGCACAGTCTCGTGGTTGGCTGAAAACGTTCTCCGTGCCGGCGTGCAGCGCCGTGACACTTGGTGGTTGAGTTTACCCTCGAGGCCAGTCACGTGTGCTCCCTGTCGGTTTCGGAAGCATGGACCCGTGAGCGGCAAAGATCGCCCTTGATGCGACCTCAGGTCAGGCGGGGCTACCCGCTGAGTTTAAGCATATCAATAAGCG

>Aindica_6x_Africa_44-A

GTAACAAGGTTTCCGTAGGTGAACCTGCGGAAGGATCATTGTTGATGCCTCGACCCAGCTAGACCCGCGAATGTGTTTTACTACTCGGGGTGATCGGGTTGCCTAGGCAGCTCGCCTCCCCGACCCGTTGGGGCTCTGGCCACCCTGTGTGGCTCGGTCCCGACACAACAACAAACCCCGGCGCGGAATGCGCCAAGGAATAACAAACACAAGGCGTGCCCCCTCGACCCGGAAGCGGTGTTCGTCTGGGTGGCGTCGCAAAAAAATTGAGTCCAAAATGACTCTCGGCAACGGATATCTCGGCTCTTGCATCGATGAAGAACGTAGCGAAATGCGATACTTGGTGTGAATTGCAGAATCCCGTGAACCATCGAGTCTTTGAACGCAAGTTGCGCCCGAAGCCATTAGGCTAAGGGCACGCCTGCCTGGGTGTCACCAATCGTCGCCCCCAACCTCACTGCCTTGTTGCGTGGAGAAGGGGTGAATGATGGCTTCCCGTGAGCACAGTCTCGCGGTTGGCTGAAAACGTTCTCCGTGCCGGCGTGCAGCGCCGTGACACTTGGTGGTTGAGTTTACCCTCGAGGCCAGTCACGTGTGCTCCCTGTCGGTTCCGGAAGCATGGACCCGTGAGCGGCAAAGACCGCCCTTGATGCGACCTCAGGTCAGGCGGGGCTACCCGCTGAGTTTAAGCATATCAATAAGCG

>Aindica_6x_Africa_44-B

GTAACAAGGTTTCCGTAGGTGAACCTGCGGAAGGATCATTGTTGATGCCTCGACCCAGCTAGACCCGCGAATGTGTTTTACTaCCCgGGGTGATCGGGCTGCCTAGGCAGCTCGCCTCCCCGACCCGTTGGGGCTCTGGCCACCCTGTGTGGCTCGGTCCCGACACAACAACAAACCCCGGCGCGGAATGCGCCAAGGAATAACAAACACAAGGCGTGCCCCCTCGACCCGGAAGCGGTGTTCGTCTGGGTGGCGTCGCAAAAAATTGAGTCCAAAATGACTCTCGGCAACGGATATCTCGGCTCTTGCATCGATGAAGAACGTAGCGAAATGCGATACTTGGTGTGAATTGCAGAATCCCGTGAACCATCGAGTCTTTGAACGCAAGTTGCGCCCGAAGCCATTAGGCTAAGGGCACGCCTGCCTGGGTGTCACCAATCGTCGCCCCCAACCTCACTGCCTTGTTGCGTGGAGAAGGGGTGAATGATGGCTTCCCGTGAGCACAGTCTCGTGGTTGGCTGAAAACGTTCTCCGTGCCGGCGTGCAGCGCCGTGACACTTGGTGGTTGAGTTTACCCTCGAGGCCAGTCACGTGTGCTCCCTGTCGGTTTCGGAAGCATGGACCCGTGAGCGGCAAAGATCGCCCTTGATGCGACCTCAGGTCAGGCGGGGCTACCCGCTGAGTTTAAGCATATCAATAAGCG

>Aindica_6x_Africa_46-A

GTAACAAGGTTTCCGTAGGTGAACCTGCGGAAGGATCATTGTTGATGCCTCGACCCAGCTAGACCCGCGAATGTGTTTTACTACTCGGGGTGATCGGGTTGCCTAGGCAGCTCGCCTCCCCGACCCGTTGGGGCTCTGGCCACCCTGTGTGGCTCGGTCCCGACACAACAACAAACCCCGGCGCGGAATGCGCCAAGGAATCACAAACACAAGGCGTGCCCCCTCGACCCGGAAGCGGTGTTCGTCTGGGTGGCGTCGCAAAAAATTGAGTCCAAAATGACTCTCGGCAACGGATATCTCGGCTCTTGCATCGATGAAGAACGTAGCGAAATGCGATACTTGGTGTGAATTGCAGAATCCCGTGAACCATCGAGTCTTTGAACGCAAGTTGCGCCCGAAGCCATTAGGCTAAGGGCACGCCTGCCTGGGTGTCACCAATCGTCGCCCCCAACCTCACTGCCTTGTTGCGTGGAGAAGGGGTGAATGATGGCTTCCCGTGAGCACAGTCTCGCGGTTGGCTGAAAACGTTCTCCGTGCCGGCGTGCAGCGCCGTGACACTTGGTGGTTGAGTTTACCCTCGAGGCCAGTCACGTGTGCTCCCTGTCGGTTTCGGAAGCATGGACCCGTGAGCGGCAAAGATCGCCCTTGATGCGACCTCAGGTCAGGCGGGGCTACCCGCTGAGTTTAAGCATATCAATAAGCG

>Aindica_6x_Africa_46-B

GTAACAAGGTTTCCGTAGGTGAACCTGCGGAAGGATCATTGTTGATGCCTCGACCCAGCTAGACCCGCGAATGTGTTTTACTaCCCgGGGTGATCGGGCTGCCTAGGCAGCTCGCCTCCCCGACCCGTTGGGGCTCTGGCCACCCTGTGTGGCTCGGTCCCGACACAACAACAAACCCCGGCGCGGAATGCGCCAAGGAATAACAAACACAAGGCGTGCCCCCTCGACCCGGAAGCGGTGTTCGTCTGGGTGGCGTCGCAAAAAATTGAGTCCAAAATGACTCTCGGCAACGGATATCTCGGCTCTTGCATCGATGAAGAACGTAGCGAAATGCGATACTTGGTGTGAATTGCAGAATCCCGTGAACCATCGAGTCTTTGAACGCAAGTTGCGCCCGAAGCCATTAGGCTAAGGGCACGCCTGCCTGGGTGTCACCAATCGTCGCCCCCAACCTCACTGCCTTGTTGCGTGGAGAAGGGGTGAATGATGGCTTCCCGTGAGCACAGTCTCGTGGTTGGCTGAAAACGTTCTCCGTGCCGGCGTGCAGCGCCGTGACACTTGGTGGTTGAGTTTACCCTCGAGGCCAGTCACGTGTGCTCCCTGTCGGTTTCGGAAGCATGGACCCGTGAGCGGCAAAGATCGCCCTTGATGCGACCTCAGGTCAGGCGGGGCTACCCGCTGAGTTTAAGCATATCAATAAGCG

>Aindica_6x_Africa_93-A

GTAACAAGGTTTCCGTAGGTGAACCTGCGGAAGGATCATTGTTGATGCCTCGACCCAGCTAGACCCGCGAATGTGTTTTACTACCCGGGGTGATCGGGCTGCCTAGGCAGCTCGCCTCCCCGACCCGTTGGGGCTCTGGCCACCCTGTGTGGCTCGGTCCCGACACAACAACAAACCCCGGCGCGGAATGCGCCAAGGAATAACAAACACAAGGCGTGCCCCCTCGACCCGGAAGCGGTGTTCGTCTGGGTGGCGTCGCAAAAAATTGAGTCCAAAATGACTCTCGGCAACGGATATCTCGGCTCTTGCATCGATGAAGAACGTAGCGAAATGCGATACTTGGTGTGAATTGCAGAATCCCGTGAACCATCGAGTCTTTGAACGCAAGTTGCGCCCGAAGCCATTAGGCTAAGGGCACGCCTGCCTGGGTGTCACCAATCGTCGCCCCCAACCTCACTGCCTTGTTGCGTGGAGAAGGGGTGAATGATGGCTTCCCGTGAGCACAGTCTCGTGGTTGGCTGAAAACGTTCTCCGTGCCGGCGTGCAGCGCCGTGACACTTGGTGGTTGAGTTTACCCTCGAGGCCAGTCACGTGTGCTCCCTGTCGGTTCCGGAAGCATGGACCCGTGAGCGGCAAAGACCGCCCTTGATGCGACCTCAGGTCAGGCGGGGCTACCCGCtGAGTTtAAGCATATCAATAAGCG

>Aindica_6x_Africa_93-B

GTAACAAGGTTTCCGTAGGTGAACCTGCGGAAGGATCATTGTTGATGCCTCGACCCAGCTAGACCCGCGAATGTGTTTTACTACCCGGGGTGATCGGGCTGCCTAGGCAGCTCGCCTCCCCGACCCGTTGGGGCTCTGGCCACCCTGTGTGGCTCGGTCCCGACACAACAACAAACCCCGGCGCGGAATGCGCCAAGGAATAACAAACACAAGGCGTGCCCCCTCGACCCGGAAGCGGTGTTCGTCTGGGTGGCGTCGCAAAAAAATTGAGTCCAAAATGACTCTCGGCAACGGATATCTCGGCTCTTGCATCGATGAAGAACGTAGCGAAATGCGATACTTGGTGTGAATTGCAGAATCCCGTGAACCATCGAGTCTTTGAACGCAAGTTGCGCCCGAAGCCATTAGGCTAAGGGCACGCCTGCCTGGGTGTCACCAATCGTCGCCACCAACCTCACTGCCTTGTTGCGTGGAGAAGGGGTGAATGATGGCTTCCCGTGAGCACAGTCTCGTGGTTGGCTGAAAACGTTCTCCGTGCCGGCGTGCAGCGCCGTGACACTTGGTGGTTGAGTTTACCCTCGAGGCCAGTCACGTGTGCTCCCTGTCGGTTCCGGAAGCATGGACCCGTGAGCGGCAAAGACCGCCCTTGATGCGACCTCAGGTCAGGCGGGGCTACCCGCTGAGTTTAAGCATATCAATAAGCG

>Aindica_6x_Africa_97-A

GTAACAAGGTTTCCGTAGGTGAACCTGCGGAAGGATCATTGTTGATGCCTCGACCCAGCTAGACCCGCGAATGTGTTTTACAACTCGGGGTGATCGGGTTGCCTAGGCAGCTCGCCTCCCCGACCCGTTGGGGCTCTGGCCACCCTGTGTGGCTCGGTCCCGACACAACAACAAACCCCGGCGCGGAATGCGCCAAGGAATCACAAACACAAGGCGTGCCCCCTCGACCCGGAAGCGGTGTTCGTCTGGGTGGCGTCGCAAAAAATTGAGTCCAAAATGACTCTCGGCAACGGATATCTCGGCTCTTGCATCGATGAAGAACGTAGCGAAATGCGATACTTGGTGTGAATTGCAGAATCCCGTGAACCATCGAGTCTTTGAACGCAAGTTGCGCCCGAAGCCATTAGGCTAAGGGCACGCCTGCCTGGGTGTCACCAATCGTCGCCCCCAACCTCACTGCCTTGTTGCGTGGAGAAGGGGTGAATGATGGCTTCCCGTGAGCACAGTCTCGCGGTTGGCTGAAAACGTTCTCCGTGCCGGCGTGCAGTGCCGTGACACTTGGTGGTTGAGTTTACCCTCGAGGCCAGTCACGTGTGCTCCCTGTCGGTTTCGGAAGCATGGACCCGTGAGCGGCAAAGACCGCCCTTGATGCGACCTCAGGTCAGGCGGGGCTACCCGCTGAGTTTAAGCATATCAATAAGCG

>Aindica_6x_Africa_97-B

GTAACAAGGTTTCCGTAGGTGAACCTGCGGAAGGATCATTGTTGATGCCTCGACCCAGCTAGACCCGCGAATGTGTTTTACTACCCGGGGTGATCGGGCTGCCTAGGCAGCTCGCCTCCCCGACCCGTTGGGGCTCTGGCCACCCTGTGTGGCTCGGTCCCGACACAACAACAAACCCCGGCGCGGAATGCGCCAAGGAATAACAAACACAAGGCGTGCCCCCTCGACCCGGAAGCGGTGTTCGTCTGGGTGGCGTCGCAAAAAAATTGAGTCCAAAATGACTCTCGGCAACGGATATCTCGGCTCTTGCATCGATGAAGAACGTAGCGAAATGCGATACTTGGTGTGAATTGCAGAATCCCGTGAACCATCGAGTCTTTGAACGCAAGTTGCGCCCGAAGCCATTAGGCTAAGGGCACGCCTGCCTGGGTGTCACCAATCGTCGCCACCAACCTCACTGCCTTGTTGCGTGGAGAAGGGGTGAATGATGGCTTCCCGTGAGCACAGTCTCGTGGTTGGCTGAAAACGTTCTCCGTGCCGGCGTGCAGCGCCGTGACACTTGGTGGTTGAGTTTACCCTCGAGGCCAGTCACGTGTGCTCCCTGTCGGTTCCGGAAGCATGGACCCGTGAGCGGCAAAGACCGCCCTTGATGCGACCTCAGGTCAGGCGGGGCTACCCGCTGAGTTTAAGCATATCAATAAGCG

>Aindica_6x_Africa_106-A

GTAACAAGGTTTCCGTAGGTGAACCTGCGGAAGGATCATTGTTGATGCCTCGACCCAGCTAGACCCGCGAATGTGTTTTACaACTCGGGGTGATCGGGTTGCCTAGGCAGCTCGCCTCCCCGACCCGTTGGGGCTCTGGCCACCCTGTGTGGCTCGGTCCCGACACAACAACAAACCCCGGCGCGGAATGCGCCAAGGAATCACAAACACAAGGCGTGCCCCCTCGACCCGGAAGCGGTGTTCGTCTGGGTGGCGTCGCAAAAAATTGAGTCCAAAATGACTCTCGGCAACGGATATCTCGGCTCTTGCATCGATGAAGAACGTAGCGAAATGCGATACTTGGTGTGAATTGCAGAATCCCGTGAACCATCGAGTCTTTGAACGCAAGTTGCGCCCGAAGCCATTAGGCTAAGGGCACGCCTGCCTGGGTGTCACCAATCGTCGCCCCCAACCTCACTGCCTTGTTGCGTGGAGAAGGGGTGAATGATGGCTTCCCGTGAGCACAGTCTCGcGGTTGGCTGAAAACGTTCTCCGTGCCGGCGTGCAGTGCCGTGACACTTGGTGGTTGAGTTTACCCTCGAGGCCAGTCACGTGTGCTCCCTGTCGGTTTCGGAAGCATGGACCCGTGAGCGGCAAAGATCGCCCTTGATGCGACCTCAGGTCAGGCGGGGCTACCCGCTGAGTTTAAGCATATCAATAAGCG

>Aindica_6x_Africa_106-B

GTAACAAGGTTTCCGTAGGTGAACCTGCGGAAGGATCATTGTTGATGCCTCGACCCAGCTAGACCCGCGAATGTGTTTTACTACCCGGGGTGATCGGGCTGCCTAGGCAGCTCGCCTCCCCGACCCGTTGGGGCTCTGGCCACCCTGTGTGGCTCGGTCCCGACACAACAACAAACCCCGGCGCGGAATGCGCCAAGGAATAACAAACACAAGGCGTGCCCCCTCGACCCGGAAGCGGTGTTCGTCTGGGTGGCGTCGCAAAAAAATTGAGTCCAAAATGACTCTCGGCAACGGATATCTCGGCTCTTGCATCGATGAAGAACGTAGCGAAATGCGATACTTGGTGTGAATTGCAGAATCCCGTGAACCATCGAGTCTTTGAACGCAAGTTGCGCCCGAAGCCATTAGGCTAAGGGCACGCCTGCCTGGGTGTCACCAATCGTCGCCACCAACCTCACTGCCTTGTTGCGTGGAGAAGGGGTGAATGATGGCTTCCCGTGAGCACAGTCTCGTGGTTGGCTGAAAACGTTCTCCGTGCCGGCGTGCAGCGCCGTGACACTTGGTGGTTGAGTTTACCCTCGAGGCCAGTCACGTGTGCTCCCTGTCGGTTCCGGAAGCATGGACCCGTGAGCGGCAAAGACCGCCCTTGATGCGACCTCAGGTCAGGCGGGGCTACCCGCTGAGTTTAAGCATATCAATAAGCG

>Aindica_6x_Africa_192-A

GTAACAAGGTTTCCGTAGGTGAACCTGCGGAAGGATCATTGTTGATGCCTCGACCCAGCTAGACCCGCGAATGTGTTTTACAACTCGGGGTGATCGGGTTGCCTAGGCAGCTCGCCTCCCCGACCCGTTGGGGCTCTGGCCACCCTGTGTGGCTCGGTCCCGACACAACAACAAACCCCGGCGCGGAATGCGCCAAGGAATCACAAACACAAGGCGTGCCCCCTCGACCCGGAAGCGGTGTTCGTCTGGGTGGCGTCGCAAAAAATTGAGTCCAAAATGACTCTCGGCAACGGATATCTCGGCTCTTGCATCGATGAAGAACGTAGCGAAATGCGATACTTGGTGTGAATTGCAGAATCCCGTGAACCATCGAGTCTTTGAACGCAAGTTGCGCCCGAAGCCATTAGGCTAAGGGCACGCCTGCCTGGGTGTCACCAATCGTCGCCCCCAACCTCACTGCCTTGTTGCGTGGAGAAGGGGTGAATGATGGCTTCCCGTGAGCACAGTCTCGCGGTTGGCTGAAAACGTTCTCCGTGCCGGCGTGCAGTGCCGTGACACTTGGTGGTTGAGTTTACCCTCGAGGCCAGTCACGTGTGCTCCCTGTCGGTTTCGGAAGCATGGACCCGTGAGCGGCAAAGACCGCCCTTGATGCGACCTCAGGTCAGGCGGGGCTACCCGCTGAGTTTAAGCATATCAATAAGCG

>Aindica_6x_Africa_192-B

GTAACAAGGTTTCCGTAGGTGAACCTGCGGAAGGATCATTGTTGATGCCTCGACCCAGCTAGACCCGCGAATGTGTTTTACTACCCGGGGTGATCGGGCTGCCTAGGCAGCTCGCCTCCCCGACCCGTTGGGGCTCTGGCCACCCTGTGTGGCTCGGTCCCGACACAACAACAAACCCCGGCGCGGAATGCGCCAAGGAATAACAAACACAAGGCGTGCCCCCTCGACCCGGAAGCGGTGTTCGTCTGGGTGGCGTCGCAAAAAAATTGAGTCCAAAATGACTCTCGGCAACGGATATCTCGGCTCTTGCATCGATGAAGAACGTAGCGAAATGCGATACTTGGTGTGAATTGCAGAATCCCGTGAACCATCGAGTCTTTGAACGCAAGTTGCGCCCGAAGCCATTAGGCTAAGGGCACGCCTGCCTGGGTGTCACCAATCGTCGCCACCAACCTCACTGCCTTGTTGCGTGGAGAAGGGGTGAATGATGGCTTCCCGTGAGCACAGTCTCGTGGTTGGCTGAAAACGTTCTCCGTGCCGGCGTGCAGCGCCGTGACACTTGGTGGTTGAGTTTACCCTCGAGGCCAGTCACGTGTGCTCCCTGTCGGTTCCGGAAGCATGGACCCGTGAGCGGCAAAGACCGCCCTTGATGCGACCTCAGGTCAGGCGGGGCTACCCGCTGAGTTTAAGCATATCAATAAGCG

>Aindica_6x_Africa_200-A

GTAACAAGGTTTCCGTAGGTGAaCCTGCGGAAGGAtCATTGTTGATGCCTCGACCCAGCTAGACCCGCGAATGTGTTTTACTACCCGGGGTGATCGGGCTGCCTAGGCAGCTCGCCTCCCCGACCCGTTGGGGCTCTGGCCACCCTGTGTGGCTCGGTCCCGACACAACAACAAACCCCGGCGCGGAATGCGCCAAGGAATAACAAACACAAGGCGTGCCCCCTCGACCCGGAAGCGGTGTTCGTCTGGGTGGCGTCGCAAAAAAATTGAGTCCAAAATGACTCTCGGCAACGGATATCTCGGCTCTTGCATCGATGAAGAACGTAGCGAAATGCGATACTTGGTGTGAATTGCAGAATCCCGTGAACCATCGAGTCTTTGAACGCAAGTTGCGCCCGAAGCCATTAGGCTAAGGGCACGCCTGCCTGGGTGTCACCAATCGTCGCCCCCAACCTCACTGCCTTGTTGCGTGGAGAAGGGGTGAATGATGGCTTCCCGTGAGCACAGTCTCGTGGTTGGCTGAAAACGTTCTCCGTGCCGGCGTGCAGCGCCGTGACACTTGGTGGTTGAGTTTACCCTCGAGGCCAGTCACGTGTGCTCCCTGTCGGTTCCGGAAGCATGGACCCGTGAGCGGCAAAGACCGCCCTTGATGCGACCTCAGGTCAGGCGGGGCTACCCGCTGAGTttAAGCaTAtCAATAAGCG

>Aindica_6x_Africa_200-B

GTAACAAGGTTTCCGTAGGTGAACCTGCGGAAGGATCATTGTTGATGCCTCGACCCAGCTAGACCCGCGAATGTGTTTTACTACCCGGGGTGATCGGGCTGCCTAGGCAGCTCGCCTCCCCGACCCGTTGGGGCTCTGGCCACCCTGTGTGGCTCGGTCCCGACACAACAACAAACCCCGGCGCGGAATGCGCCAAGGAATAACAAACACAAGGCGTGCCCCCTCGACCCGGAAGCGGTGTTCGTCTGGGTGGCGTCGCAAAAAAATTGAGTCCAAAATGACTCTCGGCAACGGATATCTCGGCTCTTGCATCGATGAAGAACGTAGCGAAATGCGATACTTGGTGTGAATTGCAGAATCCCGTGAACCATCGAGTCTTTGAACGCAAGTTGCGCCCGAAGCCATTAGGCTAAGGGCACGCCTGCCTGGGTGTCACCAATCGTCGCCACCAACCTCACTGCCTTGTTGCGTGGAGAAGGGGTGAATGATGGCTTCCCGTGAGCACAGTCTCGTGGTTGGCTGAAAACGTTCTCCGTGCCGGCGTGCAGCGCCGTGACACTTGGTGGTTGAGTTTACCCTCGAGGCCAGTCACGTGTGCTCCCTGTCGGTTCCGGAAGCATGGACCCGTGAGCGGCAAAGACCGCCCTTGATGCGACCTCAGGTCAGGCGGGGCTACCCGCTGAGTTTAAGCATATCAATAAGCG

>Aindica_6x_Autralia_22

GTAACAAGGTTTCCGTAGGTGAACCTGCGGAAGGATCATTGTTGATGCCTCGACCCAGCTAGACCCGCGAATGTGTTTTACAACTCGGGGTGATCGGGTTGCCTAGGCAGCTCGCCTCCCCGACCCGTTGGGGCTCTGGCCACCCTGTGTGGCTCGGTCCCGACACAACAACAAACCCCGGCGCGGAATGCGCCAAGGAATCACAAACACAAGGCGTGCCCCCTCGACCCGGAAGCGGTGTTCGTCTGGGTGGCGTCGCAAAAAATTGAGTCCAAAATGACTCTCGGCAACGGATATCTCGGCTCTTGCATCGATGAAGAACGTAGCGAAATGCGATACTTGGTGTGAATTGCAGAATCCCGTGAACCATCGAGTCTTTGAACGCAAGTTGCGCCCGAAGCCATTAGGCTAAGGGCACGCCTGCCTGGGTGTCACCAATCGTCGCCCCCAACCTCACTGCCTTGTTGCGTGGAGAAGGGGTGAATGATGGCTTCCCGTGAGCACAGTCTCGCGGTTGGCTGAAAACGTTCTCCGTGCCGGCGTGCAGTGCCGTGACACTTGGTGGTTGAGTTTACCCTCGAGGCCAGTCACGTGTGCTCCCTGTCGGTTCCGGAAGCATGGACCCGTGAGCGGCAAAGACCGCCCTTGATGCGACCTCAGGTCAGGCGGGGCTACCCGCTGAGTTTAAGCATATCAATAAGCG

>Aindica_6x_Autralia_100

GTAACAAGGTTTCCGTAGGTGAACCTGCGGAAGGATCATTGTTGATGCCTCGACCCAGCTAGACCCGCGAATGTGTTTTACAACTCGGGGTGATCGGGTTGCCTAGGCAGCTCGCCTCCCCGACCCGTTGGGGCTCTGGCCACCCTGTGTGGCTCGGTCMCGACACAACAACAAACCCCGGCGCGGAATGCGCCAAGGAATCACAAACACAAGGCGTGCCCCCTCGACCCGGWMRCGGTGTTCGTSTGGGTGGCGTCGCAAAAAATTGAGTCCAAAATGACTCTCGGCAACGGATATCTCGGCTCTTGCATCGATGAAGAACGTAGCGAAATGCGATACTTGGTGTGAATTGCAGAATCCCGTGAACCATCGAGTCTTTGAACGCAAGTTGCGCCCGAAGCCATTAGGCTAAGGGCACGCCTGCCTGGGTGTCACCAATCGTCGCCCCCAACCTCACTGCCTTGTTGCGTGGAGAAGGGGTGAATGATGGCTTCCCGTGAGCACAGTCTCGCGGTTGGCTGAAAACGTTCTCCGTGCCGGCGTGCAGTGCCGTGACACTTGGTGGTTGAGTTTACCCTCGAGGCCAGTCACGTGTGCTCCCTGTCGGTTCCGGAAGCATGGACCCGTGAGCGGCAAAGACCGCCCTTGATGCGACCTCAGGTCAGGCGGGGCTACCCGCTGAGTTTAAGCATATCAATAAGCG

>Aindica_6x_Autralia_154

GTAACAAGGTTTCCGTAGGTGAACCTGCGGAAGGATCATTGTTGATGCCTCGACCCAGCTAGACCCGCGAATGTGTTTTACAACTCGGGGTGATCGGGTTGCCTAGGCAGCTCGCCTCCCCGACCCGTTGGGGCTCTGGCCACCCTGTGTGGCTCGGTCCCGACACAACAACAAACCCCGGCGCGGAATGCGCCAAGGAATCACAAACACAAGGCGTGCCCCCTCGACCCGGAAGCGGTGTTCGTCTGGGTGGCGTCGCAAAAAATTGAGTCCAAAATGACTCTCGGCAACGGATATCTCGGCTCTTGCATCGATGAAGAACGTAGCGAAATGCGATACTTGGTGTGAATTGCAGAATCCCGTGAACCATCGAGTCTTTGAACGCAAGTTGCGCCCGAAGCCATTAGGCTAAGGGCACGCCTGCCTGGGTGTCACCAATCGTCGCCCCCAACCTCACTGCCTTGTTGCGTGGAGAAGGGGTGAATGATGGCTTCCCGTGAGCACAGTCTCGCGGTTGGCTGAAAACGTTCTCCGTGCCGGCGTGCAGTGCCGTGACACTTGGTGGTTGAGTTTACCCTCGAGGCCAGTCACGTGTGCTCCCTGTCGGTTCCGGAAGCATGGACCCGTGAGCGGCAAAGACCGCCCTTGATGCGACCTCAGGTCAGGCGGGGCTACCCGCTGAGTTTAAGCATATCAATAAGCG

>Aindica_6x_Autralia_206

GTAACAAGGTTTCCGTAGGTGAACCTGCGGAAGGATCATTGTTGATGCCTCGACCCAGCTAGACCCGCGAATGTGTTTTACAACTCGGGGTGATCGGGTTGCCTAGGCAGCTCGCCTCCCCGACCCGTTGGGGCTCTGGCCACCCTGTGTGGCTCGGTCMCGACACAACAACAAACCCCGGCGCGGAATGCGCCAAGGAATCACAAACACAAGGCGTGCCCCCTCGACCCGGWMRCGGTGTTCGTSTGGGTGGCGTCGCAAAAAATTGAGTCCAAAATGACTCTCGGCAACGGATATCTCGGCTCTTGCATCGATGAAGAACGTAGCGAAATGCGATACTTGGTGTGAATTGCAGAATCCCGTGAACCATCGAGTCTTTGAACGCAAGTTGCGCCCGAAGCCATTAGGCTAAGGGCACGCCTGCCTGGGTGTCACCAATCGTCGCCCCCAACCTCACTGCCTTGTTGCGTGGAGAAGGGGTGAATGATGGCTTCCCGTGAGCACAGTCTCGCGGTTGGCTGAAAACGTTCTCCGTGCCGGCGTGCAGTGCCGTGACACTTGGTGGTTGAGTTTACCCTCGAGGCCAGTCACGTGTGCTCCCTGTCGGTTCCGGAAGCATGGACCCGTGAGCGGCAAAGACCGCCCTTGATGCGACCTCAGGTCAGGCGGGGCTACCCGCTGAGTTTAAGCATATCAATAAGCG

>Aindica_6x_Autralia_240

GTAACAAgGTTTCCgTAGGTGAACCTGCGGAAGGatCATTGTTGATGCCTCGACCCAGCTAGACCCGCGAATGTGTTTTACAACTCGGGGTGATCGGGTTGCCTAGGCAGCTCGCCYCCCCGACCCGTTGGGGCTCTGGCCACCCTGTGTGGCTCGGTCCCGACACAACAACAAACCCCGGCGCGGAATGCGCCAAGGAATCACAAACACAAGGCGTGCCCCCTCGACCCGGAAGCGGTGTTCGTCTGGGTGGCGTCGCAAAAAATTGAGTCCAAAATGACTCTCGGCAACGGATATCTCGGCTCTTGCATCGATGAAGAACGTAGCGAAATGCGATACTTGGTGTGAATTGCAGAATCCCGTGAACCATCGAGTCTTTGAACGCAAGTTGCGCCCGAAGCCATTAGGCTAAGGGCACGCCTGCCTGGGTGTCACCAATCGTCGCCCCCAACCTCACTGCCTTGTTGCGTGGAGAAGGGGTGAATGATGGCTTCCCGTGAGCACAGTCTCGCGGTTGGCTGAAAACGTTCTCCGTGCCGGCGTGCAGTGCCGTGACACTTGGTGGTTGAGTTTACCCTCGAGGCCAGTCACGTGTGCTCCCTGTCGGTTCCGGAAGCATGGACCCGTGAGCGGCAAAGACCGCCCTTGATGCGACCTCAGGTCAGGCGGGGCTACCCGCTGAGTTTAAGCATATCAATAAGCG

>Aindica_6x_Autralia_241

GTAACAAgGTTTCCGTAGGTGAacCTGCGGAAGGatCATTGTTGATGCCTCgACCCAGCTAGACCCGCGAATGTGTTTTACAACTCGGGGTGATCGGGTTGCCTAGGCAGCTCGCCTCCCCGACCCGTTGGGGCTCTGGCCACCCTGTGTGGCTCGGTCcCGACACAACAACAAACCCCGGCGCGGAATGCGCCAAGGAATCACAAACACAAGGCGTGCCCCCTCGACCCGGAAGCGGTGTTCGTCTGGGTGGCGTCGCAAAAAATTGAGTCCAAAATGACTCTCGGCAACGGATATCTCGGCTCTTGCATCGATGAAGAACGTAGCGAAATGCGATACTTGGTGTGAATTGCAGAATCCCGTGAACCATCGAGTCTTTGAACGCAAGTTGCGCCCGAAGCCATTAGGCTAAGGGCACGCCTGCCTGGGTGTCACCAATCGTCGCCCCCAACCTCACTGCCTTGTTGCGTGGAGAAGGGGTGAATGATGGCTTCCCGTGAGCACAGTCTCGCGGTTGGCTGAAAACGTTCTCCGTGCCGGCGTGCAGTGCCGTGACACTTGGTGGTTGAGTTTACCCTCGAGGCCAGTCACGTGTGCTCCCTGTCGGTTcCGGAAGCATGGACCCGTGAGCGGCAAAGACCGCCCTTGATGCGACCTCAgGTCAGGCGGGGCTACCCGCTGAGTTTAAGCATATCAATAAGCG

>Aindica_6x_Autralia_242

GTAACAAgGTTTCCGTAGGTGAacCTGCGGAAGGatcATTGTTGATGCCTCGACCCAGCTAGACCCGYGAATGTGTTTTACAACTCGGGGTGATCGGGTTGCCTAGGCAGCTCGCCTCCCCGACCCGTTGGGGCTCTGGCCACCCTGTGTGGCTMGGTCMCGACACAACAACAAACCCCGGCGCGGAATGCGCCAAGGAATCACAAACACAAGGCGTGCCCCCTCGACCCGGAAGCGGTGTTCGTCTGGGTGGCGTCGCAAAAAAWTGAGTCCAAAATGACTCTCGGCAACGGATATCTCGGCTCTTGCATCGATGAAGAACGTAGCGAAATGCGATACTTGGTGTGAATTGCAGAATCCCGTGAACCATCGAGTCTTTGAACGCAAGTTGCGCCCGAAGCCATTAGGCTAAGGGCACGCCTGCCTGGGTGTCACCAATCGTCGCCCCCAACCTCACTGCCTTGTTGCGTGGAGAAGGGGTGAATGATGGCTTCCCGTGAGCACAGTCRCGCGGTTGGCTGAAAACGTTCTCCGTGCCGGCGTGCRGTGCCGTGACACTTGGTGGTTGAGTTTACCCTCGAGGCCAGTCACGTGTGCTCCCTGTCGGTTCCGGAAGCATGGACCCGTGAGCGGCAAAGACCGCCCTTGATGCGACCTCAGGTCAGGCGGGGCTACCCGCTGAGTTTAAGCATATCAATAAGCG

>Aindica_6x_Autralia_243

GTAACAAgGTTTCCGTAGGTGAaCCTGCGGAAGGatCATTGTTGATGCCTCGACCCAGCTAGACCCGCGAATGTGTTTTACAACTCGGGGTGATCGGGTTGCCTAGGCAGCTCGCCYCCCCGACCCGTTGGGGCTCTGGCCACCCTGTGTGGCTCGGTCMCGACACAACAACAAACCCCGGCGCGGAATGCGCCAAGGAATCACAAACACAAGGCGTGCCCCCTCGACCCGGAAGCGGTGTTCGTCTGGGTGGCGTCGCAAAAAATTGAGTCCAAAATGACTCTCGGCAACGGATATCTCGGCTCTTGCATCGATGAAGAACGTAGCGAAATGCGATACTTGGTGTGAATTGCAGAATCCCGTGAACCATCGAGTCTTTGAACGCAAGTTGCGCCCGAAGCCATTAGGCTAAGGGCACGCCTGCCTGGGTGTCACCAATCGTCGCCCCCAACCTCACTGCCTTGTTGCGTGGAGAAGGGGTGAATGATGGCTTCCCGTGAGCACAGTCTCGCGGTTGGCTGAAAACGTTCTCCGTGCCGGCGTGCAGTGCCGTGACACTTGGTGGTTGAGTTTACCCTCGAGGCCAGTCACGTGTGCTCCCTGTCGGTTCCGGAAGCATGGACCCGTGAGCGGCAAAGACCGCCCTTGATGCGACCTCAGGTCAGGCGGGGCTACCCGcTGAGTTTAAGCATATCAaTAAGCG

>Aindica_6x_Autralia_244

GTAACAAgGTTTCCGTAgGTGAACCTGCGGAAGGatcATTGTTGATGCCTCGACCCAGCTAGACCCGCGAATGTGTTTTACAACTCGGGGTGATCGGGTTGCCTAGGCAGCTCGCCYCCCCGACCCGTTGGGGCTCTGGCCACCCTGTGTGGCTCGGTCMCGACACAACAACAAACCCCGGCGCGGAATGCGCCAAGGAATCACAAACACAAGGCGTGCCCCCTCGACCCGGAAGCGGTGTTCGTCTGGGTGGCGTCGCAAAAAATTGAGTCCAAAATGACTCTCGGCAACGGATATCTCGGCTCTTGCATCGATGAAGAACGTAGCGAAATGCGATACTTGGTGTGAATTGCAGAATCCCGTGAACCATCGAGTCTTTGAACGCAAGTTGCGCCCGAAGCCATTAGGCTAAGGGCACGCCTGCCTGGGTGTCACCAATCGTCGCCCCCAACCTCACtGCCTTGTTGCGTGGAGAAGGGGTGAATGATGGCTTCCCGTGAGCACAGTCTCGCGGTTGGCTGAAAACGTTCTCCGTGCCGGCGTGCAGTGCCGTGACACTTGGTGGTTGAGTTTACCCTCGAGGCCAGTCACGTGTGCTCCCTGTCGGTTCCGGAAGCATGGACCCGTGAGCGGCAAAGACCGCCCTTGATGCGACCTCAGGTCAGGCGGGGCTACCCGCTGAGTTTAAGCATATCAATAAGCG

>Aindica_6x_Autralia_245

GTAACAAgGTTTCCGTAggTGAaCCTGCGGAAGGatCATTGTTGATGCCTCGACCCAGCTAGACCCGCGAATGTGTTTTACAACTCGGGGTGATCGGGTTGCCTAGGCAGCTCGCCTCCCCGACCCGTTGGGGCTCTGGCCACCCTGTGTGGCTCGGTCMCGACACAACAACAAACCCCGGCGCGGAATGCGCCAAGGAATCACAAACACAAGGCGTGCCCCCTCGACCCGGAAGCGGTGTTCGTCTGGGTGGCGTCGCAAAAAATTGAGTCCAAAATGACTCTCGGCAACGGATATCTCGGCTCTTGCATCGATGAAGAACGTAGCGAAATGCGATACTTGGTGTGAATTGCAGAATCCCGTGAACCATCGAGTCTTTGAACGCAAGTTGCGCCCGAAGCCATTAGGCTAAGGGCACGCCTGCCTGGGTGTCACCAATCGTCGCCCCCAACCTCACTGCCTTGTTGCGTGGAGAAGGGGTGAATGATGGCTTCCCGTGAGCACAGTCTCGCGGTTGGCTGAAAACGTTCTCCGTGCCGGCGTGCAGTGCCGTGACACTTGGTGGTTGAGTTTACCCTCGAGGCCAGTCACGTGTGCTCCCTGTCGGTTCCGGAAGCATGGACCCGTGAGCGGCAAAGACCGCCCTTGATGCGACCTCAGGTCAGGCGGGGCTACCCGCTGAGTTTAAGCATATCAATAAGCG

>Amagna

GTAACAAGGTTTCCGTAGGTGAACCTGCGGAAGGATCATTGTTGATGCCTCGACCCAGCTAGACCCGCGAATGCGTTTTACTACCCGGGGTGATCGGGCTGCCTAGGCAGCTCGCCTCCCTGACTCGTTGGGGCTCTGGCCGCCCTGTGTGGCCCGGTCCCGACACAACAACAAACCCCGGCGCGGAATGCGCCAAGGAATAACAATCACAAGGCGTGCCCCCTCGACCCGGAAGCGGTGTTCGTATGGGTGGCGTCGCAAAAAATTGAGTCCAAAATGACTCTCGGCAACGGATATCTCGGCTCtTGCATCGATgAAgAACGTAGCGAAATGCGATACTTGGTGTGAATTGCAGAATCCCGTGAaCCATCGAGTCTTTGAACGCAAGTTGCGCCCGAAGCCATTAGGCTAAGGGCACGCCTGCCTGGGTGTCACCAATCGTCGCCCCCAACCTCACTGCCTYGTTGCGTGGgGAAGGGGTGAATGATGGCTTCCCGTGAGCACrGTCTCGCGGTTGGCTGAAAACGTTCTCCGTGCYGGCGTGCAGCGCCGTGACACTTGGTGGTTGAGTTTACCCTCGAGGCCAGTCACGTGTGCTCCCTGTcGGTTCCGGAAGCATGGACCCGTGAGCGGCAAAGACCGCCCTTGATGCGACCTCAGGTCAGGCGGGGCTACCCGCTGAGTTTAAGCATATCAATAAGCG

>Apluriarticulata_109

GTAACAAGGTTTCCGTAGGTGAACCTGCGGAAGGATCATTGTTGATGCCTCGACCCAGCTAGACCCGCGAATGTGTTTTACTACCCGGGGTGACCGGGCTGCCTAGGCAGCTCGCCTCCCCGACCCGTTGGGGCTCTGGCCACCCTGTGTGGCTCGGTCCCGACACAACAACAAACCCCGGCGCGGAATGCGCCAAGGAATCACAAACACAAGGCGTGCCCCCTCGACCCGGAAGCGGTGTTCGTCTGGGTGGCGTCGCAAAAGAATTGAGTCCAAAATGACTCTCGGCAACGGATATCTCGGCTCTTGCATCGATGAAGAACGTAGCGAAATGCGATACTTGGTGTGAATTGCAGAATCCCGTGAACCATCGAGTCTTTGAACGCAAGTTGCGCCCGAAGCCATTAGGCTAAGGGCACGCCTGCCTGGGTGTCACCAATCGTCGCCCCCAACCTCACTGCCTTGTTGCGTGGAGAAGGGGTGAATGATGGCTTCCCGTGAGCACAGTCTCGCGGTTGGCTGAAAACGTTCTCCGTGCCGGCGTGCAGCGCCGTGACACTTGGTGGTTGAGTTTACCCTCGAGGCCAGTCACGTGTGCTCCCTGTCGGTTCCGGAAGCATGGACCCGTGAGCGGCAAAGACCGCCCTTGATGCGACCTCAGGTCAGGCGGGGCTACCCGCTGAGTTTAAGCATATCAATAAGCG

>Apratensis_55

GTAACAAGGTTTCCGTAGGTGAACCTGCGGAAGGATCATTGTTGATGCCTCAACCCAATTAGACCGGTGAACGTGTTTTACTACCTAGGGGTGGTAGAGCTGCCCAGGTAGCTCTCCAACCTGATCCATCGGGTTCGGACCACCTAGTGTGGCCTGGTCTCGGTGCAACAACAAACCCCGGCGCGGAATGCGTCAAGGAATTCACAATCACAAGGCGTCCCCACTCGACCCGGCAGCGGTGTTCGTATGGGTGGTGTCACAAAAATTGAGTCCAAAATGACTCTCGGCAACGGATATCTCGGCTCTTGCATCGATGAAGAACGTAGCGAAATGCGATACTTGGTGTGAATTGCAGAATCCCGTGAACCATTGAGTCTTTGAACGCAAGTTGCGCCCGAAGCCATTAGGCTAAGGGCACGCCTGCCTGGGTGTCACCAGTTGTTGCCCCAAACCACATTGCCTATGTGCATGGAGAAGGGTGAATGTTGGCTTCCCGTGAGCACTGCTTGCGGTTGGCTGAAAACGTTCTCCGTGTCGGCGTGCAGCATCGTGGCACTTGGTGGTTGAGTTTGCTCTCGAGGCCAGTCATGTGTGCTCCCTGTCGGTTCCGGAAACATGTACCCGTGAGTGGCAATGATCGCTCAAGATGCGACCTCAGGTCAGGCGGGGCTACCCGCTGAGTTTAAGCATATCAATAAGCG

>Apratensis_135

GTAACAAGGTTTCCGTAGGTGAACCTGCGGAAGGATCATTGTTGATGCCTCAACCCAATTAGACCGGTGAACGTGTTTTACTACCTAGGGGTGGTAGAGCTGCCCAGGTAGCTCTCCAACCTGATCCATCGGGTTCGGACCACCTAGTGTGGCCTGGTCTCGGTGCAACAACAAACCCCGGCGCGGAATGCGTCAAGGAATTCACAATCACAAGGCGTCCCCACTCGACCCGGCAGCGGTGTTCGTATGGGTGGTGTCACAAAAATTGAGTCCAAAATGACTCTCGGCAACGGATATCTCGGCTCTTGCATCGATGAAGAACGTAGCGAAATGCGATACTTGGTGTGAATTGCAGAATCCCGTGAACCATTGAGTCTTTGAACGCAAGTTGCGCCCGAAGCCATTAGGCTAAGGGCACGCCTGCCTGGGTGTCACCAGTTGTTGCCCCAAACCACATTGCCTATGTGCATGGAGAAGGGTGAATGTTGGCTTCCCGTGAGCACTGCTTGCGGTTGGCTGAAAACGTTCTCCGTGTCGGCGTGCAGCATCGTGGCACTTGGTGGTTGAGTTTGCTCTCGAGGCCAGTCATGTGTGCTCCCTGTCGGTTCCGGAAACATGTACCCGTGAGTGGCAATGATCGCTCAAGATGCGACCTCAGGTCAGGCGGGGCTACCCGCTGAGTTTAAGCATATCAATAAGCG

>Apratensis_136_A

GTAACAAGGTTTCCGTAGGTGAACCTGCGGAAGGATCATTGTTGATGCCTCAACCCAATTAGACCGGTGAACGTGTTTTACTACCTAGGGGTGGTAGAGCTGCCCAGGTAGCTCTCCAACCTGATCCATCGGGTTCGGACCACCTAGTGTGGCCTGGTCTCGGTGCAACAACAAACCCCGGCGCGGAATGCGTCAAGGAATTCACAATCACAAGGCGTCCCCACTCGACCCGGCAGCGGTGTTCGTATGGGTGGTGTCACAAAAATTGAGTCCAAAATGACTCTCGGCAACGGATATCTCGGCTCTTGCATCGATGAAGAACGTAGCGAAATGCGATACTTGGTGTGAATTGCAGAATCCCGTGAACCATTGAGTCTTTGAACGCAAGTTGCGCCCGAAGCCATTAGGCTAAGGGCACGCCTGCCTGGGTGTCACCAGTTGTTGCCCCAAACCACATTGCCTATGTGCATGGAGAAGGGTGAATGTTGGCTTCCCGTGAGCACTGCTTGCGGTTGGCTGAAAACGTTCTCCGTGTCGGCGTGCAGCATCGTGGCACTTGGTGGTTGAGTTTGCTCTCGAGGCCAGTCATGTGTGCTCCCTGTCGGTTCCGGAAACATGTACCCGTGAGTGGCAATGATCGCTCAAGATGCGACCTCAGGTCAGGCGGGGCTACCCGCTGAGTTTAAGCATATCAATAAGCG

>Apratensis_136_B

GTAACAAGGTTTCCGTAGGTGAACCTGCGGAAGGATCATTGTTGATGCCTCAACCCAGTTAGACCGGCGAACGCGTTTACTACCCGGGGGTGGTAGAGCTGCCCAGGTAGCTCTCCTCCCCGATCCATCGGGTTTGGGCCACCTAGTGTGGCCTGATTTCGGTGCAACAACAACAAACCCCGGCGCGGAATGCGTCAAGGAATTCACAATCACAAGGCGTGCCCCCTCGACCCGGCAACGGTGTTCGTCTGGGTGGCGTCGCAAAAATTGAGTCCAAAATGACTCTCGGCAACGGATATCTCGGCTCTTGCATCGATGAAGAACGTAGCGAAATGCGATACTTGGTGTGAATTGCAGAATCCCGTGAACCATCGAGTCTTTGAACGCAAGTTGCGCCCGAAGCCATTAGGCTAAGGGCACGCTTTCCTGGGTGTCACCAATCGTCGCCCCCAACCACATTGCCTATGTGCATGGAGAAGGGTGAATGCTGGCTTCCCGTGAGCACCGTCTTGCGGTTGGCTGAAAACGTTCTCCGTGTCGGCGCGCAGCATCGTGGCACTTGGTGGTTGAGTTTGCTCTCGAGGCCAGTCATGCGTGCTCCCTGTCGGTTTCGGAAACATGTACCCTGTGCGGCACTGACCGCCAAAGATGCGACCTCAGGTCAGGCGGGGCTACCCGCTGAGTTTAAGCATATCAATAAGCG

>Apratensis_248

GTAACAAGGTTTCCGTAGGTGAACCTGCGGAAGGATCATTGTTGATGCCTCAACCCAATTAGACCGGTGAACGTGTTTTACTACCTAGGGGTGGTAGAGCTGCCCAGGTAGCTCTCCAACCTGATCCATCGGGTTCGGACCACCTAGTGTGGCCTGGTCTCGGTGCAACAACAAACCCCGGCGCGGAATGCGTCAAGGAATTCACAATCACAAGGCGTCCCCACTCGACCCGGCAGCGGTGTTCGTATGGGTGGTGTCACAAAAATTGAGTCCAAAATGACTCTCGGCAACGGATATCTCGGCTCTTGCATCGATGAAGAACGTAGCGAAATGCGATACTTGGTGTGAATTGCAGAATCCCGTGAACCATTGAGTCTTTGAACGCAAGTTGCGCCCGAAGCCATTAGGCTAAGGGCACGCCTGCCTGGGTGTCACCAGTTGTTGCCCCAAACCACATTGCCTATGTGCATGGAGAAGGGTGAATGTTGGCTTCCCGTGAGCACTGCTTGCGGTTGGCTGAAAACGTTCTCCGTGTCGGCGTGCAGCATCGTGGCACTTGGTGGTTGAGTTTGCTCTCGAGGCCAGTCATGTGTGCTCCCTGTCGGTTCCGGAAACATGTACCCGTGAGTGGCAATGATCGCTCAAGATGCGACCTCAGGTCAGGCGGGGCTACCCGCTGAGTTTAAGCATATCAATAAGCG

>Apratensis_251

GTAACAAGGTTTCCGTAGGTGAACCTGCGGAAGGATCATTGTTGATGCCTCAACCCAATTAGACCGGTGAACGTGTTTTACTACCTAGGGGTGGTAGAGCTGCCCAGGTAGCTCTCCAACCTGATCCATCGGGTTCGGACCACCTAGTGTGGCCTGGTCTCGGTGCAACAACAAACCCCGGCGCGGAATGCGTCAAGGAATTCACAATCACAAGGCGTCCCCACTCGACCCGGCAGCGGTGTTCGTATGGGTGGTGTCACAAAAATTGAGTCCAAAATGACTCTCGGCAACGGATATCTCGGCTCTTGCATCGATGAAGAACGTAGCGAAATGCGATACTTGGTGTGAATTGCAGAATCCCGTGAACCATTGAGTCTTTGAACGCAAGTTGCGCCCGAAGCCATTAGGCTAAGGGCACGCCTGCCTGGGTGTCACCAGTTGTTGCCCCAAACCACATTGCCTATGTGCATGGAGAAGGGTGAATGTTGGCTTCCCGTGAGCACTGCTTGCGGTTGGCTGAAAACGTTCTCCGTGTCGGCGTGCAGCATCGTGGCACTTGGTGGTTGAGTTTGCTCTCGAGGCCAGTCATGTGTGCTCCCTGTCGGTTCCGGAAACATGTACCCGTGAGTGGCAATGATCGCTCAAGATGCGACCTCAGGTCAGGCGGGGCTACCCGCTGAGTTTAAGCATATCAATAAGCG

>Apratensis-260

GTAACAAGGTTTCCGTAGGTGAACCTGCGGAAGGATCATTGTTGATGCCTCAACCCAATTAGACCGGTGAACGTGTTTTACTACCTAGGGGTGGTAGAGCTGCCCAGGTAGCTCTCCAACCTGATCCATCGGGTTCGGACCACCTAGTGTGGCCTGGTCTCGGTGCAACAACAAACCCCGGCGCGGAATGCGTCAAGGAATTCACAATCACAAGGCGTCCCCACTCGACCCGGCAGCGGTGTTCGTATGGGTGGTGTCACAAAAATTGAGTCCAAAATGACTCTCGGCAACGGATATCTCGGCTCTTGCATCGATGAAGAACGTAGCGAAATGCGATACTTGGTGTGAATTGCAGAATCCCGTGAACCATCGAGTCTTTGAACGCAAGTTGCGCCCGAAGCCATTAGGCTAAGGGCACGCCTGCCTGGGTGTCACCAGTTGTTGCCCCAAACCACATTGCCTATGTGCATGGAGAAGGGTGAATGTTGGCTTCCCGTGAGCACTGCTTGCGGTTGGCTGAAAACGTTCTCCGTGTCGGCGTGCAGCATCGTGGCACTTGGTGGTTGAGTTTGCTCTCGAGGCCAGTCATGTGTGCTCCCTGTCGGTTCCGGAAACATGTACCCGTGAGTGGCAATGATCGCTCAAGATGCGACCTCAGGTCAGGCGGGGCTACCCGCTGAGTTTAAGCATATCAATAAGCG

>Apratensis_261

GTAACAAGGTTTCCGTAGGTGAACCTGCGGAAGGATCATTGTTGATGCCTCAACCCAATTAGACCGGTGAACGTGTTTTACTACCTAGGGGTGGTAGAGCTGCCCAGGTAGCTCTCCAACCTGATCCATCGGGTTCGGACCACCTAGTGTGGCCTGGTCTCGGTGCAACAACAAACCCCGGCGCGGAATGCGTCAAGGAATTCACAATCACAAGGCGTCCCCACTCGACCCGGCAGCGGTGTTCGTATGGGTGGTGTCACAAAAATTGAGTCCAAAATGACTCTCGGCAACGGATATCTCGGCTCTTGCATCGATGAAGAACGTAGCGAAATGCGATACTTGGTGTGAATTGCAGAATCCCGTGAACCATTGAGTCTTTGAACGCAAGTTGCGCCCGAAGCCATTAGGCTAAGGGCACGCCTGCCTGGGTGTCACCAGTTGTTGCCCCAAACCACATTGCCTATGTGCATGGAGAAGGGTGAATGTTGGCTTCCCGTGAGCACTGCTTGCGGTTGGCTGAAAACGTTCTCCGTGTCGGCGTGCAGCATCGTGGCACTTGGTGGTTGAGTTTGCTCTCGAGGCCAGTCATGTGTGCTCCCTGTCGGTTCCGGAAACATGTACCCGTGAGTGGCAATGATCGCTCAAGATGCGACCTCAGGTCAGGCGGGGCTACCCGCTGAGTTTAAGCATATCAATAAGCG

>Apratensis_293

GTAACAAGGTTTCCGTAGGTGaAcCTGCGGAAGGATCATTGTTGATGCCTCAACCCAATTAGACCGGTGAACGTGTTTTACTACCTAGGGGTGGTAgAGCTGCCCAGGTAGCTCTCCAACCTGATCCATCGGGTTCGGACCACCTAGTGTGGCCTGGTCTCGGTGCAACAACAAACCCCGGCGCGGAATGCGTCAAGGAATTCACAATCACAAGGCGTCCCCaCTCGACtCGGCAGCGGTGTTCGTATGGGTGGTGTCACAAAAATTGAGTCCAAAATGACTCTCGGCAACGGATATCTCGGCTCTtGCATCGATGAAgAACGTAGCGAAATGCGATACTTGGTGTGAATTGCAGAATCCCGTGAACCATCGAGTCTTTGAACGCAAGTTGCGCCCGAAGCCATTAGGCTAAGGGCACGCCTGCCTGGGTGTCACCAGTTGTTGCCCCaAACCACATTGCCTATGTGCATGGAGAAGGGTGAATGTTGGCTTCCCGTGAGCACTGCTtGCGGTTGGCTGAAAACgTTCTCCGTGTCGGCGTGCAGCATCGTGGCACTTGGTGGTTGAGTTTGCTCTCGAGGCCAGTCATGTGTGCTCCCTGTCGGTTCCgGAAACATGTACCCgTGAGTGGCAATGATCGCTCAAGATGCGACCTCAgGTCAGGCGGGGCTACCCGCTGAGTTTAAGCATATCAaTAAGCG

>Apratensis_337

GTAACAAGGTTTCCGTAGGTGAACCTGCGGAAGGATCATTGTTGATGCCTCAACCCAATTAGACCGGTGAACGTGTTTTACTACCTAGGGGTGGTAGAGCTGCCCAGGTAGCTCTCCAACCTGATCCATCGGGTTCGGACCACCTAGTGTGGCCTGGTCTCGGTGCAACAACAAACCCCGGCGCGGAATGCGTCAAGGAATTCACAATCACAAGGCGTCCCCACTCGACCCGGCAGCGGTGTTCGTATGGGTGGTGTCACAAAAATTGAGTCCAAAATGACTCTCGGCAACGGATATCTCGGCTCTTGCATCGATGAAGAACGTAGCGAAATGCGATACTTGGTGTGAATTGCAGAATCCCGTGAACCATcGAGTCTTTGAACGCAAGTTGCGCCCGAAGCCATTAGGCTAAGGGCACGCCTGCCTGGGTGTCACCAGTTGTTGCCCCAAACCACATTGCCTATGTGCATGGAGAAGGGTGAATGTTGGCTTCCCGTGAGCACTGCTTGCGGTTGGCTGAAAACGTTCTCCGTGTCGGCGTGCAGCATCGTGGCACTTGGTGGTTGAGTTTGCTCTCGAGGCCAGTCATGTGTGCTCCCTGTCGGTTCCGGAAACATGTACCCGTGAGTGGCAATGATCGCTCAAGATGCGACCTCAGGTCAGGCGGGGCTACCCGCTGAGTTTAAGCATATCAATAAGCG

>Arostrata

GTAACAAGGTTTCcGTAGGTGAACCTGCGGAAGGATCATTGTTGATGCCTCAATCCAGCTAGACCCGcGAATTCGTTTTACTACCcGGGGCGATCGAGCTACCTCGGCAGCTCGCCTCCCCGAAACGTTGGGGCGTTGCCGCCCCGTGTGGCCTcGTCCCAGCGCAACAACAAACCCCGGTGCGGAATGCGCCAAGGAATTATTAACCGTAcGGCGCGCCCCCTCGACCtGGCAACGGTGTTCGTTTGGGTGGCGTTGcGAAAATCGAGTCTAAAACGACTCTCGGCAACGGATATCTCGGCTCTtGCATCGatgaagaacgtagCGAAaTGCGATACTTGGTGTGAATTGCAGAATCCCGTGAACCATCGAGTCTTTGAACGCAAGTTGCGCCTGAAGCCATTAGGCTAAGGGCATGCCTGCCTGGGTGTCACTaATCGTCGCCCCAACCCAGTGCCTTCGGGCGTGGAGGGGGGTGAATGCTGGCTTCCTGTGAGCATCGTCTCGCTGTTGGCTGAAAACATTCTCTGTGCCGGCGTGCAGCGCTGTGATACATGGTGGTTGAGTTTATTCTCGATGCCTGTCACAGGTGCCCCCTGTCGGTTCTGGAAACATTGACCCGTGAGCGGCATCGATTGCCCTTGATGCGGCCTCAGGTCAGGCGGGGCTACCCGCTGAGTTTAAGCATATCAATAAGCG

>Arudis-23

GTAACAAGGTTTCCGTAGGTGAACCTGCGGAAGGatCATTGTTGATGCCTCAACCCAGTTAGACCCGCGAATGCGTTTTACTACTCGGGGCGATCGGGTTGCCCTGGCAGCTCGCCTCCCCGACCTGTTGGGGCTCTGGCCACCCGGTGTGGCCTGGTCCTGGCACAACAACAAACCCCGGCGCGGAATGCGCCAAGGAATCACATTCACAATGCGTGCCCCCTCGACCCGGCAGCGGTGTTCGTTCGGGTGGTGTCGCAAAAAATCGAGTCCAAAATGACTCTCGGCAACGGATATCTCGGCTCTTGCATCGATGAAGAACGTAGCGAAATGCGATACTTGGTGTGAATTGCAGAATCCCGTGAACCATCGAGTCTTTGAACGCAAGTTGCGCCCGAAGCCATTAGGCTAAGGGCACGCCTGCCTGGGTGTCACCAATCGTCGCCCCCAACCTAACTGCCATGGTGCgTGGGAGAAGGGGTGAATGCTGGCTTCCCGTGAGCACCGTCTTGTGGTTGGCTGAAAACGTTCTCCGTGCCGGCGTGCAGCGCCGTGACACTTGGTGGTTGAGTTTACCCTCGAGGCCAGTCACGTGTGCTCCCTGTCGGTTCCGGAAGCATGGACCCGTGAGCGGCAACGACCGCCCGTGATGCGACCTCAgGTCAGGCGGGGCTACCCGCTGAGTTTAAGCATATCAaTAAGCG

>Arudis-30

GTAACAAGGTTTCCGTAGGTGAACCTGCGGAAGGATCATTGTTGATGCCTCAACCCAGTTAGACCCGCGAATGCGTTTTACTACTCGGGGCGATCGGGTTGCCCTGGCAGCTCGCCTCCCCGACCTGTTGGGGCTCTGGCCACCCGGTGTGGCCTGGTCCTGGCACAACAACAAACCCCGGCGCGGAATGCGCCAAGGAATCACATTCACAATGCGTGCCCCCTCGACCCGGCAGCGGTGTTCGTTCGGGTGGTGTCGCAAAAAATCGAGTCCAAAATGACTCTCGGCAACGGATATCTCGGCTCTTGCATCGATGAAGAACGTAGCGAAATGCGATACTTGGTGTGAATTGCAGAATCCCGTGAACCATCGAGTCTTTGAACGCAAGTTGCGCCCGAAGCCATTAGGCTAAGGGCACGCCTGCCTGGGTGTCACCAATCGTCGCCCCCAACCTAACTGCCATGGTGCGTGGGAGAAGGGGTGAATGCTGGCTTCCCGTGAGCACCGTCTTGTGGTTGGCTGAAAACGTTCTCCGTGCCGGCGTGCAGCGCCGTGACACTTGGTGGTTGAGTTTACCCTCGAGGCCAGTCACGTGTGCTCCCTGTCGGTTCCGGAAGCATGGACCCGTGAGCGGCAACGACCGCCCGTGATGCGACCTCAGGTCAGGCGGGGCTACCCGCTGAGTTTAAGCATATCAATAAGCG

>Arudis-38

GTAACAAGGTTTCCGTAGGTGAACCTGCGGAAGGATCATTGTTGATGCCTCAACCCAGTTAGACCCGCGAATGCGTTTTACTACTCGGGGCGATCGGGTTGCCCTGGCAGCTCGCCTCCCCGACCTGTTGGGGCTCTGGCCACCCGGTGTGGCCTGGTCCTGGCACAACAACAAACCCCGGCGCGGAATGCGCCAAGGAATCACATTCACAATGCGTGCCCCCTCGACCCGGCAGCGGTGTTCGTTCGGGTGGTGTCGCAAAAAATCGAGTCCAAAATGACTCTCGGCAACGGATATCTCGGCTCTTGCATCGATGAAGAACGTAGCGAAATGCGATACTTGGTGTGAATTGCAGAATCCCGTGAACCATCGAGTCTTTGAACGCAAGTTGCGCCCGAAGCCATTAGGCTAAGGGCACGCCTGCCTGGGTGTCACCAATCGTCGCCCCCAACCTAACTGCCATGGTGCGTGGGAGAAGGGGTGAATGCTGGCTTCCCGTGAGCACCGTCTTGTGGTTGGCTGAAAACGTTCTCCGTGCCGGCGTGCAGCGCCGTGACACTTGGTGGTTGAGTTTACCCTCGAGGCCAGTCACGTGTGCTCCCTGTCGGTTCCGGAAGCATGGACCCGTGAGCGGCAACGACCGCCCGTGATGCGACCTCAGGTCAGGCGGGGCTACCCGCTGAGTTTAAGCATATCAATAAGCG

>Arudis-56

GTAACAAGGTTTCCGTAGGTGAACCTGCGGAAGGATCATTGTTGATGCCTCAACCCAGTTAGACCCGCGAATGCGTTTTACTACTCGGGGCGATCGGGTTGCCCTGGCAGCTCGCCTCCCCGACCTGTTGGGGCTCTGGCCACCCGGTGTGGCCTGGTCCTGGCACAACAACAAACCCCGGCGCGGAATGCGCCAAGGAATCACATTCACAATGCGTGCCCCCTCGACCCGGCAGCGGTGTTCGTTCGGGTGGTGTCGCAAAAAATCGAGTCCAAAATGACTCTCGGCAACGGATATCTCGGCTCTTGCATCGATGAAGAACGTAGCGAAATGCGATACTTGGTGTGAATTGCAGAATCCCGTGAACCATCGAGTCTTTGAACGCAAGTTGCGCCCGAAGCCATTAGGCTAAGGGCACGCCTGCCTGGGTGTCACCAATCGTCGCCCCCAACCTAACTGCCATGGTGCGTGGGAGAAGGGGTGAATGCTGGCTTCCCGTGAGCACCGTCTTGTGGTTGGCTGAAAACGTTCTCCGTGCCGGCGTGCAGCGCCGTGACACTTGGTGGTTGAGTTTACCCTCGAGGCCAGTCACGTGTGCTCCCTGTCGGTTCCGGAAGCATGGACCCGTGAGCGGCAACGACCGCCCGTGATGCGACCTCAGGTCAGGCGGGGCTACCCGCTGAGTTTAAGCATATCAATAAGCG

>Arudis-102

GTAACAAGGTTTCCGTAGGTGAACCTGCGGAAGGATCATTGTTGATGCCTCAACCCAGTTAGACCCGCGAATGCGTTTTACTACTCGGGGCGATCGGGTTGCCCTGGCAGCTCGCCTCCCCGACCTGTTGGGGCTCTGGCCACCCGGTGTGGCCTGGTCCTGGCACAACAACAAACCCCGGCGCGGAATGCGCCAAGGAATCACATTCACAATGCGTGCCCCCTCGACCCGGCAGCGGTGTTCGTTCGGGTGGTGTCGCAAAAAATCGAGTCCAAAATGACTCTCGGCAACGGATATCTCGGCTCTTGCATCGATGAAGAACGTAGCGAAATGCGATACTTGGTGTGAATTGCAGAATCCCGTGAACCATCGAGTCTTTGAACGCAAGTTGCGCCCGAAGCCATTAGGCTAAGGGCACGCCTGCCTGGGTGTCACCAATCGTCGCCCCCAACCTAACTGCCATGGTGCGTGGGAGAAGGGGTGAATGCTGGCTTCCCGTGAGCACCGTCTTGTGGTTGGCTGAAAACGTTCTCCGTGCCGGCGTGCAGCGCCGTGACACTTGGTGGTTGAGTTTACCCTCGAGGCCAGTCACGTGTGCTCCCTGTCGGTTCCGGAAGCATGGACCCGTGAGCGGCAACGACCGCCCGTGATGCGACCTCAGGTCAGGCGGGGCTACCCGCTGAGTTTAAGCATATCAATAAGCG

>Arudis-145

GTAACAAGGTTTCCGTAGGTGAACCTGCGGAAGGATCATTGTTGATGCCTCAACCCAGTTAGACCCGCGAATGCGTTTTACTACTCGGGGCGATCGGGTTGCCCTGGCAGCTCGCCTCCCCGACCTGTTGGGGCTCTGGCCACCCGGTGTGGCCTGGTCCTGGCACAACAACAAACCCCGGCGCGGAATGCGCCAAGGAATCACATTCACAATGCGTGCCCCCTCGACCCGGCAGCGGTGTTCGTTCGGGTGGTGTCGCAAAAAATCGAGTCCAAAATGACTCTCGGCAACGGATATCTCGGCTCTTGCATCGATGAAGAACGTAGCGAAATGCGATACTTGGTGTGAATTGCAGAATCCCGTGAACCATCGAGTCTTTGAACGCAAGTTGCGCCCGAAGCCATTAGGCTAAGGGCACGCCTGCCTGGGTGTCACCAATCGTCGCCCCCAACCTAACTGCCATGGTGCGTGGGAGAAGGGGTGAATGCTGGCTTCCCGTGAGCACCGTCTTGTGGTTGGCTGAAAACGTTCTCCGTGCCGGCGTGCAGCGCCGTGACACTTGGTGGTTGAGTTTACCCTCGAGGCCAGTCACGTGTGCTCCCTGTCGGTTCCGGAAGCATGGACCCGTGAGCGGCAACGACCGCCCGTGATGCGACCTCAGGTCAGGCGGGGCTACCCGCTGAGTTTAAGCATATCAATAAGCG

>Arudis-324

GTAACAAGGTTTCCgTAGGTGAACCTGCGGAAGGatCATTGTTGATGCCTCAACCCAGTTAGACCCGCGAATGCGTTTTACTACTCGGGGCGATCGGGTTGCCCTGGCAGCTCGCCTCCCCGACCTGTTGGGGCTCTGGCCACCCGGTGTGGCCTGGTCCTGGCACAACAACAAACCCCGGCGCGGAATGCGCCAAGGAATCACATTCACAATGCGTGCCCCCTCGACCCGGCAGCGGTGTTCGTTCGGGTGGTGTCGCAAAAAATCGAGTCCAAAATGACTCTCGGCAACGGATATCTCGGCTCTTGCATCGATGAAGAACGTAGCGAAATGCGATACTTGGTGTGAATTGCAGAATCCCGTGAACCATCGAGTCTTTGAACGCAAGTTGCGCCCGAAGCCATTAGGCTAAGGGCACGCCTGCCTGGGTGTCACCAATCGTCGCCCCCAACCTAACTGCCATGGTGCGTGGGAGAAGGGGTGAATGCTGGCTTCCCGTGAGCACCGTCTTGTGGTTGGCTGAAAACGTTCTCCGTGCCGGCGTGCAGCGCCGTGACACTTGGTGGTTGAGTTTACCCTCGAGGCCAGTCACGTGTGCTCCCTGTCGGTTCCGGAAGCATGGACCCGTGAGCGGCAACGACCGCCCGTGATGCGACCTCAgGTCAGGCGGGGCTACCCGCTGAGTTTAAGCATATCAaTAAGCG

>Ascabra-11

GTAACAAGGTTTCCGTAGGTGAACCTGCGGAAGGatCATTGTTGATGCCTCGACCCAGCTAGACCTGCGAATGCGTTTTACTACCCGGGGTGATTGGGTTGCCCAGGCAGCTCGCCTCCCCGACCCGCTGGGGCCGTGGCCACTCGGTGTGGCCCGGTCACGGCACAACAACAAACCCCGGCGCGGAATGCGCCAAGGAATCACAATCACAATGCGTGCCCCCTCGACCCGGCAACGGTGTTCGTTCGGGTGGCGTCGCAAAAAATCGAGTCCAAAATGACTCTCGGCAACGGATATCTCGGCTCTTGCATCGATGAAGAACGTAGCGAAATGCGATACTTGGTGTGAATTGCAGAATCCCGTGAACCATCGAGTCTTTGAACGCAAGTTGCGCCCGAAGCCATTAGGCTAAGGGCACGCCTGCCTGGGTGTCACCAATCGTCGCCCCCATCCTCACTGCCTTGGTGTGTGGAGCAGGGTGAATGCTGGCTTCCCGTGAGCACCGTCTTGCGGTTGGCTGAAAACGTTCTCCGTGCCGGCGTGCAGCGTCGTGACACTTGGTGGTTGAGTTTACTCTCGAGGCCAGTCACGTGTGCTCCCTGTCGGTTCCGGAAGCAAGGACCCGTGAGCGGCAACGACCGCCCGTGATGCGACCTCAGGTCAGGCGGGGCTACCCGCTGAGTTTAAGCATATCAATAAGCG

>Ascabra_26

GTAACAAGGTTTCCGTAGGTGAACCTGCGGAAGGATCATTGTTGATGCCTCGACCCAGCTAGACCTGCGAATGCGTTTTACTACCCGGGGTGATTGGGTTGCCCAGGCAGCTCGCCTCCCCGACCCGCTGGGGCCGTGGCCACTCGGTGTGGCCCGGTCACGGCACAACAACAAACCCCGGCGCGGAATGCGCCAAGGAATCACAATCACAATGCGTGCCCCCTCGACCCGGCAACGGTGTTCGTTCGGGTGGCGTCGCAAAAAATCGAGTCCAAAATGACTCTCGGCAACGGATATCTCGGCTCTTGCATCGATGAAGAACGTAGCGAAATGCGATACTTGGTGTGAATTGCAGAATCCCGTGAACCATCGAGTCTTTGAACGCAAGTTGCGCCCGAAGCCATTAGGCTAAGGGCACGCCTGCCTGGGTGTCACCAATCGTCGCCCCCATCCTCACTGCCTTGGTGTGTGGAGCAGGGTGAATGCTGGCTTCCCGTGAGCACCGTCTTGCGGTTGGCTGAAAACGTTCTCCGTGCCGGCGTGCAGCGTCGTGACACTTGGTGGTTGAGTTTACTCTCGAGGCCAGTCACGTGTGCTCCCTGTCGGTTCCGGAAGCAAGGACCCGTGAGCGGCAACGACCGCCCGTGATGCGACCTCAGGTCAGGCGGGGCTACCCGCTGAGTTTAAGCATATCAATAAGCG

>Ascabra-58

GTAACAAGGTTTCCGTAGGTGAACCTGCGGAAGGatCATTGTTGATGCCTCGACCCAGCTAGACCCGCGAATGCGTTTTACTACCCGGGGTGATCGGGTTGCCCAGGCAGCTCGCCTCCCCGACCCGCTGGGGCCGTGGCCACTCGGTGTGGCCCGGTCACGGCACAACAACAAACCCCGGCGCGGAATGCGCCAAGGAATCACAATCACAATGCGTGCCCCCTCGACCCGGCAACGGTGTTCGTTCGGGTGGCGTCGCAAAAAATCGAGTCCAAAATGACTCTCGGCAACGGATATCTCGGCTCTTGCATCGATGAAGAACGTAGCGAAATGCGATACTTGGTGTGAATTGCAGAATCCCGTGAACCATCGAGTCTTTGAACGCAAGTTGCGCCCGAAGCCATTAGGCTAAGGGCACGCCTGCCTGGGTGTCACCAATCGTCGCCCCCATCCTCACTGCCTTGGTGTGTGGAGCAGGGTGAATGCTGGCTTCCCGTGAGCACCGTCTTGCGGTTGGCTGAAAACGTTCTCCGTGCCGGCGTGCAGCGTCGTGACACTTGGTGGTTGAGTTTACTCTCGAGGCCAGTCACGTGTGCTCCCTGTCGGTTCCGGAAGCAAGGACCCGTGAGCGGCAACGACCGCCCGTGATGCGACCTCAgGTCAGGCGGGGCTACCCGCTGAGTTTAAGCATATCAaTAAGCG

>Ascabra-110

GTAACAAGGTTTCCGTAGGTGAACCTGCGGAAGGATCATTGTTGATGCCTCGACCCAGCTAGACCCGCGAATGCGTTTTACTACCCGGGGTGATCGGGTTGCCCAGGCAGCTCGCCTCCCCGACCCGCTGGGGCTGTGGCCACTCGGTGTGGCCCGGTCACGGCACAACAACAAACCCCGGCGCGGAATGCGCCAAGGAATCACAATCACAATGCGTGCCCCCTCGACCCGGCAACGGTGTTCGTTCGGGTGGCGTCGCAAAAAATCGAGTCCAAAATGACTCTCGGCAACGGATATCTCGGCTCTTGCATCGATGAAGAACGTAGCGAAATGCGATACTTGGTGTGAATTGCAGAATCCCGTGAACCATCGAGTCTTTGAACGCAAGTTGCGCCCGAAGCCATTAGGCTAAGGGCACGCCTGCCTGGGTGTCACCAATCGTCGCCCCCATCCTCACTGCCTTGGTGTGTGGAGCAGGGTGAATGCTGGCTTCCCGTGAGCACCGTCTTGCGGTTGGCTGAAAACGTTCTCCGTGCCGGCGTGCAGCGTCGTGACACTTGGTGGTTGAGTTTACTCTCGAGGCCAGTCACGTGTGCTCCCTGTCGGTTCCGGAAGCAAGGACCCGTGAGCGGCAACGACCGCCCGTGATGCGACCTCAGGTCAGGCGGGGCTACCCGCTGAGTTTAAGCATATCAATAAGCG

>Ascabra-147

GTAACAAGGTTTCCGTAGGTGAACCTGCGGAAGGatCATTGTTGATGCCTCGACCCAGCTAGACCCGCGAATGCGTTTTACTACCCGGGGTGATCGGGTTGCCCAGGCAGCTCGCCTCCCCGACCCGCTGGGGCCGTGGCCACTCGGTGTGGCCCGGTCACGGCACAACAACAAACCCCGGCGCGGAATGCGCCAAGGAATCACAATCACAATGCGTGCCCCCTCGACCCGGCAACGGTGTTCGTTCGGGTGGCGTCGCAAAAAATCGAGTCCAAAATGACTCTCGGCAACGGATATCTCGGCTCTTGCATCGATGAAGAACGTAGCGAAATGCGATACTTGGTGTGAATTGCAGAATCCCGTGAACCATCGAGTCTTTGAACGCAAGTTGCGCCCGAAGCCATTAGGCTAAGGGCATGCCTGCCTGGGTGTCACCAATCGTCGCCCCCATCCTCACTGCCTTGGTGTGTGGAGCAGGGTGAATGCTGGCTTCCCGTGAGCACCGTCTTGCGGTTGGCTGAAAACGTTCTCCGTGCCGGCGTGCAGCGTCGTGACACTTGGTGGTTGAGTTTACTCTCGAGGCCAGTCACGTGTGCTCCCTGTCGGTTCCGGAAGCAAGGACCCGTGAGCGGCAACGACCGCCCGTGATGCGACCTCAGGTCAGGCGGGGCTACCCGCTGAGTTTAAGCATATCAATAAGCG

>Ascabra_221

GTAACAAGGTTTCCGTAGGTGAacCTGCGGAAGGatCATTGTTGATGCCTCGACCCaGCTAGACCCGCGAATGCGTTTTACTACCCGGGGTGATCGGGTTGCCCAGGCAGCTCGCCTCCCCGACCCGCTGGGGCCGTGGCCACTCGGTGTGGCCCGGTCACGGCACAACAACAAACCCCGGCGCGGAATGCGCCAAGGAATCACAATCACAATGCGTGCCCCCTCGACCCGGCAACGGTGTTCGTTCGGGTGGCGTCGCAAAAAATCGAGTCCAAAATGACTCTCGGCAACGGATATCTCGGCTCTTGCATCGATGAAGAACGTAGCGAAATGCGATACTTGGTGTGAATTGCAGAATCCCGTGAACCATCGAGTCTTTGAACGCAAGTTGCGCCCGAAGCCATTAGGCTAAGGGCACGCCTGCCTGGGTGTCACCAATCGTCGCCCCCATCCTCACTGCCTTGGTGTGTGGAGCAGGGTGAATGCTGGCTTCCCGTGAGCACCGTCTTGCGGTTGGCTGAAAACGTTCTCCGTGCCGGCGTGCAGCGTCGTGACACTTGGTGGTTGAGTTTACTCTCGAGGCCAGTCACGTGTGCTCCCTGTCGGTTCCGGAAGCAAGGACCCGTGAGCGGCAACGACCGCCCGTGATGCGACCTCAgGTCAGGCGGGGCTACCCGCTGAGTTTAAGCATATCAaTAAGCG

>Ascabra-344

GTAACAAGGTTTCCGTAGGTGAACCTGCGGAAGGatCATTGTTGATGCCTCGACCCAGCTAGACCCGCGAATGCGTTTTACTACCCGGGGTGATCGGGTTGCCCAGGCAGCTCGCCTCCCCGACCCGCTGGGGCCGTGGCCACTCGGTGTGGCCCGGTCACGGCACAACAACAAACCCCGGCGCGGAATGCGCCAAGGAATCACAATCACAATGCGTGCCCCCTCGACCCGGCAACGGTGTTCGTTCGGGTGGCGTCGCAAAAAATCGAGTCCAAAATGACTCTCGGCAACGGATATCTCGGCTCTTGCATCGATGAAGAACGTAGCGAAATGCGATACTTGGTGTGAATTGCAGAATCCCGTGAACCATCGAGTCTTTGAACGCAAGTTGCGCCCGAAGCCATTAGGCTAAGGGCACGCCTGCCTGGGTGTCACCAATCGTCGCCCCCATCCTCACTGCCTTGGTGTGTGGAGCAGGGTGAATGCTGGCTTCCCGTGAGCACCGTCTTGCGGTTGGCTGAAAACGTTCTCCGTGCCGGCGTGCAGCGTCGTGACACTTGGTGGTTGAGTTTACTCTCGAGGCCAGTCACGTGTGCTCCCTGTCGGTTCCGGAAGCAAGGACCCGTGAGCGGCAACGACCGCCCGTGATGCGACCTCAGGTCAGGCGGGGCTACCCGCTGAGTTTAAGCATATCAaTAAGCG

>Ascabra_345

GTAACAAGGTTTCCgTAGGTGAACCTGCGGAAGGAtCATTGTTGATGCCTCGACCCAGCTAGACCCGCGAATGCGTTTTACTACCCGGGGTGATCGGGTTGCCCAGGCAGCTCGCCTCCCCGACCCGCTGGGGCCGTGGCCACTCGGTGTGGCCCGGTCACGGCACAACAACAAACCCCGGCGCGGAATGCGCCAAGGAATCACAATCACAATGCGTGCCCCCTCGACCCGGCAACGGTGTTCGTTCGGGTGGCGTCGCAAAAAATCGAGTCCAAAATGACTCTCGGCAACGGATATCTCGGCTCTTGCATCGATGAAGAACGTAGCGAAATGCGATACTTGGTGTGAATTGCAGAATCCCGTGAACCATCGAGTCTTTGAACGCAAGTTGCGCCCGAAGCCATTAGGCTAAGGGCACGCCTGCCTGGGTGTCACCAATCGTCGCCCCCATCCTCACTGCCTTGGTGTGTGGAGCAGGGTGAATGCTGGCTTCCCGTGAGCACCGTCTTGCGGTTGGCTGAAAACGTTCTCCGTGCCGGCGTGCAGCGTCGTGACACTTGGTGGTTGAGTTTACTCTCGAGGCCAGTCACGTGTGCTCCCTGTCGGTTCCGGAAGCAAGGACCCGTGAGCGGCAACGACCGCCCGTGATGCGACCTCAGGTCAGGCGGGGCTACCCGCTGAGTTTAAGCATATCAaTAAGCG

>Atambacoundensis-60

GTAACAAGGTTTCCGTAGGTGAACCTGCGGAAGGATCATTGTTGATGCCTCAATCCAGCTAGACCCGCGAATTCGTTTTACTACCCGGGGCGATGGAGCTACCTCGGCAGCTCGCCTCCCCGAAACGTTGGGGCGTTGCCGCCCCGTGTGGCCTCGTCCCAGCGCAACAACAAACCCCGGTGCGGAATGCGCCAAGGAATTCACAACCGTAAGGCGCGCCCCCTCGACCTGGCAACGGTGTTCGTTTGGGTGGCGTTGCGAAAATGGAGTCTAAAATGACTCTCGGCAACGGATATCTCGGCTCTTGCATCGATGAAGAACGTAGCGAAATGCGATACTTGGTGTGAATTGCAGAATCCCGTGAACCATCGAGTCTTTGAACGCAAGTTGCGCCTGAAGCCATTAGGCTAAGGGCATGCCTGCCTGGGTGTCACTAATCGTCGCCCCAACCCAGTGCCTTCGGGCGTGGAGGGGGGTGAATGCTGGCTTCCTGTGAGCATCGTCTCGCTGTTGGCTGAAAACATTCTCTGTGCCGGCGTGCAGCGCTGTGATGCTTGGTGGTTGAGTTTATTCTCGATGCCTGTCACAGGTGCCCCCTGTCGGTTCTGGAAACAATGACCCATGGGCGGCATCGATCGCCCTTGATGCGACCTCAGGTCAGGCGGGGCTACCCGCTGAGTTTAAGCATATCAATAAGCG

>Aselloi_186

GTAACAAGGTTTCCGTAGGTGAaCCTGCGGAAGGatCATTGTTGATGCCTCAACCCAGTTAGACCGGCGAACGCGTTTTACTACCCAGGGGTGGTAGATCTGCCTAGGTAGCTCTCCACCCTGATCCATCGGGGTCGGGCCTCCTAGTGTGGCCTGGTCTCGGTGCAACAACAAACCCCGGCGCGGAATGCGTCAAGGAATTCACAATCAGAAGGCGTCCCCCCTCGACCCTGCAACGGTGTTCGTCTGGGTGGCGTCGCAAAAATTGAGTCCAAAATGACTCTCGGCAACGGATATCTCGGCTCTTGCATCGATGAAGAACGTAGCGAAATGCGATACTTGGTGTGAATTGCAGAATCCCGTGAACCATCGAGTCTTTGAACGCAAGTTGCGCCTGAAGCCATTAGGCTAAGGGCACGCCTGCCTGGGTGTCACCAATCGTCGCCCCTAACCACATTGCCTATGTGCATGGAGAAGGGTGAATGCTGGCTTCCCGTGAGCACCCGTCTTGCGGTTGGCTGAAAACGTTCTCCGTGTCGGCACGCAGCATCGTGGCACTTGGTGGTTGAGTTTGCTCTCGAGGCCAGTCACGCATGCTCCCTGTCGGTTGTGGAATCATGTACCCGTGAGCGGCAATAACCGCCCAAGATGCGACCTCAGGTCAGGCGGGGCTACCCGCTGAGTTTAAGCATATCAATAAGCG

>Aselloi_334

GTAACAAGGTTTCCgTAgGTGAACCTGCGGAAGGAtCATTGTTGATGCCTCAACCCAGTTAGACCGGCGAACGCGTTTTACTACCCAGGGGTGGTAGATCTGCCTAGGTAGCTCTCCACCCTGATCCATCGGGGTCGGGCCTCCTAGTGTGGCCTGGTCTCGGTGCAACAACAAACCCCGGCGCGGAATGCGTCAAGGAATTCACAATCAGAAGGCGTCCCCCCTCGACCCTGCAACGGTGTTCGTCTGGGTGGCGTCGCAAAAATTGAGTCCAAAATGACTCTCGGCAACGGATATCTCGGCTCTTGCATCGATGAAGAACGTAGCGAAATGCGATACTTGGTGTGAATTGCAGAATCCCGTGAACCATCGAGTCTTTGAACGCAAGTTGCGCCTGAAGCCATTAGGCTAAGGGCACGCCTGCCTGGGTGTCACCAATCGTCGCCCCtAACCACATTGCCTATGTGCATGGAGAAGGGTGAATGCTGGCTTCCCGTGAGCACCCGTCTTGCGGTTGGCTGAAAACGTTCTCCGTGTCGGCACGCAGCATCGTGGCACTTGGTGGTTGAGTTTGCTCTCGAGGCCAGTCACGCATGCTCCCTGTCGGTTGTGGAATCATGTACCCGTGAGCGGCAATAACCGCCCAAGATGCGACCTCAGGTCAGGCGGGGCTAcCCGCTGAGTTTAAGCATATCAaTAAGCG

>Aselloi_347

GTAACAAGGTTTCCgTAgGTGAACCTGCGGAAGGatCATTGTTGATGCCTCAACCCAGTTAGACCGGCGAACGCGTTTTACTACCCAGGGGTGGTAGATCTGCCTAGGTAGCTCTCCACCCTGATCCATCGGGGTCGGGCCTCCTAGTGTGGCCTGGTCTCGGTGCAACAACAAACCCCGGCGCGGAATGCGTCAAGGAATTCACAATCAGAAGGCGTCCCCCCTCGACCCTGCAACGGTGTTCGTCTGGGTGGCGTCGCAAAAATTGAGTCCAAAATGACTCTCGGCAACGGATATCTCGGCTCTTGCATCGATGAAGAACGTAGCGAAATGCGATACTTGGTGTGAATTGCAGAATCCCGTGAACCATCGAGTTTTTGAACGCAAGTTGCGCCTGAAGCCATTAGGCTAAGGGCACGCCTGCCTGGGTGTCACCAATCGTCGCCCCCAACCACATTGCCTATGTGCATGGAGAAGGGTGAATGCTGGCTTCCCGTGAGCACCCGTCTTGCGGTTGGCTGAAAACGTTCTCCGTGTCGGCGCGCAGCATCGTGGCACTTGGTGGTTGAGTTTGCTCTCGAGGCCAGTCACGCATGCTCCCTGTCGGTTGTGGAATCATGTACCCGTGAGCGGCAATAACCGCCCAAGATGCGACCTCAGGTCAGGCGGGGCTACCCGCTGAGTTTAAGCATATCAaTAAGCG

>Asensitiva_28

GTAACAAGGTTTCCGTAGGTGAACCTGCGGAAGGATCATTGTTGATGCCTCAACCCAGTTAGACCGGCGAACGCGTTTTACTACCCGGGGGTGGTAGAGCTGCCCAGGTAGCTCTCCTCCCCGATCCATCGGGTTTGGGCCACCTAGTGTGGCCTGATTTCGGTGCAACAACAACAAACCCCGGCGCGGAATGCGTCAAGGAATTCACAATCACAAGGCGTGCCCCCTCAACCCGGCAACGGTGTTCGTCTGGGTGGCGTCGCAAAAATTGAGTCCAAAATGACTCTCGGCAACGGATATCTCGGCTCTTGCATCGATGAAGAACGTAGCGAAATGCGATACTTGGTGTGAATTGCAGAATCCCGTGAACCATCGAGTCTTTGAACGCAAGTTGCGCCCGAAGCCATTAGGCTAAGGGCACGCCTGCCTGGGTGTCACCAATCGTCGCCCCCAACCACATTGCCTATGTGCATGGAGAAGGGTGAATGCTGGCTTCCCGTGAGCACCGTCTTGCGGTTGGCTGAAAACGTTCTCCGTGTCGGCGCGCAGCATCGTGGCACTTGGTGGTTGAGTTTGCTCTCGAGGCCAGTCATGCGTGCTCCCTGTCGGTTCCGGAAACATGTACCCTGCGCGGCACTGACCGCCAAAGATGCGACCTCAGGTCAGGCGGGGCTACCCGCTGAGTTTAAGCATATCAATAAGCG

>Asensitiva_32

GTAACAAGGTTTCcGTAGGTGAACcTGCGGAAGGATCATTGTTGATGCCTCAACCCAGTTAGACCGGCGAACGTGTTTTACTACCcGGGGGTGGTAGAGCTGCCCAGGTAGCTCTCCTCCCCGATCCATCGGGTTTGGGCCACCTAGTGTGGCCTGAtTTCGGTGCAACAACAACAAACCCCGGCGCGGAaTGCGTCAAGGAATTCACAATCACAAGGCGTGCCCCCTCGACCCGGCAACGGTGTTcGTCTGGGTGGCGTCGCAAAAATTGAGTCCAAAATGACTCTCGGCAACGGATATCTCGGCTCTTGCATCGATGAAGAACGTAGCGAAATGCGATACTTGGTGTGAATTGCAGAATCCCGTGAACCATCGAGTCTTTGAACGCAAGTTGCGCCCGAAGCCATTAGGCTAAGGGCACGCCTGCCTGGGTGTCACCAATCGTCGCCCCCAACCACATTGCCTATGTGCATGGAGAAGGGTGAATGCTGGCTTCCCGTGAGCACCGTCTTGCGGTTGGCTGAAAACGTTCTCCGTGTCGGCGCGCAGCATCGTGGCACTTGGTGGTTGAGTTTGCTCTCGAGGCCAGTCATGCGTGCTCCCTGTCGGTTCCGGAAACATGTACCCTGCGCGGCACTGACCGCCAAAGATGCGACCTCAGGTCAGGCGGGGCTACCCGCtGAGTTTAAGCAtATCAATAAGCG

>Asensitiva_33

GTAACAAGGTTTCcGTAGGTGAACcTGCGGAAGGATCATTGTTGATGCCTCAACCCAGTTAGACCGGCGAACGTGTTTTACTACCCGGGGGTGGTAGAGCTGCCCAGGTAGCTCTCCTCCCCGATCCATCGGGTTTGGGCCACCTAGTGTGGCCTGATTTCGGTGCAACAACAACAAACCCCGGCGCGGAATGCGTCAAGGAATTCACAATCACAAGGCGTGCCCCCTCGACCCGGCAACGGTGTTCGTCTGGGTGGCGTCGCAAAAATTGAGTCCAAAATGACTCTCGGCAACGGATATCTCGGCTCTTGCATCGATGAAGAACGTAGCGAAATGCGATACTTGGTGTGAATTGCAGAATCCCGTGAACCATCGAGTCTTTGAACGCAAGTTGCGCCCGAAGCCATTAGGCTAAGGGCACGCCTGCCTGGGTGTCACCAATCGTCGCCCCCAACCACATTGCCTATGTGCATGGAGAAGGGTGAATGCTGGCTTCCCGTGAGCACCGTCTTGCGGTTGGCTGAAAACGTTCTCCGTGTCGGCGCGCAGCATCGTGGCACTTGGTGGTTGAGTTTGCTCTCGAGGCCAGTCATGCGTGCTCCCTGTCGGTTCCGGAAACATGTACCCTGCGCGGCACTGACCGCCAAAGATGCGACCTCAGGTCAGGCGGGGCTACCCGCTGAGTTTAAGCATATCAATAAGCG

>Asensitiva_34

GTAACAAGGTTTCCGTAGGTGAacCTGCGGAAGGaTCATTGTTGATGCCTCAACCCAGTTAGACCGGCGAACGTGTTTTACTACCCGGGGGTGGTAGAGCTGCCCAGGTAGCTCTCCTCCCCGATCCATCGGGTTTGGGCCACCTAGTGTGGCCTGATTTCGGTGCAACAACAACAAACCCCGGCGCGGAATGCGTCAAGGAATTCACAATCACAAGGCGTGCCCCCTCGACCCGGCAACGGTGTTCGTCTGGGTGGCGTCGCAAAAATTGAGTCCAAAATGACTCTCGGCAACGGATATCTCGGCTCTTGCATCGATGAAGAACGTAGCGAAATGCGATACTTGGTGTGAATTGCAGAATCCCGTGAACCATCGAGTCTTTGAACGCAAGTTGCGCCCGAAGCCATTAGGCTAAGGGCACGCCTGCCTGGGTGTCACCAATCGTCGCCCCCAACCACATTGCCTATGTGCATGGAGAAGGGTGAATGCTGGCTTCCCGTGAGCACCGTCTTGCGGTTGGCTGAAAACGTTCTCCGTGTCGGCGCGCAGCATCGTGGCACTTGGTGGTTGAGTTTGCTCTCGAGGCCAGTCATGCGTGCTCCCTGTCGGTTCCGGAAACATGTACCCTGTGCGGCACTGACCGCCAAAGATGCGACCTCAGGTCAGGCGGGGCTACCCGCTGAGTTTAAGCATATCAaTAAGCG

>Asensitiva_35

GTAACAAGGTTTCCGTAgGTGAaCCTGCGGAAGGatCATTGTTGATGCCTCAACCCAGTTAGACCGGCGAACGTGTTTTACTACCCGGGGGTGGTAGAGCTGCCCAGGTAGCTCTCCTCCCCGATCCATCGGGTTTGGGCCACCTAGTGTGGCCTGATTTCGGTGCAACAACAACAAACCCCGGCGCGGAATGCGTCAAGGAATTCACAATcacaagGCGTGCCCCCTCGACCCGGCAACGGTGTTCGTCTGGGTGGCGTCGCAAAAATTGAGTCCAAAATGACTCTCGGCAACGGATATCTCGGCTCTTGCATCGATGAAGAACGTAGCGAAATGCGATACTTGGTGTGAATTGCAGAATCCCGTGAACCATCGAGTCTTTGAACGCAAGTTGCGCCCGAAGCCATTAGGCTAAGGGCACGCCTGCCTGGGTGTCACCAATCGTCGCCCCCAACCACATTGCCTATGTGCATGGAGAAGGGTGAATGCTGGCTTCCCGTGAGCACCGTCTTGCGGTTGGCTGAAAACGTTCTCCGTGTCGGCGCGCAGCATCGTGGCACTTGGTGGTTGAGTTTGCTCTCGAGGCCAGTCATGCGTGCTCCCTGTCGGTTCCGGAAACATGTACCCTGTGCGGCACTGACCGCCaAAGATGCGACCTCAgGTCAgGCGGGGCTACCCGCTgAGTTTAAGCATATCAaTAAGCG

>Asensitiva_250

GTAACAAGGTTTccgTAGGTGAAccTGCGGAAGGATCATTGTTGATGCCTCAACCCAGTTAGACCGGCGAACGCGTTTACTACCCGGGGGTGGTAGAGCTGCCCAGGTAGTTCTCcTCCCCGATCCATCGGGTTTGGGCCACCTAGTGTGGCCTGATTTCGGTGCAACAACAACAAACCCCGGCGCGGAaTGCGTCAAGGAATTCACAATCACAAGGCGTGCCCCCTCGACCCGGCAACGGTGTTCGTCTGGGTGGCGTCGCAAAAATTGAGTCCAAAATGACTCTCGGCAACGGATATCTCGGCTCTTGCATCGATGAAGAACGTAGCGAAATGCGATACTTGGTGTGAATTGCAGAATCCCGTGAACCATCGAGTCTTTGAACGCAAGTTGCGCCCGAAGCCATTAGGCTAAGGGCACGCCTGCCTGGGTGTCACCAATCGTCGCCCCCAACCACAttgCCTATGTGCATGGAGAAGGGTGAATGCTGGCTTCCCGTGAGCACCGTCTTGCGGTTGGCTGAAAACGTTCTCCGTGTTGGCGCGCAGCATCGTGGCACTTGGTGGTTGAGTTTGCTCTCGAGGCCAGTCATGCGTGCTCCCTGTCGGTTTCGGAAACATGTACCCTGCGCGGCACTGACCGCCAAAGATGCGACCTCAGGTCAGGCGGGGCTACCCGCtGAGTTTAAGCATATCAATAAGCG

>Asensitiva_252

GTAACAAGGTTTccgTAGGTGAAcCTGCGGAAGGatCATTGTTGATGCCTCAACCCAGTTAGACCGGCGAACGCGTTTACTACCCGGGGGTGGTAGAGCTGCCCAGGTAGCTCTCCTCCCCGATCCATCGGGTTTGGGCCACCTAGTGTGGCCTGATTTCGGTGCAACAACAACAAACCCCGGCGCGgAATGCGTCAAGGAATTCACAATCACAAGGCGTGCCCCCTCGACCCGGCaACGGTGTTCGTCTGGGTGGCGTCGCAAAAATTGAGTCCAAAATGACTCTCGGCAACGGATATCTCGGCTCTTGCATCGATgAAgAACGTAGCGAAATGCGATACTTGGTGTGAATTGCAGAATCCCGTGAACCATCGAGTCTTTGAACGCAAGTTGCGCCCGAAGCCATTAGGCTAAGGGCACGCCTGCCTGGGTGTCACCAATCGTCGCCCCCAACCACATTGCCTATGTGCATGGAGAAGGGTGAATGCTGGCTTCCCGTGAGCACCGTCTTGCGGTTGGCTGAAAACGTTCTCCGTGTCGGCGCGCAGCATCGTGGCACTTGGTGGTTGAGTTTGCTCTCGAGGCCAGTCATGCGTGCTCCCTGTCGGTTTCGGAAACATGTACCCTGtGCGGCACTGACCGCCaAAGATGCGACCTCAgGTCAgGCGGGGCTACCCGCTGAGTTTAAGCATATCAaTAAGCG

>Asensitiva_253

GTAACAAGGTTTCCGTAGGTGAACCTGCGGAAGGATCATTGTTGATGCCTCAACTCAGTTAGACCGGTGAACGCGTTTACTACCCGGGGGTGGTAGAGCTGCCCAGGTAGCTCTCCTCCCCGATCCATCGGGTTTGGGCCACCTAGTGTGGCCTGATTTCGGTGCAACAACAACAAACCCCGGCGCGGAATGCGTCAAGGAATTCACAATCACAAGGCGTGCCCCCTCGACCCGGCAACGGTGTTCGTCTGGGTGGCGTCGCAAAAATTGAGTCCAAAATGACTCTCGGCAACGGATATCTCGGCTCTTGCATCGATGAAGAACGTAGCGAAATGCGATACTTGGTGTGAATTGCAGAATCCCGTGAACCATCGAGTCTTTGAACGCAAGTTGCGCCCGAAGCCATTAGGCTAAGGGCACGCCTGCCTGGGTGTCACCAATCGTCGCCCCCAACCACATTGCCTATGTGCATGGAGAAGGGTGAATGCTGGCTTCCCGTGAGCACCGTCTTGCGGTTGGCTGAAAACGTTCTCCGTGCCGGCGCGCAGCATCGTGGCACTTGGTGGTTGAGTTTGCTCTCGAGGCCAGTCATGCGTGCTCCCTGTCGGTTCCGGAAACATGTACCCTGCGCGGCACTGACCGCCAAAGATGCGACCTCAGGTCAGGCGGGGCTACCCGCTGAGTTTAAGCATATCAATAAGCG

>Asensitiva_255

GTAACAAGGTtTCCGTAGGTGAacCTGCGGAAGGatCATTGTTGATGCCTCAACCCAGTTAGACCGGCGAACGCGTTTTACTACCCGGGGGTGGTAGAGCTGCCCAGGTAGCTCTCCTCCCCGATCCATCGGGTTTGGGCCACCTAGTGTGGCCTGATTTCGGTGCAACAACAACAAACCCCGGCGCGGAATGCGTCAAGGAATTCACAATCATAAGGCGTGCCCCTCgACCCGGCAACGGTgTTCGTCTGGGTGTCGTCGCAAAAATTGAGTCCAAAATGACTCTCGGCAACGGATATCTCGGCTCTTGCATCGATGAAgAACGTAGCGAAATGCGATACTTGGTGTGAATTGCAgAATCCCGTGAACCATCGAGTCTTTGAACGCAAGTTGCGCCCGAAGCCATTAGGCCAAGGGCACGCCTGCCTGGGTGTCACCAATCGTCGCCCCCAACCAACATTGCCTATgTGCATGGAgAAGGGTGAATGCTGGCTTCCCGTGAGCACCGTCTTGtGGTTGGCTGAAAACGTTCTCCGTGTCGGCGCGCAgCATCGTGGCACTTGGTGGTTGAGTTTGCTCTCGAGGCCAGTCATGCgTGCTCCCTGTCGGTTCCGGAAACATGTACCCTGCGCGGCACTGACCGCCAAAgATGCGACCTCAGGTCAGGCGGGGCTACCCGCTGAGTTTAAGCATATCAaTAAGCG

>Asensitiva_256

GTAACAAGGTTTCcGTAGGTGAACcTGCGGAAGGATCATTGTTGATGCCTCAACCCAGTTAGACCGGCGAACGTGTTTTACTACCCGGGGGTGGTAGAGCTGCCCAGGTAGCTCTCCTCCCCGATCCATCGGGTTTGGGCCACCTAGTGTGGCCTGATTTCGGTGCAACAACAACAAACCCCGGCGCGGAaTGCGTCAAGGAATTCACAATCACAAGGCGTGCCCCCTCGACCCGGCAACGGTGTTCGTCTGGGTGGCGTCGCAAAAATTGAGTCCAAAATGACTCTCGGCAACGGATATCTCGGCTCTTGCATCGATGAAGAACGTAGCGAAATGCGATACTTGGTGTGAATTGCAGAATCCCGTGAACCATCGAGTCTTTGAACGCAAGTTGCGCCCGAAGCCATTAGGCTAAGGGCACGCCTGCCTGGGTGTCACCAATCGTCGCCCCCAACCACAttgCCTATGTGCATGGAGAAGGGTGAATGCTGGCTTCCCGTGAGCACCGTCTTGCGGTTGGCTGAAAACGTTCTCCGTGTCGGCGCGCAGCATCGTGGCACTTGGTGGTTGAGTTTGCTCTCGAGGCCAGTCATGCGTGCTCCCTGTCGGTTCCGGAAACATGTACCCTGCGCGGCACTGACCGCCAAAGATGCGACCTCAGGTCAGGCGGGGCTACCCGCtGAGTTTAAGCATATCAATAAGCG

>Asensistiva_262

GTAACAAGGTTTCCGTAgGTGAacCTGCGGAAGGaTCATTGTTGATGCCTCAACCCAGTTAGACCGGCGAACGCGTTTACTACCCGGGGGTGGTAGAGCTGCCCAGGTAGCTCTCCTCCCCGATCCATCGGGTTTGGGCCACCTAGTGTGGCCTGATTTCGGTGCAACAACAACAAACCCCGGCGCGGAATGCGTCAAGGAATTCACAATCACAAGGCGTGCCCCCTCGACCCGGCAACGGTGTTCGTCTGGGTGGCGTCGCAAAAATTGAGTCCAAAATGACTCTCGGCAACGGATATCTCGGCTCTTGCATCGATGAAGAACGTAGCGAAATGCGATACTTGGTGTGAATTGCAGAATCCCGTGAACCATCGAGTCTTTGAACGCAAGTTGCGCCCGAAGCCATTAGGCTAAGGGCACGCCTGCCTGGGTGTCACCAATCGTCGCCCCCAACCACATTGCCTATGTGCATGGAGAAGGGTGAATGCTGGCTTCCCGTGAGCACCGTCTTGCGGTTGGCTGAAAACGTTCTCCGTGTCGGCGCGCAGCATCGTGGCACTTGGTGGTTGAGTTTGCTCTCGAGGCCAGTCATGCGTGCTCCCTGTCGGTTTCGGAAACATGTACCCTGTGCGGCACTGACCGCCAAAGATGCGACCTCAGGTCAGGCGGGGCTACCCGCTGAGTTTAAGCATATCAaTAAGCG

>Asensitiva_268

GTAACAAGGTTTCcGTAGGTGAACCTGCGGAAGGaTCATTGTTGATGCCTCAACCCAGTTAGACCGGTGAACGCGTTTACTACCCGGGGGTGGTAGAGCTGCCCAGGTAGCTCTCCTCCCCGATCCATCGGGTTTGGGCCACCTAGTGTGGCCTGATTTCGGTGCAACAACAACAAACCCCGGCGCGGAATGCGTCAAGGAATTCACAATCACAAGGCGTGCCCCCTCGACCCGGGAACGGTGATCGTGTGGGTGGCGTcGCAAAAATTGAGTCCAAAATGACTCTCGGCAACGGATATCTCGGCTCTTGCATCGATGAAGAACGTAGCGAAATGCGATACTTGGTGTGAATTGCAGAATCCCGTGAACCATCGAGTCTTTGAACGCAAGTTGCGCCCGAAGCCATTAGGCTAAGGGCACGCCTGCCTGGGTGTCACCAATCGTCGCCCCCAACCACATTGCCTATGTGCATGGAGAAGGGTGAATGCTGGCTTCCCGTGAGCACCGTCTTGCGGTTGGCTGAAAACGTTCTCCGTGTCGGCGCGCAGCATCGTGGCACTTGGTGGTTGAGTTTGCTCTCGAGGCCAGTCATGCGTGCTCCCTGTCGGTTCCGGAAACATGTACCCTGCGCGGCACTGACCGCCaAAGATGCGACCTCAgGTCAgGCGGGGCTACCCGCTGAGTTTAAGCATATCaaTAAGCG

>Asensitiva_271

GTAACAAGGTTTCcGTAGGTGAACcTGCGGAAGGATCATTGTTGATGCCTCAACCCAGTTAGACCGGCGAACGTGTTTTACTACCCGGGGGTGGTAGAGCTGCCCAGGTAGCTCTCCTCCCCGATCCATCGGGTTTGGGCCACCTAGTGTGGCCTGATTTCGGTGCAACAACAACAAACCCCGGCGCGGAATGCGTCAAGGAATTCACAATCACAAGGCGTGCCCCCTCGACCCGGCAACGGTGTTCGTCTGGGTGGCGTCGCAAAAATTGAGTCCAAAATGACTCTCGGCAACGGATATCTCGGCTCTTGCATCGATGAAGAACGTAGCGAAATGCGATACTTGGTGTGAATTGCAGAATCCCGTGAACCATCGAGTCTTTGAACGCAAGTTGCGCCCGAAGCCATTAGGCTAAGGGCACGCCTGCCTGGGTGTCACCAATCGTCGCCCCCAACCACATTGCCTATGTGCATGGAGAAGGGTGAATGCTGGCTTCCCGTGAGCACCGTCTTGCGGTTGGCTGAAAACGTTCTCCGTGTCGGCGCGCAGCATCGTGGCACTTGGTGGTTGAGTTTGCTCTCGAGGCCAGTCATGCGTGCTCCCTGTCGGTTCCGGAAACATGTACCCTGCGCGGCACTGACCGCCAAAGATGCGACCTCAGGTCAGGCGGGGCTACCCGCTGAGTTTAAGCATATCAATAAGCG

>Asensitiva_274

GTAACAAGGTTTCcGTAGGTGAACcTGCGGAAGGATCATTGTTGATGCCTCAACCCAGTTAGACCGGCGAACGCGTTTTACTACCCGGGGGTGGTAGAGCTGCCCAGGTAGCTCTCCTCCCCGATCCATCGGGTTTGGGCCACCTAGTGTGGCCTGAtTTCGGTGCAACAACAACAAACCCCGGCGCGGAaTGCGTCAAGGAATTCACAATCATAAGGCGTGCCCCCTCGACCCGGCAACGGTGTTCGTCTGGGTGGCGTCGCAAAAATTGAGTCCAAAATGACTCTCGGCAACGGATATCTCGGCTCTTGCATCGATGAAGAACGTAGCGAAATGCGATACTTGGTGTGAATTGCAGAATCCCGTGAACCATCGAGTCTTTGAACGCAAGTTGCGCCCGAAGCCATTAGGCCAAGGGCACGCCTGCCTGGGTGTCACCAATCGTCGCCCCCAACCACAttgCCTATGTGCATGGAGAAGGGTGAATGCTGGCTTCCCGTGAGCACCGTCTTGCGGTTGGCTGAAAACGTTCTCCGTGTCGGCGCGCAGCATCGTGGCACTTGGTGGTTGAGTTTGCTCTCGAGGCCAGTCATGCGTGCTCCCTGTCGGTTCCGGAAACATGTACCCTGCGCGGCACTGACCGCCAAAGATGCGACCTCAGGTCAGGCGGGGCTACCCGCtGAGTTTAAGCATATCAaTAAGCG

>Asensitiva_275

GTAACAAGGTTTCCGTAGgTGAacCTGCGGAAGGatCATTGTTGATGCCTCAACCCAGTTAGACCGGCGAACGCGTTTACTACCCGGGGGTGGTAGAGCTGCCCAGGTAGCTCTCCTCCCCGATCCATCGGGTTTGGGCCACCTAGTGTGGCCTGATTTCGGTGCAACAACAACAAACCCCGGCGCGGAATGCGTCAAGGAATTCACAATCACAAGGCGTGCCCCCTCGACCCGGCAACGGTGTTCGTCTGGGTGGCGTCGCAAAAATTGAGTCCAAAATGACTCTCGGCAACGGATATCTCGGCTCTTGCATCGATGAAGAACGTAGCGAAATGCGATACTTGGTGTGAATTGCAGAATCCCGTGAACCATCGAGTCTTTGAACGCAAGTTGCGCCCGAAGCCATTAGGCTAAGGGCACGCCTGCCTGGGTGTCACCAATCGTCGCCCCCAACCACATTGCCTATGTGCATGGAGAAGGGTGAATGTTGGCTTCCCGTGAGCACCGTCTTGCGGTTGGCTGAAAACGTTCTCCGTGTCGGCGCGCAGCATCGTGGCACTTGGTGGTTGAGTTTGCTCTCGAGGCCAGTCATGCGTGCTCCCTGTCGGTTTCGGAAACATGTACCCTGCGCGGCACTGACCGCCAAAGATGCGACCTCAGGTCAgGCGGGGCTACCCGCTGAGTTTAAGCATATCAaTAAGCG

>Asensitiva_292

GTAACAAGGTTTCCGTAGGTGAACCTGCGGAAGGATCATTGTTGATGCCTCAACCCAGTTAGACCGGCGAACGCGTTTTACTACCCGGGGGTGGTAGAGCTGCCCAGGTAGCTCTCCTCCCCGATCCATCGGGTTTGGGCCACCTAGTGTGGCCTGATTTCGGTGCAACAACAACAAACCCCGGCGCGGAATGCGTCAAGGAATTCACAATCACAAGGCGTGCCCCCTCAACCCGGCAACGGTGTTCGTCTGGGTGGCGTCGCAAAAATTGAGTCCAAAATGACTCTCGGCAACGGATATCTCGGCTCTTGCATCGATGAAGAACGTAGCGAAATGCGATACTTGGTGTGAATTGCAGAATCCCGTGAACCATCGAGTCTTTGAACGCAAGTTGCGCCCGAAGCCATTAGGCTAAGGGCACGCCTGCCTGGGTGTCACCAATCGTCGCCCCCAACCACATTGCCTATGTGCATGGAGAAGGGTGAATGCTGGCTTCCCGTGAGCACCGTCTTGCGGTTGGCTGAAAACGTTCTCCGTGTCGGCGCGCAGCATCGTGGCACTTGGTGGTTGAGTTTGCTCTCGAGGCCAGTCATGCGTGCTCCCTGTCGGTTCCGGAAACATGTACCCTGCGCGGCACTGACCGCCAAAGATGCGACCTCAGGTCAGGCGGGGCTACCCGCTGAGTTTAAGCATATCAATAAGCG

>Asensitiva_314

GTAACAAGGTTTCCGTAGGTGAACCTGCGGAAGGATCATTGTTGATGCCTCAACCCAGTTAGACCGGCGAACGCGTTTTACTACCCGGGGGTGGTAGAGCTGCCCAGGTAGCTCTCCTCCCCGATCCATCGGGTTTGGGCCACCTAGTGTGGCCTGATTTCGGTGCAACAACAACAAACCCCGGCGCGGAATGCGTCAAGGAATTCACAATCACAAGGCGTGCCCCCTCAACCCGGCAACGGTGTTCGTCTGGGTGGCGTCGCAAAAATTGAGTCCAAAATGACTCTCGGCAACGGATATCTCGGCTCTTGCATCGATGAAGAACGTAGCGAAATGCGATACTTGGTGTGAATTGCAGAATCCCGTGAACCATCGAGTCTTTGAACGCAAGTTGCGCCCGAAGCCATTAGGCTAAGGGCACGCCTGCCTGGGTGTCACCAATCGTCGCCCCCAACCACATTGCCTATGTGCATGGAGAAGGGTGAATGCTGGCTTCCCGTGAGCACCGTCTTGCGGTTGGCTGAAAACGTTCTCCGTGTCGGCGCGCAGCATCGTGGCACTTGGTGGTTGAGTTTGCTCTCGAGGCCAGTCATGCGTGCTCCCTGTCGGTTCCGGAAACATGTACCCTGCGCGGCACTGACCGCCAAAGATGCGACCTCAGGTCAGGCGGGGCTACCCGCTGAGTTTAAGCATATCAATAAGCG

>Asensitiva_325

GTAACAAGGTTTCCgTAgGTGAaCCTGCGGAAGGatCATTGTTGATGCCTCAACCCAGTTAGACCGGCGAACGTGTTTTACTACCCGGGGGTGGTAGAGCTGCCCAGGTAGCTCTCCTCCCCGATCCATCGGGTTTGGGCCACCTAGTGTGGCCTGATTTCGGTGCAACAACAACAAACCCCGGCGCGGAATGCGTCAAGGAATTCACAATCACAAGGCGTGCCCCCTCGACCCGGCAACGGTGTTCGTCTGGGTGGCGTCGCAAAAATTGAGTCCAAAATGACTCTCGGCAACGGATATCTCGGCTCTTGCATCGATGAAGAACGTAGCGAAATGCGATACTTGGTGTGAATTGCAGAATCCCGTGAACCATCGAGTCTTTGAACGCAAGTTGCGCCCGAAGCCATTAGGCTAAGGGCACGCCTGCCTGGGTGTCACCAATCGTCGCCCCCAACCACATTGCCTATGTGCATGGAGAAGGGTGAATGCTGGCTTCCCGTGAGCACCGTCTTGCGGTTGGCTGAAAACGTTCTCCGTGTCGGCGCGCAGCATCGTGGCACTTGGTGGTTGAGTTTGCTCTCGAGGCCAGTCATGCGTGCTCCCTGTCGGTTCCGGAAACATGTACCCTGCGCGGCACTGACCGCCAAAGATGCGACCTCAGGTCAGGCGGGGCTACCCGCTGAGTTTAAGCATATCAaTAAGCG

>Asensitiva_327

GTAACAAGGTTTCCgTAGGTGAACCTGCGGAAGGAtCATTGTTGATGCCTCAACCCAGTTAGACCGGCGAACGCGTTTTACTACCCGGGGGTGGTAGAGCTGCCCAGGTAGCTCTCCTCCCCGATCCATCGGGTTTGGGCCACCTAGTGTGGCCTGATTTCGGTGCAACAACAACAAACCCCGGCGCGGAATGCGTCAAGGAATTCACAATCACAAGGCGTGCCCCCTCGACCCGGCAACGGTGTTCGTCTGGGTGGCGTCGCAAAAATTGAGTCCAAAATGACTCTCGGCAACGGATATCTCGGCTCTTGCATCGATGAAGAACGTAGCGAAATGCGATACTTGGTGTGAATTGCAGAATCCCGTGAACCATCGAGTCTTTGAACGCAAGTTGCGCCCGAAGCCATTAGGCTAAGGGCACGCCTGCCTGGGTGTCACCAATCGTCGCCCCCAACCACATTGCCTATGTGCATGGAGAAGGGTGAATGCTGGCTTCCCGTGAGCACCGTCTTGCGGTTGGCTGAAAACGTTCTCCGTGTCGGCGCGCAGCATCGTGGCACTTGGTGGTTGAGTTTGCTCTCGAGGCCAGTCATGCGTGCTCCCTGTCGGTTCCGGAAACATGTACCCTGCGCGGCACTGACCGCCAAAGATGCGACCTCAGGTCAGGCGGGGCTACCCGCTGAGTTTAAGCATATCAaTAAGCG

>Asensitiva_329

GTAACAAGGTTTCCGTAgGTGAACCTGCGGAAGGatCATTGTTGATGCCTCAACCCAGTTAGACCGGCGAACGCGTTTACTACCCGGGGGTGGTAGAGCTGCCCAGGTAGCTCTCCTCCCCGATCCATCGGGTTTGGGCCACCTAGTGTGGCCTGATTTCGGTGCAACAACAACAAACCCCGGCGCGGAATGCGTCAAGGAATTCACAATCACAAGGCGTGCCCCCTCGACCCGGCAACGGTGTTCGTCTGGGTGGCGTCGCAAAAATTGAGTCCAAAATGACTCTCGGCAACGGATATCTCGGCTCTTGCATCGATGAAGAACGTAGCGAAATGCGATACTTGGTGTGAATTGCAGAATCCCGTGAACCATCGAGTCTTTGAACGCAAGTTGCGCCCGAAGCCATTAGGCTAAGGGCACGCCTGCCTGGGTGTCACCAATCGTCGCCCCCAACCACATTGCCTATGTGCATGGAGAAGGGTGAATGCTGGCTTCCCGTGAGCACCGTCTTGCGGTTGGCTGAAAACGTTCTCCGTGTCGGCGCGCAGCATCGTGGCACTTGGTGGTTGAGTTTGCTCTCGAGGCCAGTCATGCGTGCTCCCTGTCGGTTTCGGAAACATGTACCCTGTGCGGCACTGACCGCCAAAGATGCGACCTCAGGTCAGGCGGGGCTACCCGCTGAGTTTAAGCATATCAaTAAGCG

>Asensitiva_336

GTAACAAGGTTTCCGTAGGTGAACCTGCGGAAGGATCATTGTTGATGCCTCAACCCAGTTAGACCGGCGAACGCGTTTACTACCCGGGGGTGGTAGAGCTGCCCAGGTAGCTCTCCTCCCCGATCCATCGGGTTTGGGCCACCTAGTGTGGCCTGATTTCGGTGCAACAACAACAAACCCCGGCGCGGAATGCGTCAAGGAATTCACAATCACAAGGCGTGCCCCCTCGACCCGGCAACGGTGTTCGTCTGGGTGGCGTCGCAAAAATTGAGTCCAAAATGACTCTCGGCAACGGATATCTCGGCTCTTGCATCGATGAAGAACGTAGCGAAATGCGATACTTGGTGTGAATTGCAGAATCCCGTGAACCATCGAGTCTTTGAACGCAAGTTGCGCCCGAAGCCATTAGGCTAAGGGCACGCCTGCCTGGGTGTCACCAATCGTCGCCCCCAACCGCATTGCCTATGTGCATGGAGAAGGGTGAATGCTGGCTTCCCGTGAGCACCGTCTTGcGGTTGGCTGAAAACGTTTTCCGTGTCGGCGCGCAGCATCGTGGCACTTGGTGGTTGAGTTTGCTCTCGAGGCCAGTCATGCGTGCTCCCTGTCGGTTTCGGAAACATGTACCCCGTGCGGCACTGACCGCCAAAGATGCGACCTCAgGTCAgGCGGGGCTACCCGCTGAGTTTAAGCATATCAATAAGCG

>Asensitiva_341

GTAACAAGGTTTCCgTAGGTGAACCTGCGGAAGGatCATTGTTGATGCCTCAACCCAGTTAGACCGGCGAACGTGTTTTACTACCCGGGGGTGGTAGAGCTGCCCAGGTAGCTCTCCTCCCCGATCCATCGGGTTTGGGCCACCTAGTGTGGCCTGATTTCGGTGCAACAACAACAAACCCCGGCGCGGAATGCGTCAAGGAATTCACAATCACAAGGCGTGCCCCCTCGACCCGGCAACGGTGTTCGTCTGGGTGGCGTCGCAAAAATTGAGTCCAAAATGACTCTCGGCAACGGATATCTCGGCTCTTGCATCGATGAAGAACGTAGCGAAATGCGATACTTGGTGTGAATTGCAGAATCCCGTGAACCATCGAGTCTTTGAACGCAAGTTGCGCCCGAAGCCATTAGGCTAAGGGCACGCCTGCCTGGGTGTCACCAATCGTCGCCCCCAACCACATTGCCTATGTGCATGGAGAAGGGTGAATGCTGGCTTCCCGTGAGCACCGTCTTGCGGTTGGCTGAAAACGTTCTCCGTGTCGGCGCGCAGCATCGTGGCACTTGGTGGTTGAGTTTGCTCTCGAGGCCAGTCATGCGTGCTCCCTGTCGGTTCCGGAAACATGTACCCTGCGCGGCACTGACCGCCaAAGATGCGACCTCAgGTCAgGCGGGGCTACCCGCTGAGTTTAAGCATATCAaTAAGCG

>Asensitiva_343

GTAACAAGGTTTCCgTAGGTGAACCTGCGGAAGGatCATTGTTGATGCCTCAACCCAGTTAGACCGGCGAACGCGTTTACTACCCGGGGGTGGTAGAGCTGCCCAGGTAGCTCTCCTCCCCGATCCATCGGGTTTGGGCCACCTAGTGTGGCCTGATTTCGGTGCAACAACAACAAACCCCGGCGCGGAATGCGTCAAGGAATTCACAATCACAAGGCGTGCCCCCTCGACCCGGCAACGGTGTTCGTCTGGGTGGCGTCGCAAAAATTGAGTCCAAAATGACTCTCGGCAACGGATATCTCGGCTCTTGCATCGATGAAGAACGTAGCGAAATGCGATACTTGGTGTGAATTGCAGAATCCCGTGAACCATCGAGTCTTTGAACGCAAGTTGCGCCCGAAGCCATTAGGCTAAGGGCACGCCTGCCTGGGTGTCACCAATCGTCGCCCCCAACCGCATTGCCTATGTGCATGGAGAAGGGTGAATGCTGGCTTCCCGTGAGCACCGTCTTGCGGTTGGCTGAAAACGTTTTCCGTGTCGGCGCGCAGCATCGTGGCACTTGGTGGTTGAGTTTGCTCTCGAGGCCAGTCATGCGTGCTCCCTGTCGGTTTCGGAAACATGTACCCCGTGCGGCACTGACCGCCAAAGATGCGACCTCAGGTCAGGCGGGGCTACCCGCTGAGTTTAAGCATATCAATAAGCG

>Asensitiva_348

GTAACAAGGTTTCCgTAgGTGAACCTGCGGAAGGatCATTGTTGATGCCTCAACCCAGTTAGACCGGCGAACGCGTTTTACTACCCGGGGGTGGTAGAGCTGCCCAGGTAGCTCTCCTCCCCGATCCATCGGGTTTGGGCCACCTAGTGTGGCCTGATTTCGGTGCAACAACAACAAACCCCGGCGCGGAATGCGTCAAGGAATTCACAATCACAAGGCGTGCCCCCTCGACCCGGCAACGGTGTTCGTCTGGGTGGCGTCGCAAAAATTGAGTCCAAAATGACTCTCGGCAACGGATATCTCGGCTCTTGCATCGATGAAGAACGTAGCGAAATGCGATACTTGGTGTGAATTGCAGAATCCCGTGAACCATCGAGTCTTTGAACGCAAGTTGCGCCCGAAGCCATTAGGCTAAGGGCACGCCTGCCTGGGTGTCACCAATCGTCGCCCCCAACCACATTGCCTATGTGCATGGAGAAGGGTGAATGCTGGCTTCCCGTGAGCACCGTCTTGCGGTTGGCTGAAAACGTTCTCCGTGTCGGCGCGCAGCATCGTGGCACTTGGTGGTTGAGTTTGCTCTCGAGGCCAGTCATGCGTGCTCCCTGTCGGTTCCGGAAACATGTACCCTGCGCGGCACTGACCGCCAAAGATGCGACCTCAGGTCAGGCGGGGCTACCCGCTGAGTTTAAGCATATCAaTAAGCG

>Asensitiva_354

GTAACAAGGTTTCCGTAGGTGAACCTGCGGAAGGatCATTGTTGATGCCTCAACCCAGTTAGACCGGCGAACGCGTTTTACTACCCGGGGGTGGTAGAGCTGCCCAGGTAGCTCTCCTCCCCGATCCATCGGGTTTGGGCCACCTAGTGTGGCCTGATTTCGGTGCAACAACAACAAACCCCGGCGCGGAATGCGTCAAGGAATTCACAATCATAAGGCGTGCCCCTCGACCCGGCAACGGTGTTCGTCTGGGTGTCGTCGCAAAAATTGAGTCCAAAATGACTCTCGGCAACGGATATCTCGGCTCTTGCATCGATGAAGAACGTAGCGAAATGCGATACTTGGTGTGAATTGCAGAATCCCGTGAACCATCGAGTCTTTGAACGCAAGTTGCGCCCGAAGCCATTAGGCCAAGGGCACGCCTGCCTGGGTGTCACCAATCGTCGCCCCCAACCAACATTGCCtATgTGCATGGAGAAGGGTGAATGCTGGCTTCCCGTGAGCACCGTCTTGTGGTTGGCTGAAAACGTTCTCCGTGTCGGCgCGCAgCAtCGTGGCACTTGGTGGTTGAGTTTGCTCTCGAGGCCAGTCATGCGTGCTCCCTGTCGGTTCCGGAAACATGTACCCTGCGCGGCACTGACCGCCAAAgATGCGACCTCAGGTCAGGCGGGGCTACCCGCTGAGTTTAAGCATATCAaTAAGCG

>Asensitiva_355

GTAACAAGGTTTCCgTAgGTGAACCTGCGGAAGGatCATTGTTGATGCCTCAACCCAGTTAGACCGGCGAACGCGTTTACTACCCGGGGGTGGTAGAGCTGCCCAGGTAGCTCTCCTCCCCGATCCATCGGGTTTGGGCCACCTAGTGTGGCCTGATTTCGGTGCAACAACAACAAACCCCGGCGCGGAATGCGTCAAGGAATTCACAATCACAAGGCGTGCCCCCTCGACCCGGCAACGGTGTTCGTCTGGGTGGCGTCGCAAAAATTGAGTCCAAAATGACTCTCGGCAACGGATATCTCGGCTCTTGCATCGATGAAGAACGTAGCGAAATGCGATACTTGGTGTGAATTGCAGAATCCCGTGAACCATCGAGTCTTTGAACGCAAGTTGCGCCCGAAGCCATTAGGCTAAGGGCACGCCTGCCTGGGTGTCACCAATCGTCGCCCCCAACCGCATTGCCTATGTGCATGGAGAAGGGTGAATGCTGGCTTCCCGTGAGCACCGTCTTGCGGTTGGCTGAAAACGTTTTCCGTGTCGGCGCGCAGCATCGTGGCACTTGGTGGTTGAGTTTGCTCTCGAGGCCAGTCATGCGTGCTCCCTGTCGGTTTCGGAAACATGTACCCCGTGCGGCACTGACCGCCAAAGATGCGACCTCAGGTCAGGCGGGGCTACCCGCTGAGTTTAAGCATATCAaTAAGCG

>Asenstiva_361

GTAACAAGGtTTCCgTAGGTGAACCTGCGGAAGGATCATTGTTGATGCCTCAACCCAGTTAGACCGGCGAACGCGTTTTACTACCCGGGGGTGGTAGAGCTGCCCAGGTAGCTCTCCTCCCCGATCCATCGGGTTTGGGCCACCTAGTGTGGCCTGATTTCGGTGCAACAACAACAAACCCCGGCGCGGAATGCGTCAAGGAATTCACAATCACAAGGCGTGCCCCCTCAACCCGGCAACGGTGTTCGTCTGGGTGGCGTCGCAAAAATTGAGTCCAAAATGACTCTCGGCAACGGATATCTCGGCTCTTGCATCGATGAAGAACGTAGCGAAATGCGATACTTGGTGTGAATTGCAGAATCCCGTGAACCATCGAGTCTTTGAACGCAAGTTGCGCCCGAAGCCATTAGGCTAAGGGCACGCCTGCCTGGGTGTCACCAATCGTCGCCCCCAACCACATTGCCTATGTGCATGGAGAAGGGTGAATGCTGGCTTCCCGTGAGCACCGTCTTGCGGTTGGCTGAAAACGTTCTCCGTGTCGGCGCGCAGCATCGTGGCACTTGGTGGTTGAGTTTGCTCTCGAGGCCAGTCATGCGTGCTCCCTGTCGGTTCCGGAAACATGTACCCTGCGCGGCACTGACCGCCAAAGATGCGACCTCAGGTCAgGCGGGGCTACCCGCTGAGTTTAAGCATATCAaTAAGCG

>Asp_328

GTAACAAGGTTTCCgTAGGTGAACCTGCGGAAGGATCATTGTTGATGCCTCAACCCAGATAGACCGGCGAACGCGTTTTACTACCCAGGGGTGGTAGAGCTGCCCAGGTAGCTCTCCACCCTGATCCATCGGGTTCGGGCCACCTAGTGTGGCCTGGTCTTGGTGCAAcAACAAACCCCGGCGCGGAATGCGTCAAGGAATTCACAATCACAAGGCGTCCCCCTCGACCCGGcAGCGGTGTTCGTCCGGGTGGCGTCGCAAAAATTGAGTCCAAAATGACTCTCGGCAACGGATATCTCGGCTCTTGCATCGATGAAGAACGTAGCGAAATGCGATACTTGGTGTGAATTGCAGAATCCCGTGAACCATCGAGTCTTTGAACGCAAGTTGCGCCCGAAGCCATTAGGCTAAGGGCACGCCTGCCTGGGTGTCACCAATCGTCGCCCCCAACCACATTGCCTATGTGCATGGATtAGGGTGAATGCTGGCTTCCCGTGAGCACCGTCTTGCGGTTGGCTGAAAATGTTCTCCGTGTCGGCGCGCAGCATCGTGGCACTTGGTGGTTGAGTTTGCTCTCGAGGCCAGTCACGCGTGCTCCCTGTTGGTTCCGGAAACATGTACCCGTGAGCGGCAAGGACCGCCCAAGATGCGACCTCAGGTCAGGCGGGGCTACCCGCtGagtttaaGCATATCAaTAAGCG

>Asp_353

GTAACAAGGTTTCCgTAGGTGAACCTGCGGAAGGatCATTGTTGATGCCTCGACCCAGCTAGACCTGCGAATGCGTTTTACTACCCGGGGCGATCGGGTTGCCCAGGCAGCTCGCCTCCCCGACCTGTTGGGGCTCTGGCCACTCGGTGTGGCCTGGTCCTGGCACAACAACAAACCCCGGCGCGGAATGCGCCAAGGAATCACATTCACAATGTGCGCCCCCTCGACCCGGCAACGGTGTTCGTTCGGGTGGTGTCGCAAAAAATCGAGTCCAAAATGACTCTCGGCAACGGATATCTCGGCTCTTGCATCGATGAAGAACGTAGCGAAATGCGATACTTGGTGTGAATTGCAGAATCCCGTGAACCATCGAGTCTTTGAACGCAAGTTGCGCCCGAAGCCATTAGGCTAAGGGCACGCCTGCCTGGGTGTCACCAATCGTCGCCCCCAAACTAACTGCCATGGTGCGTGGAGAAGGGGTGAATGCTGGCTTCCCGTGAGCACCGTCTTGCGGTTGGCTGAAAACGTTCTCCGTGCCGGCgTGCAGCGCCgTGACACTtGGTGGTTGAGTTTACCCTCGAGGCCaGTCACGTGTGCTCCCTGTCGGTTCCGGAAGCATGGACCCGTTGGCGGCAACgACCGCCCGTGATGCGACCTCAGGTCAGGCGGGGCTACCCGCTgAGTTTAAGCATATCAaTAAgCG

>Avirginica_63

GTAACAAGGTTTCCGTAGGTGAACCTGCGGAAGGATCATTGTTGATGCCTTGACCCAGCTAGACCTGCGAATGCGTTTTACTACCCGGGGCGATCGGGTTGCCCAGGCAGCTCGCCTCCCCGACCTGTTGGGGCTCTGGCCACTCGGTGTGGCCTGGTCCTGGCACAACAACAAACCCCGGCGCGGAATGCGCCAAGGAATCACATTCACAATGTGCGCCCCCTCGACCCGGCAACGGTGTTCGTTCGGGTGGTGTCGCAAAAAATCGAGTCCAAAATGACTCTCGGCAACGGATATCTCGGCTCTTGCATCGATGAAGAACGTAGCGAAATGCGATACTTGGTGTGAATTGCAGAATCCCGTGAACCATCGAGTCTTTGAACGCAAGTTGCGCCCGAAGCCATTAGGCTAAGGGCACGCCTGCCTGGGTGTCACCAATCGTCGCCCCCAAACTAACTGCCATGGTGCGTGGAGAAGGGGTGAATGCTGGCTTCCCGTGAGCACCGTCTTGCGGTTGGCTGAAAACGTTCTCCGTGCCGGCGTGCAGCGCCGTGACACTTGGTGGTTGAGTTTACCCTCGAGGCCAGTCACGTGTGCTCCCTGTCGGTTCCGGAAGCATGGACCCGTTGGCGGCAACGACCGCCCGTGATGCGACCTCAGGTCAGGCGGGGCTACCCGCTGAGTTTAAGCATATCAATAAGCG
