# Supplementary figures and images for: Naturally occurring variations in the nod-independent model legume Aeschynomene evenia and relatives: a resource for nodulation genetics
Source: BMC Plant Biol. 2018 Apr 3;18:54. doi: 10.1186/s12870-018-1260-2 (PMC5883870; doi:10.1186/s12870-018-1260-2)

## Slide 1
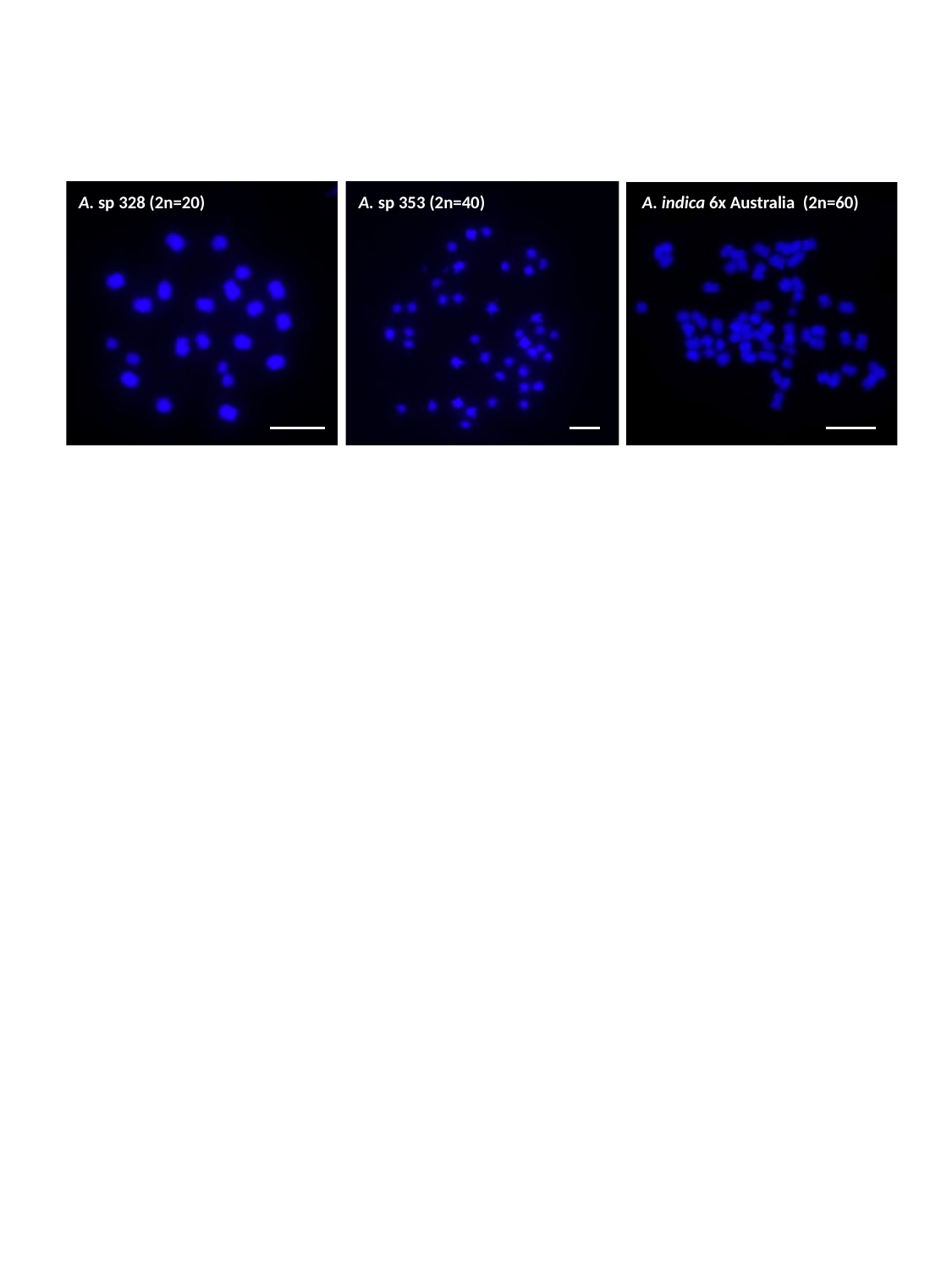

A. sp 328 (2n=20)
A. sp 353 (2n=40)
A. indica 6x Australia (2n=60)

Supplement: Supplementary file 5 — Figure S1. Chromosome numbers in new Aeschynomene taxa. Root tip metaphase chromosomes stained in blue with DAPI (4′,6-diamidino-2-phenylindole). Chromosome numbers are indicated in brackets. Scale bars: 5 μm. (PPTX 1008 kb) [file 12870_2018_1260_MOESM5_ESM.pptx]
