# Supplementary material for: Naturally occurring variations in the nod-independent model legume Aeschynomene evenia and relatives: a resource for nodulation genetics
Source: BMC Plant Biol. 2018 Apr 3;18:54. doi: 10.1186/s12870-018-1260-2 (PMC5883870; doi:10.1186/s12870-018-1260-2)
Supplement: Supplementary file 6 — Figure S2. Schematic representation of the different steps of the genotyping process from marker selection to data treatment. (PPTX 64 kb) [file 12870_2018_1260_MOESM6_ESM.pptx]

## Slide 1
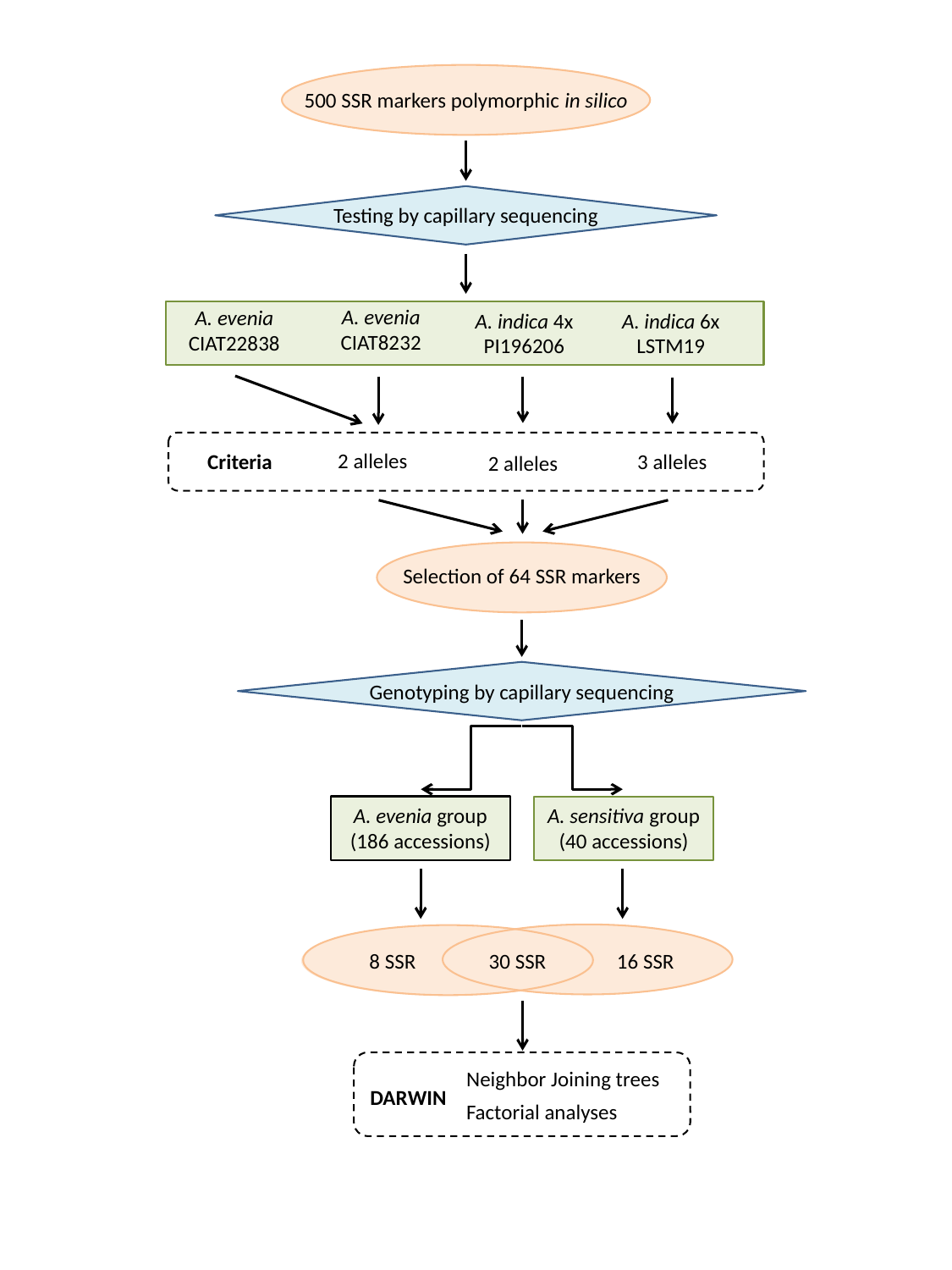

500 SSR markers polymorphic in silico
Testing by capillary sequencing
A. evenia CIAT8232
A. evenia CIAT22838
A. indica 4x PI196206
A. indica 6x LSTM19
2 alleles
3 alleles
Criteria
2 alleles
Selection of 64 SSR markers
Genotyping by capillary sequencing
A. sensitiva group
(40 accessions)
A. evenia group
(186 accessions)
8 SSR
30 SSR
16 SSR
Neighbor Joining trees
DARWIN
Factorial analyses
