# Supplementary material for: Naturally occurring variations in the nod-independent model legume Aeschynomene evenia and relatives: a resource for nodulation genetics
Source: BMC Plant Biol. 2018 Apr 3;18:54. doi: 10.1186/s12870-018-1260-2 (PMC5883870; doi:10.1186/s12870-018-1260-2)
Supplement: Supplementary file 9 — Figure S3. Detailed NJ trees representing the genetic diversity among the Nod-independent Aeschynomene accessions. The trees were developed separately in DARWIN using the allelic data of 65 SSRs for the 2× (a), 4× (b) and 6× (c) taxa. Well-differentiated taxa are distinctly colored. Identified genotypes are marked with a red dot and numbered. Accessions are designated with their LSTM code mentioned in Additional file 1: Table S1 followed by their geographical origin. Species suspected to be morphological variants are marked with an asterisk. Taxon colours and genotype numbers are the same as in Fig. 3. (PPTX 187 kb) [file 12870_2018_1260_MOESM9_ESM.pptx]

## Slide 1
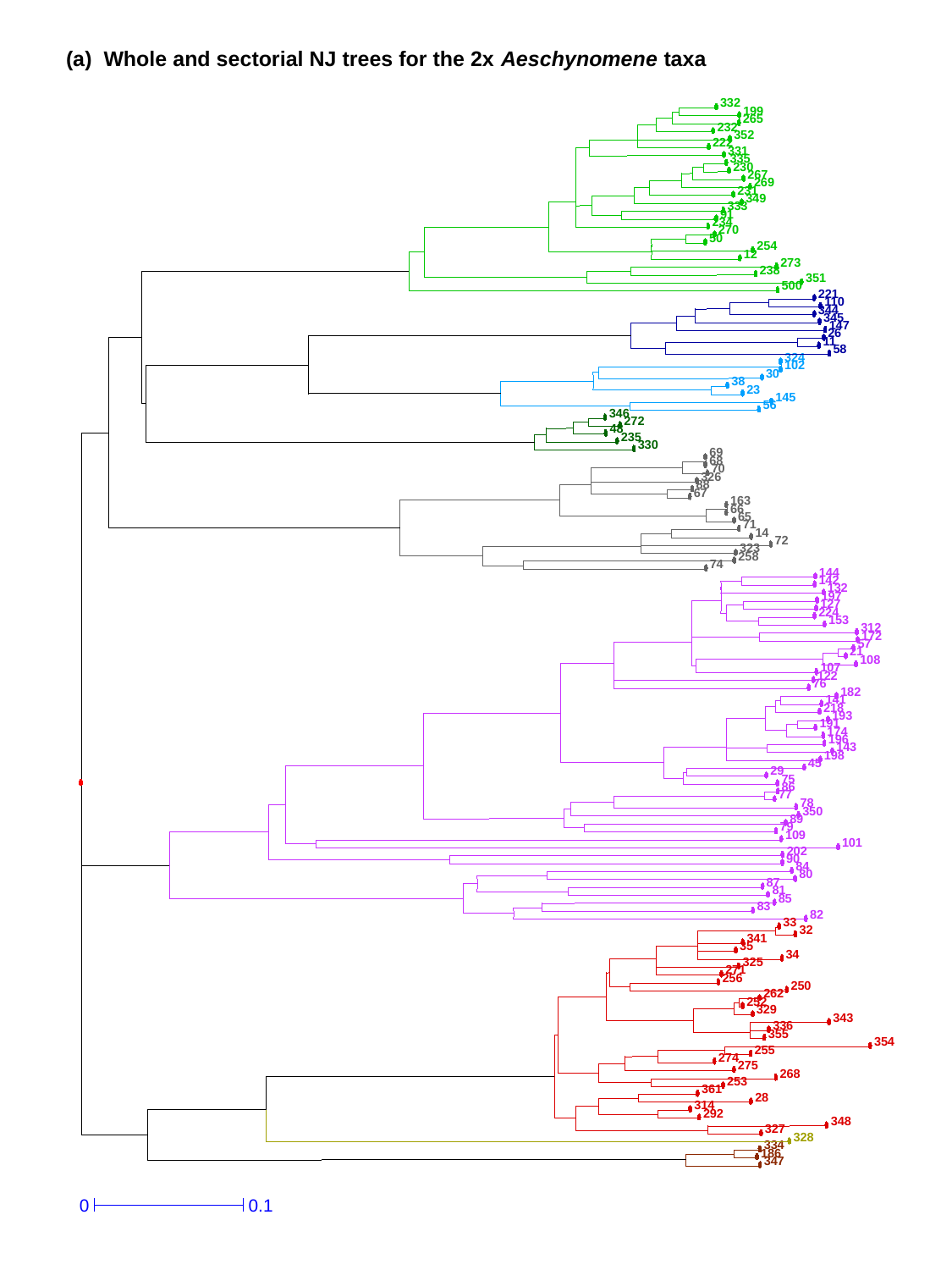

(a) Whole and sectorial NJ trees for the 2x Aeschynomene taxa
332
199
265
232
352
222
331
335
230
267
269
231
349
333
91
234
270
50
254
12
273
238
351
500
346
272
48
235
330
57
21
76
45
29
75
86
77
78
89
79
90
84
80
87
81
85
83
82
0.1
0
326
88
163
323
258
33
32
341
35
34
325
271
256
250
262
252
329
343
336
355
354
255
274
275
268
253
361
28
314
292
348
327
328
334
186
347
221
110
344
345
147
26
11
58
324
102
30
38
23
145
56
69
68
70
67
66
65
71
14
72
74
144
142
132
197
127
224
153
312
172
108
107
122
182
141
218
193
191
174
196
143
198
350
109
101
202

## Slide 2
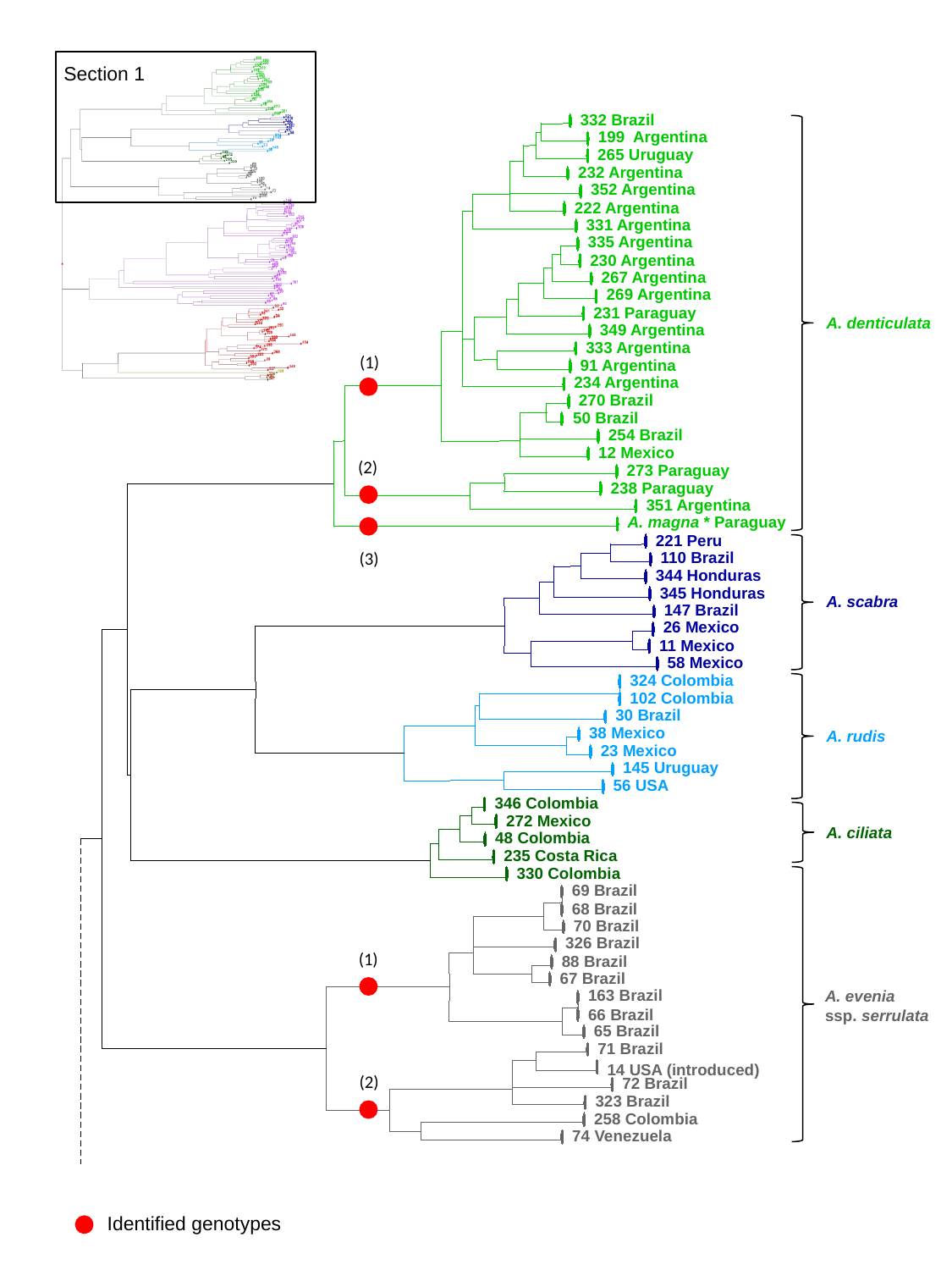

Section 1
332 Brazil
199 Argentina
265 Uruguay
232 Argentina
352 Argentina
222 Argentina
331 Argentina
335 Argentina
230 Argentina
267 Argentina
269 Argentina
231 Paraguay
A. denticulata
349 Argentina
333 Argentina
(1)
91 Argentina
234 Argentina
270 Brazil
50 Brazil
254 Brazil
12 Mexico
(2)
273 Paraguay
238 Paraguay
351 Argentina
A. magna * Paraguay
221 Peru
(3)
110 Brazil
344 Honduras
345 Honduras
A. scabra
147 Brazil
26 Mexico
11 Mexico
58 Mexico
324 Colombia
102 Colombia
30 Brazil
38 Mexico
A. rudis
23 Mexico
145 Uruguay
56 USA
346 Colombia
272 Mexico
A. ciliata
48 Colombia
235 Costa Rica
330 Colombia
69 Brazil
68 Brazil
70 Brazil
326 Brazil
(1)
88 Brazil
67 Brazil
163 Brazil
A. evenia ssp. serrulata
66 Brazil
65 Brazil
71 Brazil
14 USA (introduced)
(2)
72 Brazil
323 Brazil
258 Colombia
74 Venezuela
Identified genotypes

## Slide 3
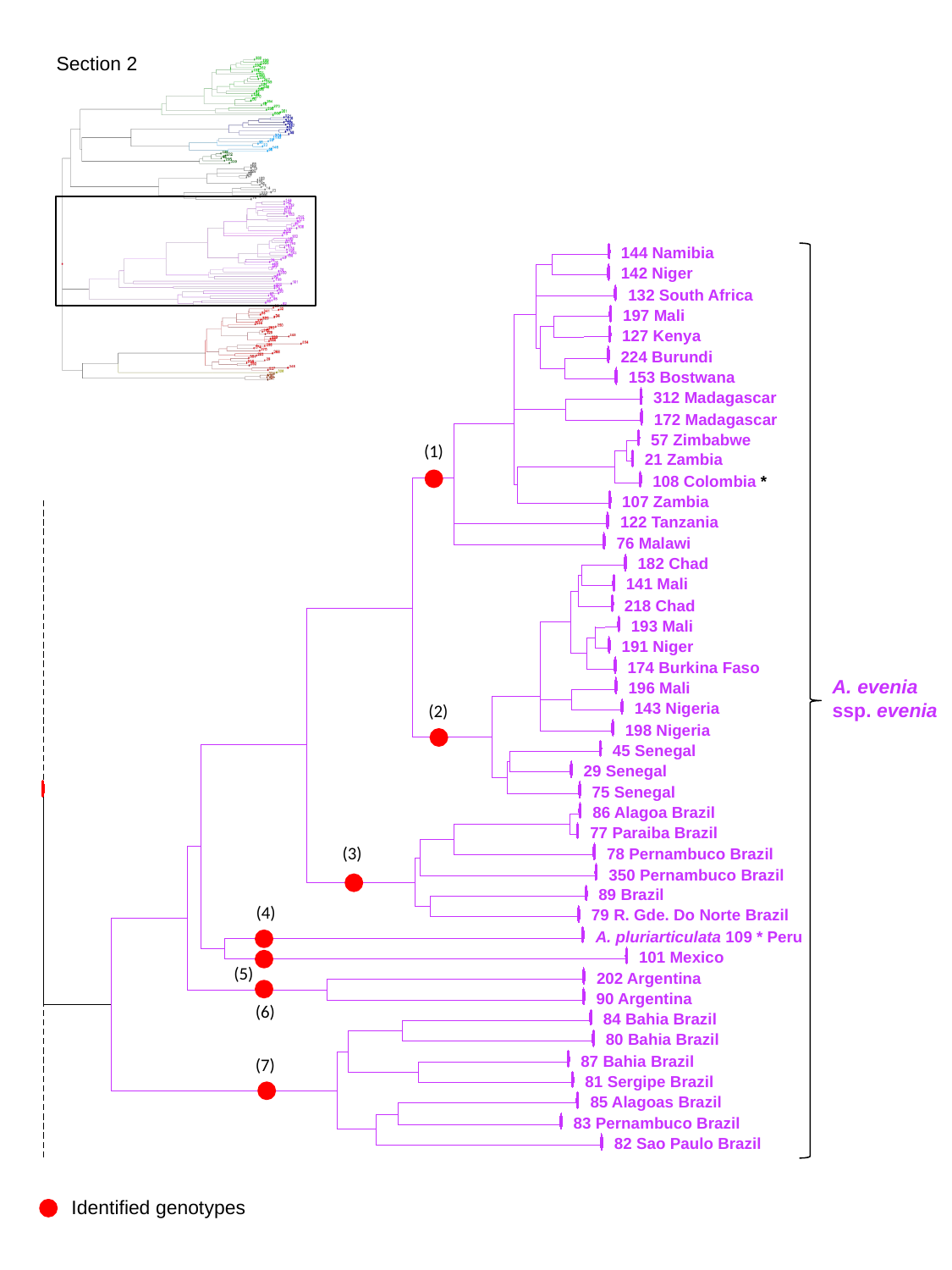

Section 2
144 Namibia
142 Niger
132 South Africa
197 Mali
127 Kenya
224 Burundi
153 Bostwana
312 Madagascar
172 Madagascar
57 Zimbabwe
(1)
21 Zambia
108 Colombia *
107 Zambia
122 Tanzania
76 Malawi
182 Chad
141 Mali
218 Chad
193 Mali
191 Niger
174 Burkina Faso
A. evenia ssp. evenia
196 Mali
(2)
143 Nigeria
198 Nigeria
45 Senegal
29 Senegal
75 Senegal
86 Alagoa Brazil
77 Paraiba Brazil
(3)
78 Pernambuco Brazil
350 Pernambuco Brazil
89 Brazil
(4)
79 R. Gde. Do Norte Brazil
A. pluriarticulata 109 * Peru
101 Mexico
(5)
202 Argentina
90 Argentina
(6)
84 Bahia Brazil
80 Bahia Brazil
(7)
87 Bahia Brazil
81 Sergipe Brazil
85 Alagoas Brazil
83 Pernambuco Brazil
82 Sao Paulo Brazil
Identified genotypes

## Slide 4
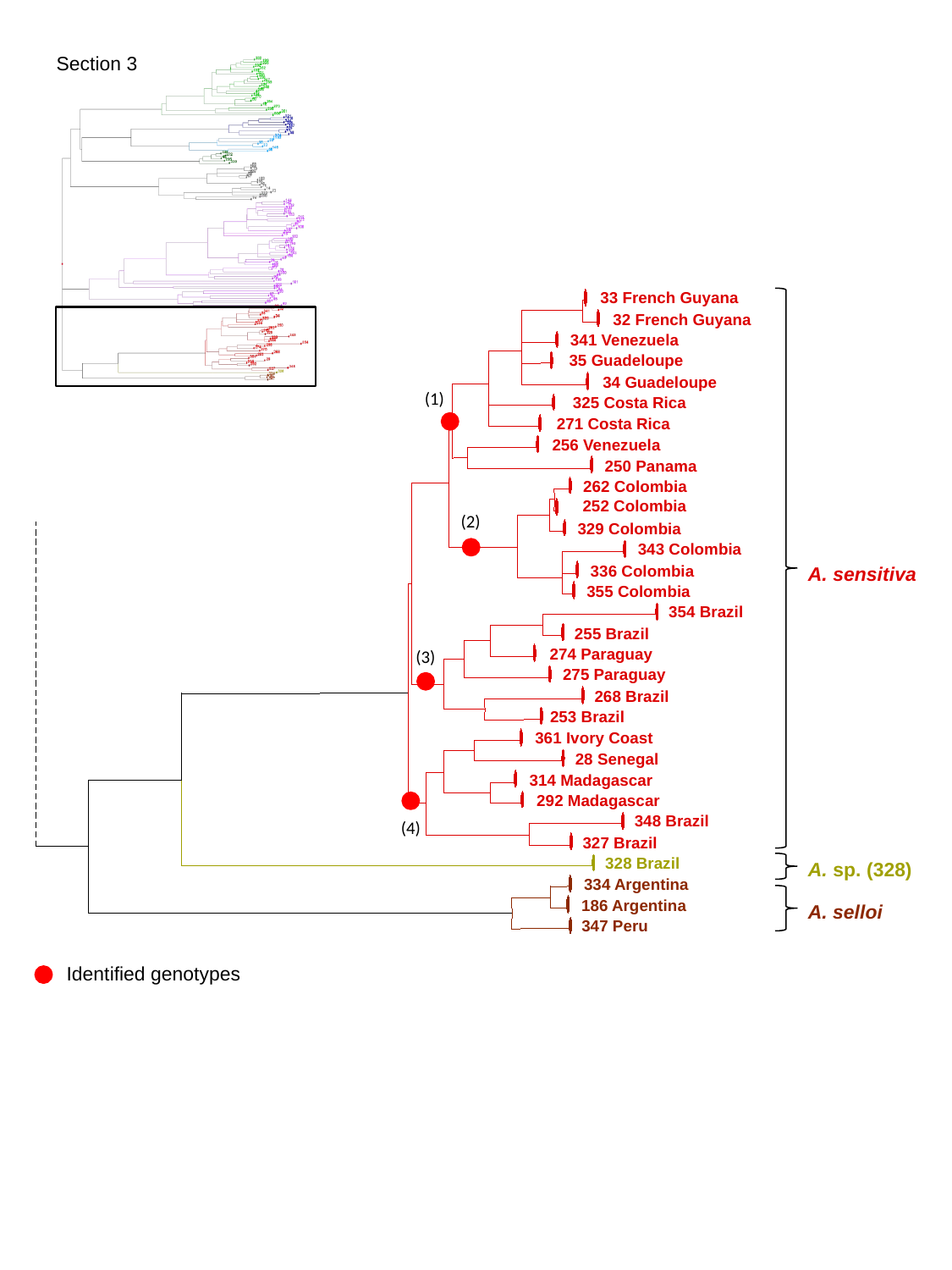

Section 3
33 French Guyana
32 French Guyana
341 Venezuela
35 Guadeloupe
34 Guadeloupe
325 Costa Rica
271 Costa Rica
256 Venezuela
250 Panama
262 Colombia
252 Colombia
329 Colombia
343 Colombia
336 Colombia
A. sensitiva
355 Colombia
354 Brazil
255 Brazil
274 Paraguay
275 Paraguay
268 Brazil
253 Brazil
361 Ivory Coast
28 Senegal
314 Madagascar
292 Madagascar
348 Brazil
327 Brazil
328 Brazil
A. sp. (328)
334 Argentina
186 Argentina
A. selloi
347 Peru
(1)
(2)
(3)
(4)
Identified genotypes

## Slide 5
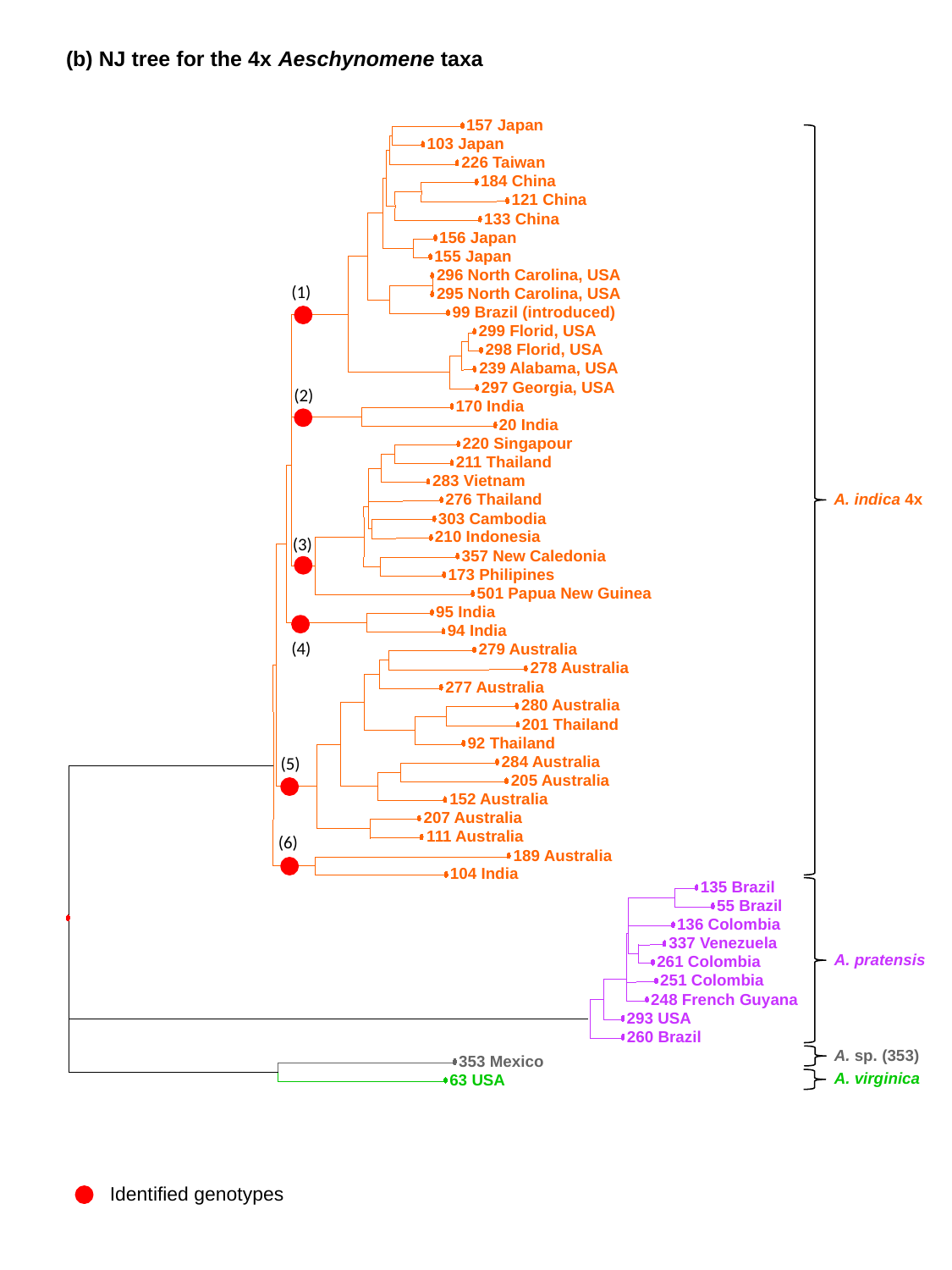

(b) NJ tree for the 4x Aeschynomene taxa
157 Japan
103 Japan
226 Taiwan
184 China
121 China
133 China
156 Japan
155 Japan
296 North Carolina, USA
(1)
295 North Carolina, USA
99 Brazil (introduced)
299 Florid, USA
298 Florid, USA
239 Alabama, USA
297 Georgia, USA
(2)
170 India
20 India
220 Singapour
211 Thailand
283 Vietnam
276 Thailand
A. indica 4x
303 Cambodia
(3)
210 Indonesia
357 New Caledonia
173 Philipines
501 Papua New Guinea
95 India
94 India
(4)
279 Australia
278 Australia
277 Australia
280 Australia
201 Thailand
92 Thailand
(5)
284 Australia
205 Australia
152 Australia
207 Australia
(6)
111 Australia
189 Australia
104 India
135 Brazil
55 Brazil
136 Colombia
337 Venezuela
A. pratensis
261 Colombia
251 Colombia
248 French Guyana
293 USA
260 Brazil
A. sp. (353)
353 Mexico
A. virginica
63 USA
Identified genotypes

## Slide 6
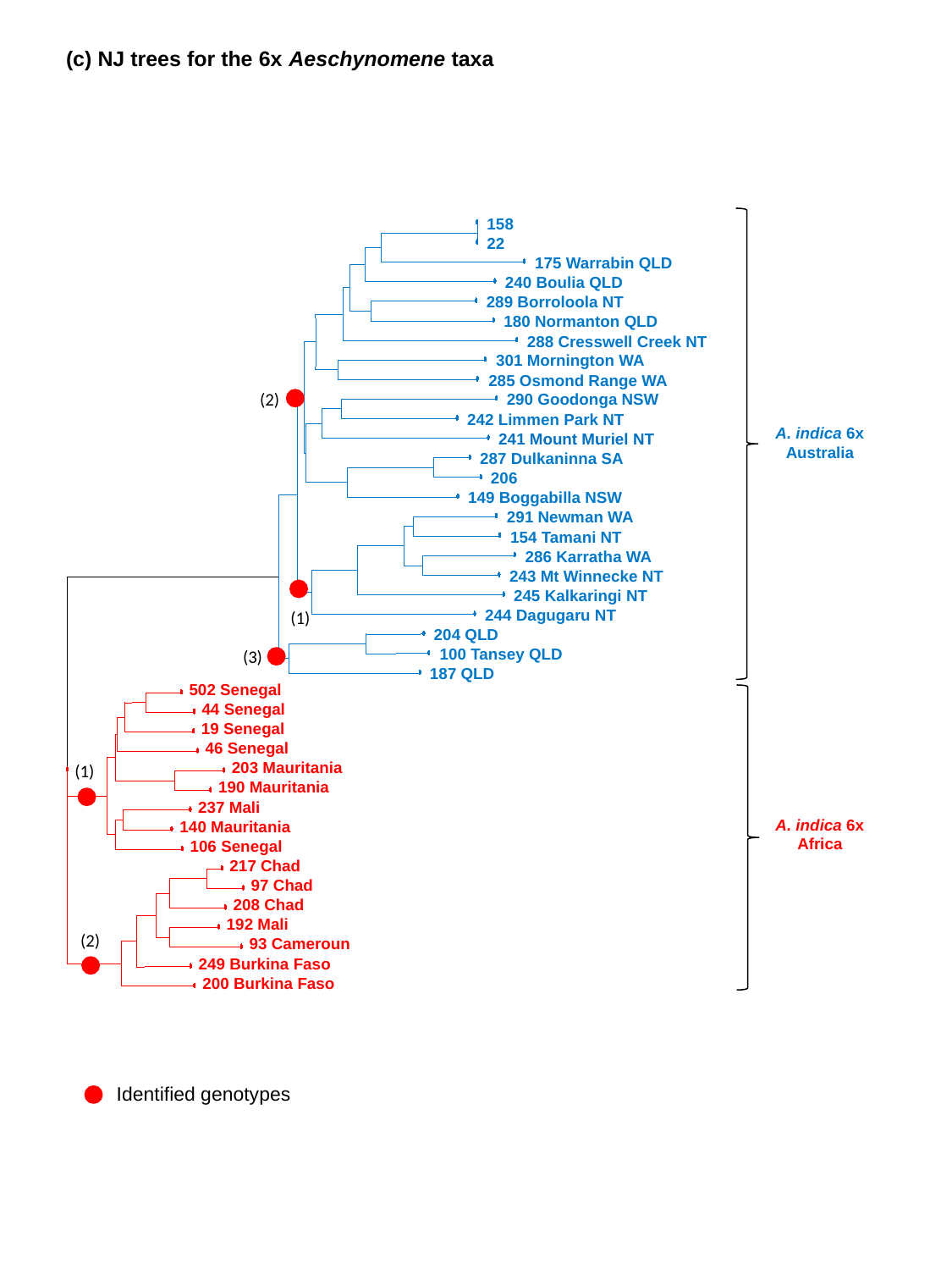

(c) NJ trees for the 6x Aeschynomene taxa
158
22
175 Warrabin QLD
240 Boulia QLD
289 Borroloola NT
180 Normanton QLD
288 Cresswell Creek NT
301 Mornington WA
285 Osmond Range WA
(2)
290 Goodonga NSW
242 Limmen Park NT
A. indica 6x Australia
241 Mount Muriel NT
287 Dulkaninna SA
206
149 Boggabilla NSW
291 Newman WA
154 Tamani NT
286 Karratha WA
243 Mt Winnecke NT
245 Kalkaringi NT
(1)
244 Dagugaru NT
204 QLD
(3)
100 Tansey QLD
187 QLD
502 Senegal
44 Senegal
19 Senegal
46 Senegal
(1)
203 Mauritania
190 Mauritania
237 Mali
A. indica 6x Africa
140 Mauritania
106 Senegal
217 Chad
97 Chad
208 Chad
192 Mali
(2)
93 Cameroun
249 Burkina Faso
200 Burkina Faso
Identified genotypes
