# Supplementary material for: Naturally occurring variations in the nod-independent model legume Aeschynomene evenia and relatives: a resource for nodulation genetics
Source: BMC Plant Biol. 2018 Apr 3;18:54. doi: 10.1186/s12870-018-1260-2 (PMC5883870; doi:10.1186/s12870-018-1260-2)
Supplement: Supplementary file 14 — Figure S4. Comparison of the nodulation properties of A. evenia s.s. and A. indica. Different accessions were root inoculated with Bradyrhizobium ORS278 and BTAi1 and analysed at 14dpi. (a) Number of nodules per accession. (b) Acetylene-reducing activity (ARA). A.U. Arbitrary Unit. Error bars represent s.d. (n = 6). (PPTX 179 kb) [file 12870_2018_1260_MOESM14_ESM.pptx]

## Slide 1
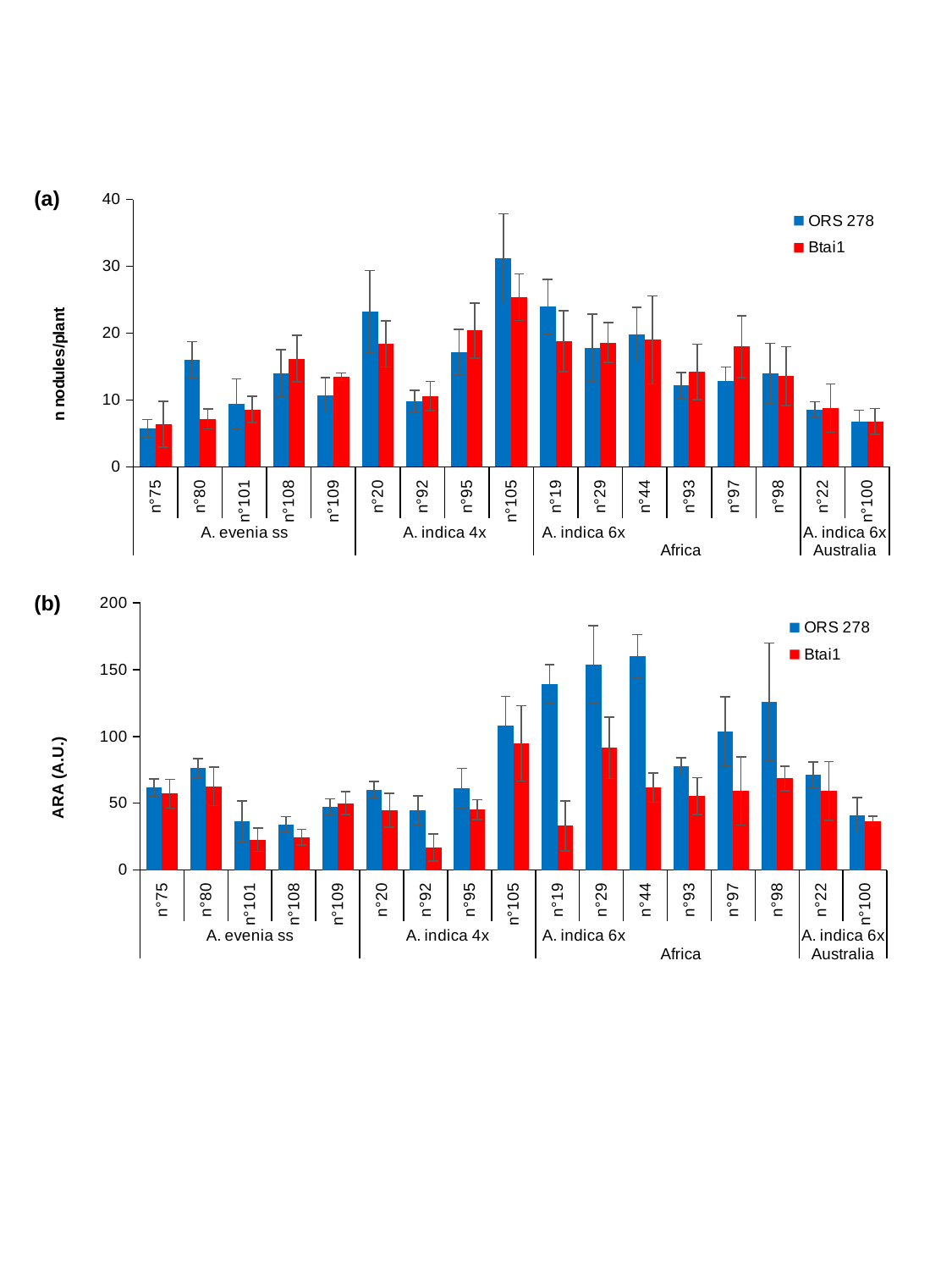

### Chart
| Category | ORS 278 | Btai1 |
|---|---|---|
| n°75 | 5.8 | 6.4 |
| n°80 | 16.0 | 7.2 |
| n°101 | 9.4 | 8.6 |
| n°108 | 14.0 | 16.2 |
| n°109 | 10.75 | 13.5 |
| n°20 | 23.2 | 18.4 |
| n°92 | 9.8 | 10.6 |
| n°95 | 17.2 | 20.4 |
| n°105 | 31.2 | 25.4 |
| n°19 | 24.0 | 18.8 |
| n°29 | 17.8 | 18.6 |
| n°44 | 19.8 | 19.0 |
| n°93 | 12.2 | 14.2 |
| n°97 | 12.8 | 18.0 |
| n°98 | 14.0 | 13.6 |
| n°22 | 8.6 | 8.8 |
| n°100 | 6.75 | 6.8 |(a)
### Chart
| Category | ORS 278 | Btai1 |
|---|---|---|
| n°75 | 61.7 | 57.13 |
| n°80 | 76.2 | 62.7 |
| n°101 | 36.47 | 22.6 |
| n°108 | 33.97 | 24.52 |
| n°109 | 47.24 | 50.05 |
| n°20 | 60.19 | 44.59 |
| n°92 | 44.64 | 16.81 |
| n°95 | 61.21 | 45.17 |
| n°105 | 108.1 | 94.8 |
| n°19 | 139.26 | 32.99 |
| n°29 | 153.9 | 91.52 |
| n°44 | 159.75 | 61.7 |
| n°93 | 77.58 | 55.32 |
| n°97 | 103.64 | 59.15 |
| n°98 | 125.96 | 68.49 |
| n°22 | 71.13 | 59.1 |
| n°100 | 41.05 | 36.68 |(b)
